# Prenatal cell-free DNA methylome detects association with autism and maternal obesity

**DOI:** 10.1038/s42003-026-10500-9

**Published:** 2026-06-22

**Authors:** Logan A. Williams, Ensieh Habibi, Meghan Miller, Irva Hertz-Picciotto, Cheryl K. Walker, Rebecca J. Schmidt, Janine M. LaSalle

**Affiliations:** 1 Department of Medical Microbiology and Immunology, University of California Davis School of Medicine, Davis, CA USA; 2 Department of Psychiatry and Behavioral Sciences, University of California Davis School of Medicine, Davis, CA USA; 3 Department of Public Health Sciences, University of California Davis School of Medicine, Davis, CA USA; 4 Department of Obstetrics and Gynecology, University of California Davis School of Medicine, Davis, CA USA; 5 Present Address: Center on Disability Studies, University of Hawai’i at Mānoa, Honolulu, HI USA

**Keywords:** Predictive markers, Autism spectrum disorders

## Abstract

Early identification of autism spectrum disorder (ASD) remains a critical challenge, particularly *in utero* when non-genetic factors such as maternal obesity (MO) are implicated. Here, we report results of whole-genome bisulfite sequencing of cell-free DNA (cfDNA) from third-trimester maternal plasma in a high-likelihood ASD pregnancy cohort associated with child (3 y) ASD diagnosis and/or MO. Differentially methylated regions (DMRs) between ASD and control cfDNA are strongly enriched for synaptic functions and genes previously implicated in ASD. These cfDNA ASD DMRs recapitulate those observed in ASD placenta and postmortem cortex and significantly overlap with MO DMRs. Our findings establish cfDNA methylation derived from maternal blood as a minimally invasive window into fetal brain ASD etiology, providing a framework for future mechanistic and early intervention studies. Future studies could investigate additional prenatal environmental exposures interacting with genetics during neurodevelopment.

## Abstract (web summary)

Prenatal cell-free DNA methylome shows overlapping associations between autism spectrum disorder (ASD) and maternal obesity, highlighting differential methylation of genes in synapse development pathways in the context of fetal brain ASD etiology.

## Introduction

In the United States, autism spectrum disorder (ASD) is diagnosed in 1 in 31 children, a figure representing a more than fourfold increase in prevalence over the last two decades^1,2^. Characterized by persistent deficits in social communication in combination with restrictive and repetitive patterns of behavior and interests, ASD is one of the most common neurodevelopmental disorders diagnosed in U.S children^3^. While ASD is variable in its severity and manifestation between individuals, children with ASD also show increased rates of attention deficit disorder, obesity, epilepsy, anxiety, depression, and intellectual disability^1,4–10^. However, effective early life behavioral interventions can dramatically improve quality of life outcomes for people with ASD^11–13^. Identifying early molecular indications of ASD may enable more effective early interventions and therapeutics, thereby improving the lives of people with ASD^14,15^.

The etiology of ASD development includes both genetic and environmental contributions^16–19^. Twin studies report 58–90% concordance among monozygotic twins, but ASD is highly polygenic, involving both rare large-effect and common small-effect variants^20–23^. To fully capture potential molecular predictors of ASD, it is necessary to also consider environmental exposures that may interact with genetic susceptibility to increase the likelihood of ASD development^24^. One known environmental factor is maternal obesity (MO), defined by having a pre-pregnancy BMI of ≥30 kg/m^2^ and currently estimated to affect up to 29% of pregnant women^25,26^. MO has been associated with a 36–54% increase in risk of ASD offspring^27–31^. MO is hypothesized to increase ASD risk though inflammation, specifically changes to proinflammatory cytokine levels, which cross the placenta and induce fetal neuroinflammation that dysregulates developmental gene regulatory networks^32–35^. Acting as the interface between environmental stimuli and gene network regulation, epigenetic mechanisms integrate environmental exposures like MO to gene networks contributing to ASD etiology^36^. Reproducible DNA methylated regions and signatures of ASD have been observed in the human brain and placenta^37–43^. MO in rhesus macaque studies revealed differential methylation in the infant brain, connecting MO to social behavior through gene networks enriched for functions in neurodevelopment^44^. Similar effects were observed in mice with perinatal high-fat diet exposure^45,46^.

Human studies on epigenetic ASD markers have been limited by the inaccessibility of fetal tissue prior to diagnosis. The onset of abnormal brain development and dysregulated gene expression profiles characteristic of ASD begins *in utero* as early as the 1st trimester, but due to the risks of invasive fetal sampling, research has historically been limited to investigating accessible perinatal tissues such as placenta and newborn blood^47–50^. However, advances in the field of noninvasive prenatal testing provide a means to examine fetal genetic material from cell-free fetal DNA (cffDNA) isolated from maternal blood^51,52^. Originating from developmental apoptosis in the placenta, cffDNA are predominantly short fetal-derived nucleic acids, representing a fraction of the total plasma cell-free DNA (cfDNA), coexisting with maternal circulating nucleic acids derived from hematopoietic cells and adipose tissue^53–60^. The fraction of cffDNA within cfDNA is variable between pregnant women, but increases throughout pregnancy and peaks at 21.6–41.0% in the 3rd trimester^61^. Notably, cffDNA retains a placental methylomic signature in maternal circulation, making it ideal for non-invasive testing of the fetal epigenome during pregnancy^62^. Some progress in assaying the prenatal cfDNA methylome has been made, though it has predominantly been constrained to lower-resolution methods, limited to individual case studies, or performed on pooled samples^62–65^.

To test the efficacy of cfDNA methylomes as environmentally sensitive predictors of ASD *in utero*, whole-genome bisulfite sequencing (WGBS) was performed on cfDNA isolated from maternal blood plasma in a high-likelihood prospective ASD cohort^66^. Comprehensive methylome analysis of cfDNA enabled the identification of differentially methylated regions (DMRs) of cfDNA associated with ASD or MO that were enriched for synaptic functions. These DMRs shared substantial similarity to ASD DMRs in the placenta and cortex, supporting the applicability of cfDNA methylation to the methylomes of other ASD relevant tissues.

## Results

### Study design and subject characteristics

To investigate fetal epigenomic signatures associated with ASD, we leveraged samples from the MARBLES cohort, a prospective longitudinal study of pregnancies at elevated ASD risk^66^. All participants contributed biological samples and detailed questionnaire data from preconception through early childhood, culminating in standardized neurodevelopmental assessments of all offspring at age 3 years.

**Fig. 1:**
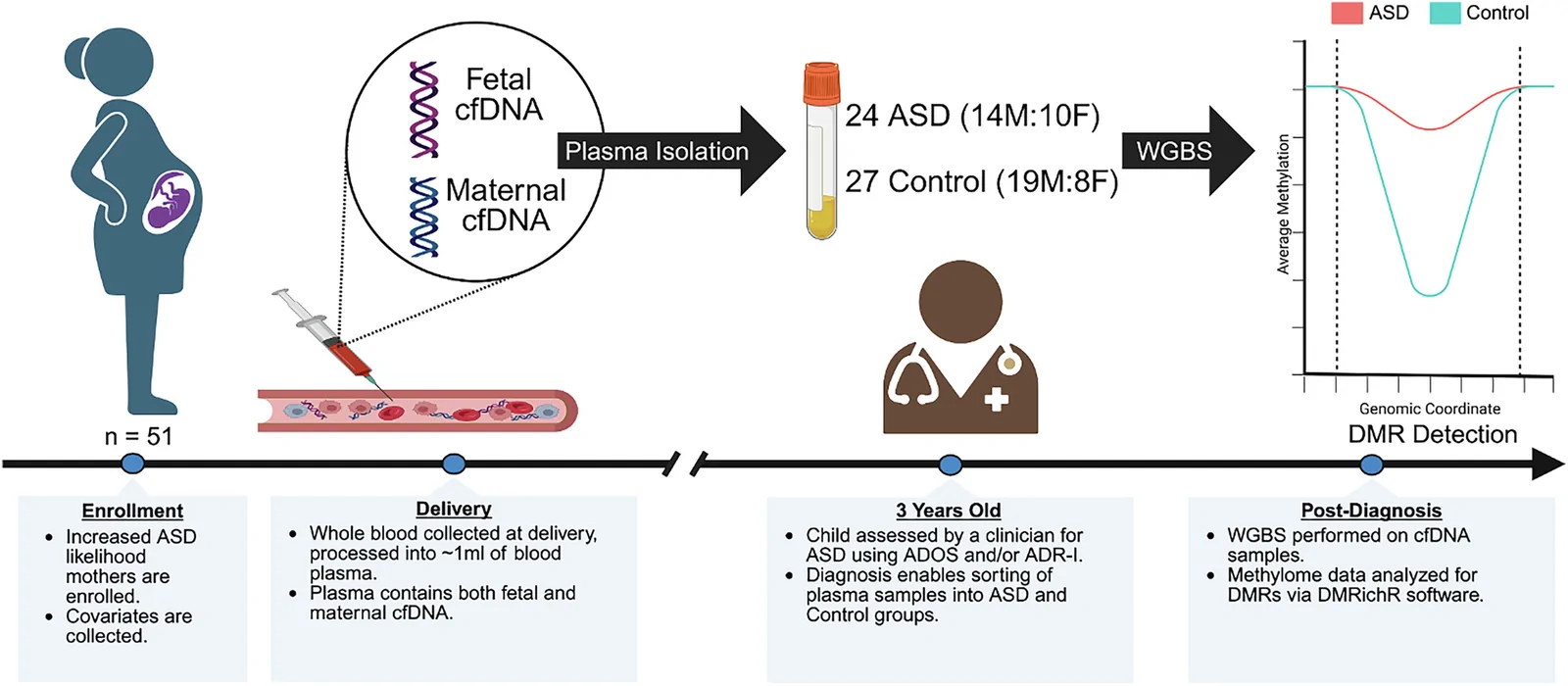
WGBS of cfDNA in a prospective autism cohort study enables prenatal detection of ASD-associated differentially methylated regions (DMRs). This experimental diagram details the process by which study participants were selected for cfDNA sampling, the subsequent diagnostic follow-up, and the computational method by which WGBS data was investigated for DMRs associated with ASD diagnosis. Created in BioRender. Williams, L. (2026) https://BioRender.com/p1nhmil.

A subset of 51 MARBLES pregnancies was selected to capture variability in fetal sex (*n* = 33 male; *n* = 18 female), maternal BMI (BMI; *n* = 33 control [BMI 18.5–29.8], *n* = 18 MO [BMI 30.2–51.2]) and ASD diagnosis (*n* = 27 non-ASD controls; *n* = 24 ASD) [Supplementary Data 1–4]. Our major inclusion criteria were the availability of 1 ml stored maternal plasma collected at delivery (36–41 weeks of gestation) from MARBLES participants with existing placental WGBS data to facilitate multi-tissue comparison [Supplementary Fig. 1]. CfDNA (4.1–37.4 ng) was isolated and subjected to WGBS, enabling methylome-wide interrogation of cfDNA at single-base resolution [Fig. 1].

### Fetal sex-stratified cfDNA methylomes reveal ASD-associated DMRs enriched in neurodevelopmental pathways

**Fig. 2:**
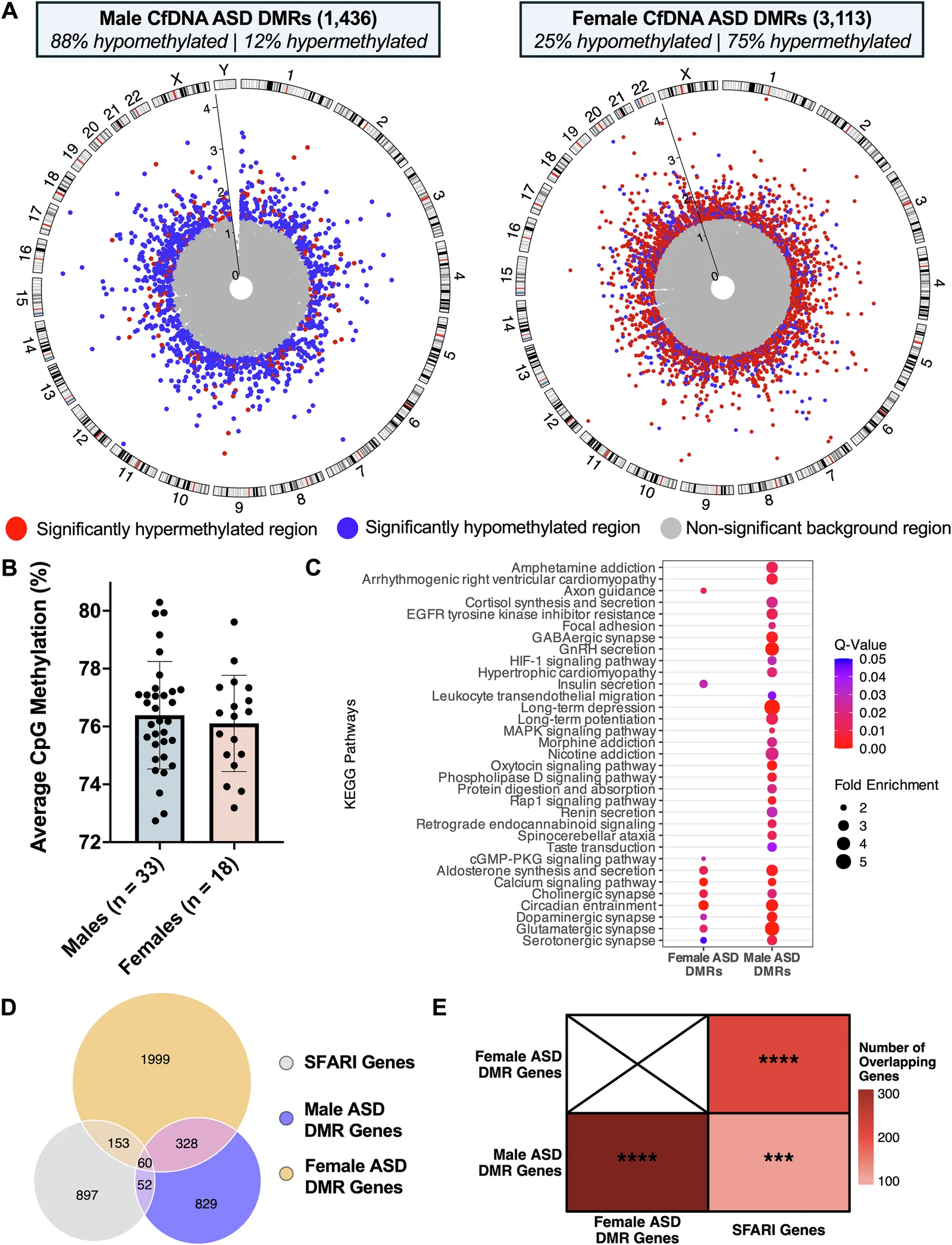
ASD DMRs are detectable in cfDNA and show heavy enrichment for synaptic gene pathways. **A** Circos plots of the identified ASD DMRs in cfDNA from sex-stratified analyses and plotted against the human genome. DMR directionality with respect to ASD diagnosis is denoted by dot color, with red indicating a hypermethylated DMR and blue indicating and hypomethylated DMR. Significance (-log_10_(permutation *p*-value)) of a given DMR is shown by its placement on the radial *y*-axis. **B** Bar plot average of global CpG methylation across all sequenced sites, separated by fetal sex (*n* = 33 males, *n* = 18 females). Error bars show standard deviation from the mean. No significant difference was identified between groups by application of a two-tailed *t*-test (*p* = 0.536). **C** Dot plot of all significant KEGG pathway enrichments for the male (*n* = 1269 male cfDNA ASD DMR genes) and female (*n* = 2540 female cfDNA ASD DMR genes) cfDNA ASD DMR genes. Significance indicated by dot color, and fold enrichment indicated by dot size. **D** Venn diagram of the overlapping genes between both sets of cfDNA ASD DMRs and the SFARI gene list. **E** Heatmap displaying the significance of gene overlap between both cfDNA ASD DMR gene lists (*n* = 1269 males, *n* = 2540 females) and the SFARI gene list (*n* = 1162 SFARI genes). Number of overlapping genes in each comparison is indicated by tile color. Significance of the overlap between two gene sets determined by Fisher’s Exact Test. Asterisks denote *p*-value as follows: ^***^p < 0.001, ^****^p < 0.0001.

DMRs between ASD and non-ASD controls after adjustment for MO were identified in a sex-stratified manner, yielding 1436 DMRs in male and 3113 in female pregnancies [Fig. 2A, and Supplementary Data 5 and 6]. ASD DMRs were detected in greater numbers in the female pregnancies than the male pregnancies, and also displayed a significantly greater magnitude of change [Supplementary Fig. 2], consistent with what we have previously observed for ASD DMRs from newborn blood spots and cord blood^41,42^. Many ASD DMRs displayed opposing methylation directionality by fetal sex, with male DMRs skewed towards hypomethylation and female DMRs towards hypermethylation, a pattern not observed at the global methylome level [Fig. 2B]. Combining both sexes and correcting for sex reduced ASD DMR detection sensitivity, despite greater power, resulting in only 377 DMRs that exhibited no significant enrichment for expected neuronal gene pathways [Supplementary Data 8, and Supplementary Fig. 3]. Despite sex-specific directionality, both male and female ASD DMRs were similarly enriched in genic (promoters and exons) versus intergenic regions and preferentially localized to CpG-rich regions [Supplementary Fig. 4].

Genes mapped to these DMRs were analyzed for KEGG pathway enrichment. Male ASD DMRs were enriched for 30 pathways and female DMRs for 10, with 7 shared pathway enrichments across both groups [Fig. 2C, and Supplementary Data 9 and 10]. These included multiple neuronal synaptic pathways such as glutamatergic, serotonergic, and cholinergic synapses; calcium signaling; and circadian entrainment. Gene ontology enrichment analysis corroborated these findings, though with greater divergence between sexes [Supplementary Fig. 5].

### CfDNA ASD DMR genes are enriched for known autism risk genes and developmentally dynamic loci

Both sex-specific ASD DMR gene lists significantly overlapped with the SFARI Gene database [Fig. 2D, E], with 112 genes in common for males and 213 for females, though fetal fraction-adjusted female cfDNA DMRs did not replicate this significant SFARI overlap [Supplementary Fig. 6]. Male ASD DMRs were selectively enriched for SFARI genes categorized as “strong candidate” genes, while Female ASD DMRs exhibited enrichment for both SFARI “high confidence” and “strong candidate” genes [Supplementary Data 11]. The two gene lists were also significantly enriched for each other (388 overlapping genes), and their DMRs showed significant proximity in the genome beyond random expectation [Supplementary Fig. 7]. Notably, 60 genes appeared in both cfDNA ASD sets and the SFARI database, including prominent autism risk genes such as *ANK2*, *CACNA1C*, and *GRIN2B* [Fig. 2D, and Supplementary Data 12]^67–69^.

DMR genes were also enriched for loci undergoing dynamic methylation changes across pregnancy [Supplementary Fig. 8], suggesting temporal relevance^70^. Enrichment was further observed in Polycomb-repressed and weakly repressed chromatin states, including those in brain and placental tissues [Supplementary Fig. 9, and Supplementary Data 13 and 14], indicating potential developmental regulation consistent with neurodevelopment risk genes like *AUTS2*^71–74^.

### CfDNA methylation patterns mirror ASD-associated DMRs in placenta and cortex

**Fig. 3:**
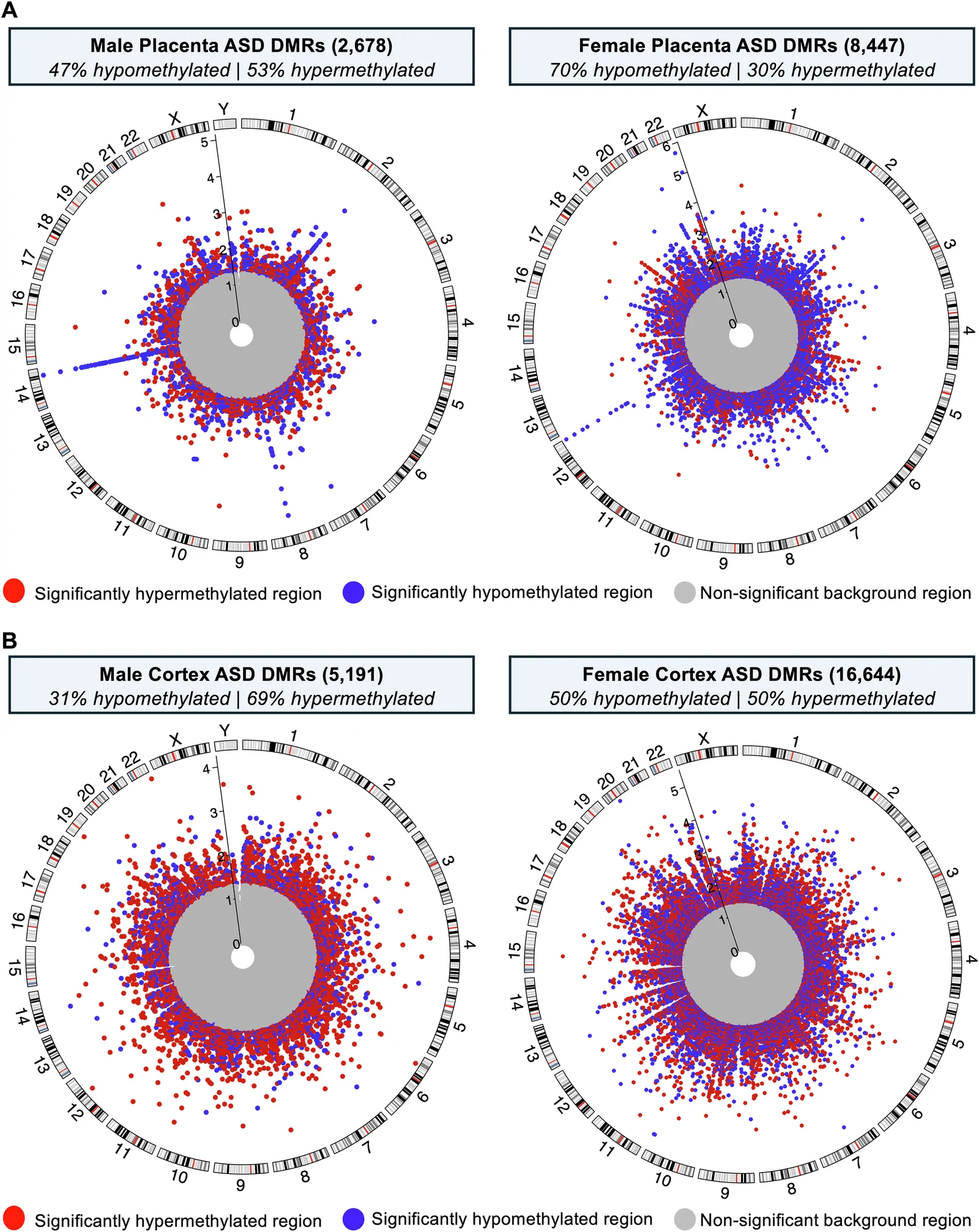
ASD DMRs identified from placenta and cortex WGBS exhibit shared characteristics with cfDNA DMRs. Circos plots mapping the genomic locations and significance of all DMRs from the sex-stratified analyses of **A** placenta and **B** cortex WGBS. DMR directionality with respect to ASD diagnosis is denoted by dot color, with red indicating a hypermethylated DMR and blue indicating and hypomethylated DMR. Significance (-log_10_(permutation *p*-value)) of a given DMR is shown by its placement on the radial *y*-axis.

To assess the tissue relevance of cfDNA methylation patterns, we compared cfDNA ASD DMRs to those identified from previous WGBS data from matched term placenta samples from MARBLES, as well as WGBS from unrelated ASD and control post-mortem cortex^37,39^. Using identical DMR pipelines as for cfDNA, we identified 2678 DMRs (male) and 8447 DMRs (female) in the placenta, and 5191 DMRs (male) and 16,644 DMRs (female) in the cortex [Fig. 3A, B, and Supplementary Data 15–18].

**Fig. 4:**
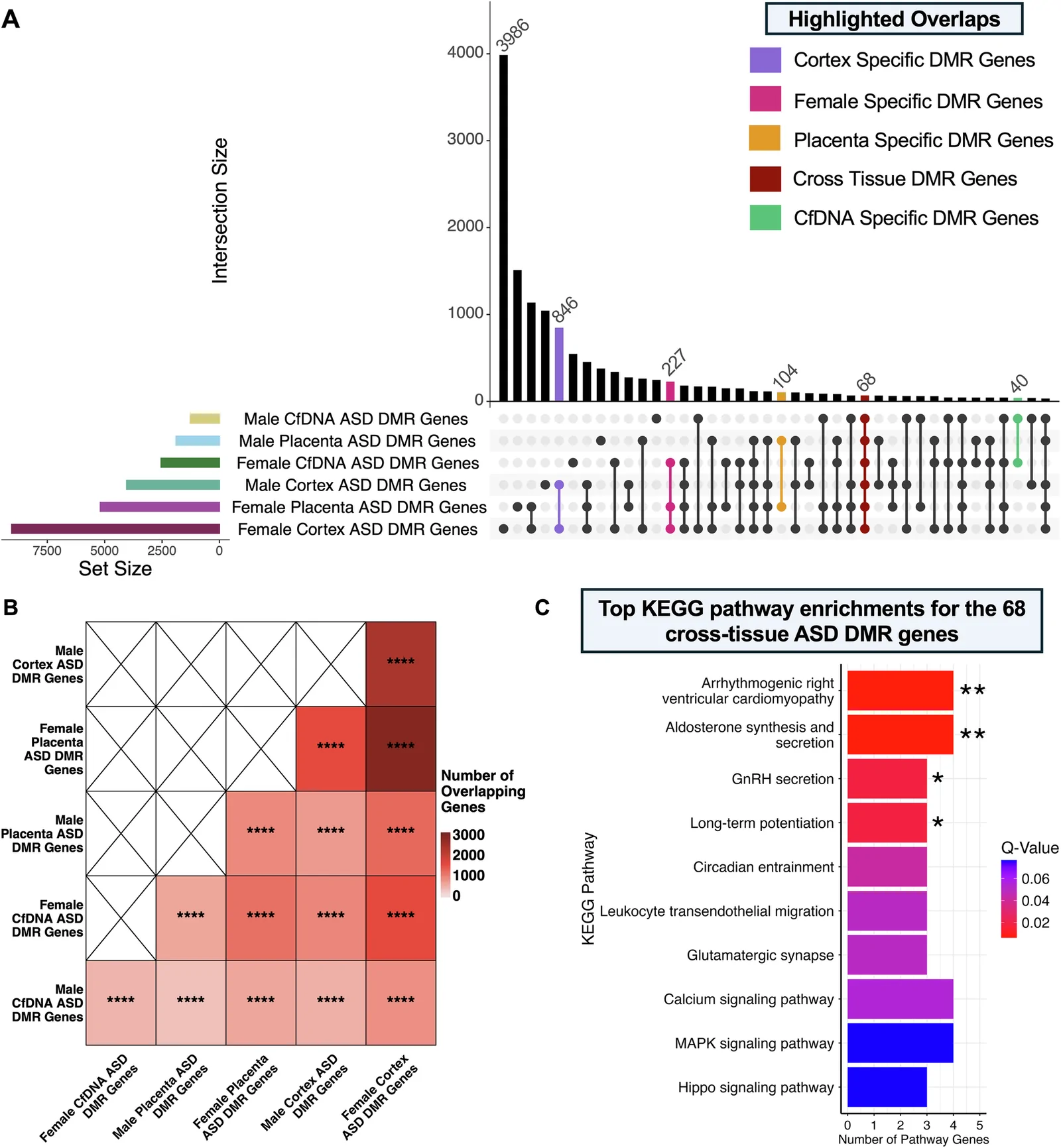
ASD DMRs across tissues overlap with one another. **A** Upset plot of the intersections between the six ASD DMR genes lists. Some intersections of interest are highlighted with specified colors and labelled for the number of overlapping genes between the sets in question. **B** Heatmap of the significance of gene overlap between the six DMR gene lists (*n* = 1269 male cfDNA, *n* = 2540 female cfDNA, *n* = 1891 male placenta, *n* = 5189 female placenta, *n* = 4082 male cortex, *n* = 9049 female cortex). Significance of the overlap between two gene sets determined by Fisher’s Exact Test. Asterisks denote *p*-value as follows: ^****^p < 0.0001. **C** Bar graph of the top 10 most significantly enriched KEGG pathways for the list of 68 genes that appear in all six ASD DMR gene lists. Significance determined via Fisher’s Exact Test with Benjamini–Hochberg correction. Asterisks denote *q*-value as follows: ^*^q < 0.05, ^**^q < 0.01.

All tissues shared a uniform genomic distribution of DMRs and consistent enrichment in gene bodies and CpG islands [Supplementary Figs. 10 and 11], though the fetal sex difference in methylation directionality observed in cfDNA was less pronounced in the placenta and cortex than was observed for cfDNA [Fig. 2A]. Cross-tissue gene-level overlap was substantial, with all six ASD DMR gene lists showing significant intersections [Fig. 4A, B, and Supplementary Figs. 12–17]. KEGG analysis across all DMR gene sets identified four pathways that were enriched in all six tissue/sex groups: glutamatergic synapse, cholinergic synapse, calcium signaling, and circadian entrainment [Supplementary Figs. 18–20], pointing to convergent methylomic alteration in synaptic gene pathways implicated across tissues and sexes.

A shared set of 68 genes was present in all six tissue/sex differentially methylated gene lists [Supplementary Data 19]. These were enriched for pathways including long-term potentiation, GnRH signaling, circadian entrainment, and aldosterone synthesis [Fig. 4C, and Supplementary Data 20]. The novel gene neuronal hypoxia inducible, placenta-associated (*NHIP*) that we previously identified as ASD associated from MARBLES placenta was included in this cross-tissue/sex differentially methylated gene list, consistent with its described function in both placenta and brain^39^. No ASD DMR genes were shared among male-only DMR lists, but 227 were exclusive to female-specific DMRs, encompassing known risk loci such as *AUTS2*, *NRCN1*, and *SCN2A*, although no KEGG enrichments were identified for this female-specific set [Supplementary Data 21, and Supplementary Fig. 21]. All placenta and cortex DMR gene lists exhibited significant enrichment for SFARI genes [Supplementary Figs. 22 and 23]. Among the 227 female-specific cross-tissue DMR-associated genes, 20 overlapped with established SFARI risk genes. Notably, five of these exhibited DMRs consistently localized within identical genic features and demonstrated concordant methylation alterations across all three tissues [Table 1]. This persistent, cross-tissue epigenetic signature is specifically associated with ASD diagnosis in females and is detectable prenatally in cfDNA.

**Table 1:** Summary of DMRs in the five genes from the female-specific cross-tissue ASD DMR gene list which have concordant genic annotation and methylation directionality

Gene	SFARI gene score	Annotation	Direction	DMR Positions	Tissue	% methylation difference	*P*-value
NRXN2	1	Intron	Hypermethylated	chr11:64,637,874-64,637,983	CfDNA	14	0.0476
				chr11:64,711,585-64,712,063	Placenta	10	0.0179
				chr11:64,611,402-64,612,258	Cortex	18	0.0103
				chr11:64,613,813-64,614,998	Cortex	15	0.0183
				chr11:64,645,890-64,646,102	Cortex	19	0.0203
ERG	2	Intron	Hypermethylated	ch21:38,436,804-38,437,438	CfDNA	12	0.0141
				chr21:38,384,196-38,384,517	Placenta	9	0.0460
				chr21:38,395,695-38,395,950	Cortex	17	0.0270
				chr21:647,306-38,648,469	Cortex	15	0.0458
PTBP2	2	Distal Intergenic	Hypermethylated	chr1:96,919,158-96,919,901	CfDNA	12	0.0075
				chr1:96,833,633-96,834,747	Placenta	10	0.0397
				chr1:96,592,914-96,594,274	Cortex	21	0.0132
SF3B1	2	Distal Intergenic	Hypermethylated	chr2:197,371,759-197,372,113	CfDNA	13	0.0057
				chr2:197,369,767-197,370,324	Placenta	11	0.0343
				chr2:197,368,831-197,369,767	Cortex	23	0.0014
TBX22	3	Distal Intergenic	Hypomethylated	chrX:79,843,148-79,845,059	CfDNA	-14	0.0091
				chrX:79,956,019-79,957,534	Placenta	-8	0.0409
				chrX:80,139,730-80,139,990	Cortex	-18	0.0195
				chrX:79,749,789-79,750,252	Cortex	-30	0.0369

Each entry specifies the DMR gene, SFARI gene score, genic annotation, methylation directionality, chromosomal coordinates, tissue source, % methylation difference between ASD and Control samples, and the *p*-value of the DMR call. These loci may represent a female-specific cross-tissue epigenetic signature of ASD detectable early in development.

### MO DMRs in cfDNA are detectable in ASD relevant gene pathways and enriched in ASD DMRs of both fetal sexes

The identification of ASD-associated methylation signatures in cfDNA provides a foundation for exploring convergent epigenetic mechanisms linking ASD with a common prenatal exposure, MO. To explore this hypothesis, we identified DMRs on the same group of cfDNA WGBS but stratified by MO rather than ASD. MO was defined categorically, with a pre-pregnancy BMI of 30 kg/m^2^ separating obese mothers (*n* = 18, 9 females and 9 males, 9 non-ASD and 9 ASD) from non-obese mothers (*n* = 33, 9 females and 24 males, 18 non-ASD and 15 ASD).

**Fig. 5:**
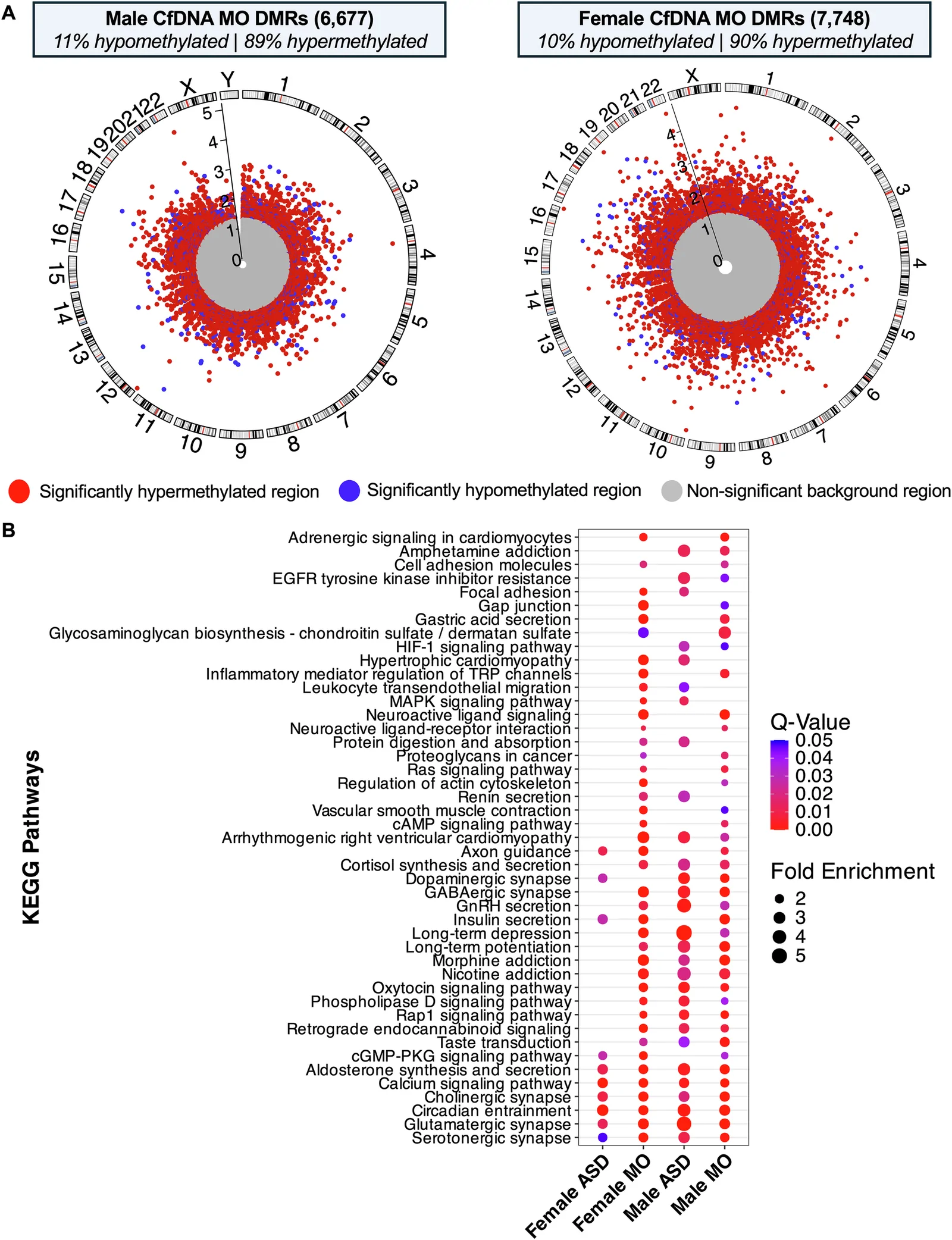
MO DMRs in cfDNA share substantial overlaps with ASD DMRs, while maintaining unique enrichments for inflammatory pathways. **A** Circos plots mapping the genomic locations and significance of all MO DMRs from the sex-stratified analyses of cfDNA WGBS. **B** Dot plot of all significant KEGG pathway enrichments for cfDNA ASD and MO DMRs (*n* = 1269 male cfDNA ASD DMR genes, *n* = 2540 female cfDNA ASD DMR genes, *n* = 3967 male cfDNA MO DMR genes, *n* = 4887 female cfDNA MO DMR genes). Significance (-log_10_(permutation *p*-value)) of a given DMR is shown by its placement on the radial *y*-axis. Fold enrichment is indicated by dot size.

We identified 6,677 MO DMRs in cfDNA from male pregnancies and 7748 MO DMRs in female pregnancies [Supplementary Data 22 and 23]. Samples were stratified by fetal sex to maintain comparability with sex-specific ASD DMR profiles. Unlike ASD DMRs, MO DMRs did not display marked differences in global methylation directionality between sexes [Fig. 5A]. Like ASD findings, MO DMRs exhibited preferential localization within gene bodies and promoters, but de-enrichment over intergenic regions [Supplementary Fig. 24].

To assess shared genomic features, we compared MO and ASD DMRs from cfDNA by intersecting their genomic coordinates within matched sex strata. This analysis revealed highly significant overlap in both male and female MO and ASD DMRs comparisons relative to permutation-based expectations using matched control region sets [Supplementary Figs. 25 and 26]. Functional enrichment of DMR-associated genes via Kyoto Encyclopedia of Genes and Genomes (KEGG) pathway analysis revealed six pathways consistently enriched across all four cfDNA DMR datasets: aldosterone synthesis and secretion (hsa04925), calcium signaling (hsa04020), cholinergic synapse (hsa04725), circadian entrainment (hsa04713), glutamatergic synapse (hsa04724), and serotonergic synapse (hsa04726) [Fig. 5B]. These pathways reflect convergent epigenetic alterations within neurodevelopmentally critical signaling networks implicated in both MO and ASD contexts. Additional enrichments unique to MO DMRs, such as inflammatory mediator regulation of TRP channels (hsa04750), suggest a broader engagement of immune and regulatory systems in MO exposure compared to ASD.

**Fig. 6:**
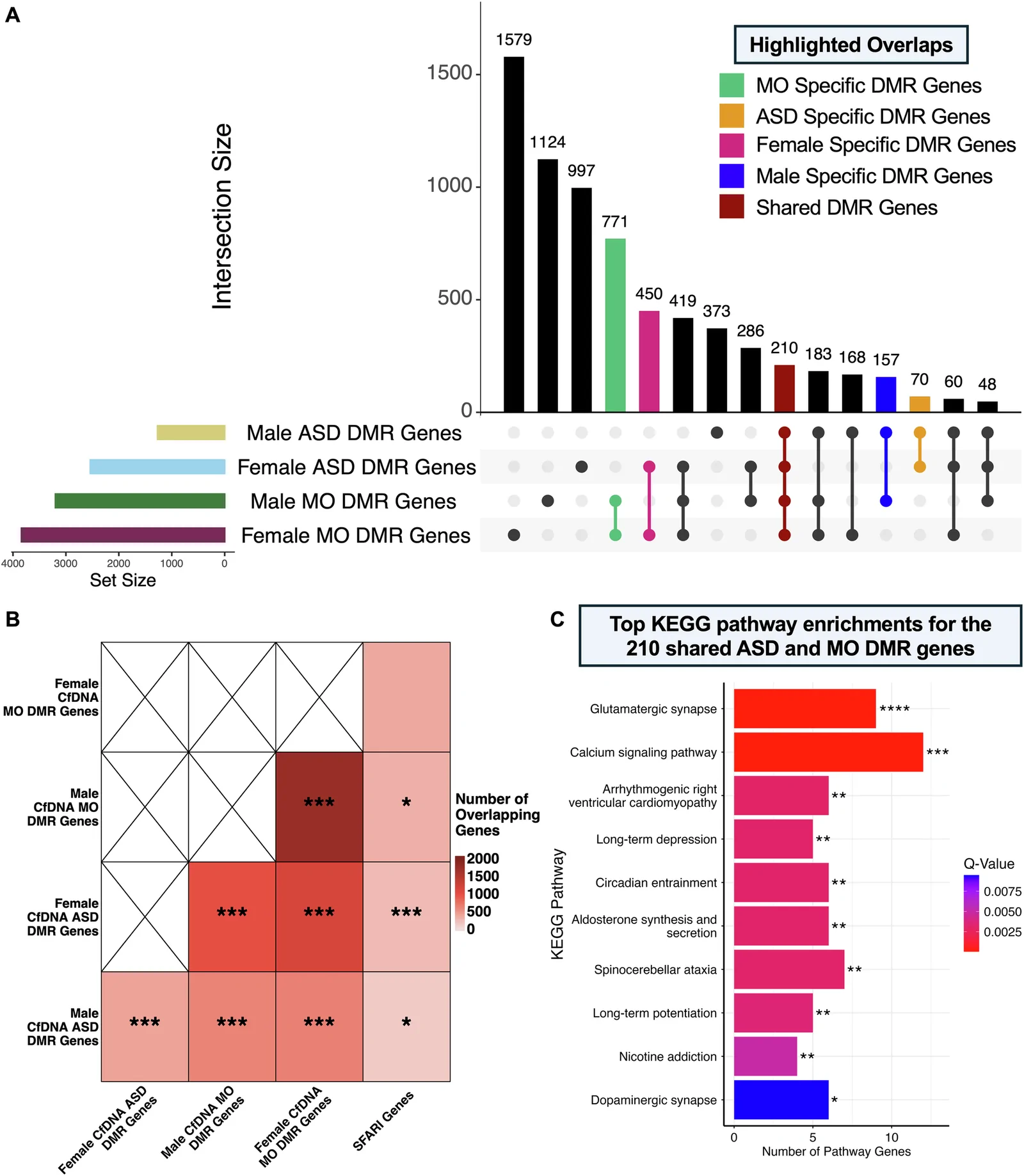
Intersecting ASD and MO DMRs are substantially enriched for genes in neurodevelopmental gene pathways. **A** Upset plot of gene overlaps between cfDNA ASD and MO DMRs. Some intersections of interest are highlighted in specified colors. **B** Heatmap of the significance of gene overlap between the cfDNA DMR gene lists (*n* = 1269 male cfDNA ASD DMR genes, *n* = 2540 female cfDNA ASD DMR genes, *n* = 3967 male cfDNA MO DMR genes, *n* = 4887 female cfDNA MO DMR genes) and the SFARI gene list (*n* = 1162 SFARI genes). Significance of the overlap between two gene sets determined by Fisher’s Exact Test. Asterisks denote *p*-value as follows: ^***^p < 0.001, ^*^p < 0.05. **C** Bar graph of the top 10 most significantly enriched KEGG pathways for the list of 210 genes that appear in all cfDNA DMR gene lists. Significance determined via Fisher’s Exact Test with Benjamini–Hochberg correction. Asterisks denote *q*-value as follows: ^****^q < 0.0001, ^***^q < 0.001, ^**^q < 0.01, ^*^q < 0.05.

To further delineate trait-specific and shared epigenetic signatures, we examined the intersection of the four cfDNA DMR gene lists corresponding to ASD and MO [Fig. 6A]. Pairwise comparisons using Fisher’s Exact Test revealed significant mutual enrichment among all four DMR gene lists [Fig. 6B]. Notably, three of the four DMR gene lists exhibited significant overlap with the SFARI Gene database, with only the female MO DMR gene list not passing our significance threshold. However, female cfDNA MO DMRs with adjustment for estimated fetal fraction do display significant overlap with the SFARI Gene database [Supplementary Fig. 6]. When stratified by SFARI Gene evidence thresholds, Male MO DMRs were selectively enriched for “strong candidate” SFARI genes, while female MO DMRs approached significant enrichment for the same SFARI subcategory [Supplementary Data 11].

To resolve trait-specific biological associations, KEGG pathway enrichment analysis was performed separately on genes unique to either MO or ASD DMRs. Among the 922 genes specific to MO DMRs, significant enrichment was observed for pathways involved in morphine addiction (hsa05032) and neuroactive ligand signaling (hsa04080), implicating neuromodulatory signaling systems in the context of maternal metabolic influence [Supplementary Fig. 27]. By contrast, analysis of the 70 genes uniquely associated with ASD DMRs identified enrichment for pathways related vitamin digestion and absorption (hsa04977), pancreatic secretion (hsa04972), and motor proteins (hsa04814), suggesting an alternative axis of neurodevelopmental disruption [Supplementary Fig. 28].

Finally, we interrogated the 210 genes common to all four cfDNA DMR lists to identify shared epigenetically perturbed pathways [Supplementary Data 24]. KEGG analysis of these overlapping genes revealed significant enrichment across 15 pathways [Fig. 6C], including glutamatergic synapse (hsa04724), circadian entrainment (hsa04713), and calcium signaling pathway (hsa04020), three of the four pathways consistently identified as enriched in the ASD DMR sets across multiple tissue types, highlight convergent neurodevelopmental mechanisms across etiologically distinct maternal exposures and fetal outcomes.

## Discussion

Our study presents evidence that cfDNA collected from late 3rd-trimester maternal blood reflects epigenomic signatures associated with ASD diagnosis. Remarkably, we demonstrate that cfDNA methylation patterns distinguishing ASD from non-ASD are sex-specific and occur at genes with functions at fetal neuronal synapses, despite originating from maternal blood. ASD DMRs overlapped across cfDNA, matched placenta, and postmortem cortex, reinforcing that ASD etiology begins *in utero* and converges on placental-brain pathways. WGBS of cfDNA identified ASD and MO-associated DMRs, uncovering both distinct and convergent molecular pathways across sexes. These findings advance our understanding of ASD etiology as being rooted in early developmental dysregulation, emphasizing maternal metabolic health as a modifiable factor that may shape the prenatal epigenomic landscape in ways that modulate synaptic development.

Epigenetic modifications, particularly DNA methylation, mediate gene-environment interactions during fetal development. Our detection of ASD-associated DMRs in the 3rd trimester cfDNA—before the manifestation of behavioral symptoms—supports a model in which prenatal epigenetic programming contributes to ASD susceptibility. These ASD DMRs were enriched in genes involved in synaptic signaling, transcriptional regulation, and neuronal connectivity, consistent with prior sex-combined studies of post-mortem ASD brain tissues, and with reports of fetal origins of neurodevelopmental conditions^75–77^. These pathway enrichments are consistent with a contemporary understanding of ASD as developing from systemic alteration to general neurodevelopmental pathways^78^. Epigenetic profiles of ASD in cfDNA appear to mirror genetic profiles of ASD in this regard, as they implicate numerous loci with convergent neurodevelopmental relevance as opposed to singular high-penetrance variants^79–81^. ASD etiology is characterized by the cascading effects of alterations to synaptic development, brain energy and metabolism, inflammatory response, and myriad other biological pathways acting in concert to produce a broad neurodevelopmental phenotype, and our reported ASD DMRs in maternal cfDNA reflect this diverse etiology.

Substantial sex-specific divergence in ASD-associated methylation signatures aligns with male-biased ASD prevalence and with sex-specific placenta and fetal epigenomic regulation^82,83^. Particularly in cfDNA, we observe substantial sex differences in the directionality of methylation associated with ASD, though the downstream effects of such differences are difficult to predict. The subset of cfDNA DMRs in genes shared between males and females with diverging methylation patterns at the same genic elements shows a significant enrichment for the MAPK signaling pathway (hsa04010), potentially implicating altered MAPK signaling as interacting with fetal sex [Supplementary Fig. 29A]. MAPK signaling pathways are rhythmic in the developing brain and function to entrain the brain’s activity to maternal feeding and activity cues^84,85^. MAPK and circadian entrain pathways are also known to be sexually dimorphic^86,87^. In contrast, the genes that showed concordance in the direction of methylation’s association with ASD between fetal sexes, were significantly enriched for canonical neurodevelopmental pathways including calcium signaling (hsa04020), glutamatergic synapse (hsa04724), and cGMP-PKG signaling (hsa04022) [Supplementary Fig. 29B]. Divergent methylation of MAPK signaling gene pathways in association with ASD in males versus females may be a byproduct of more general sex differences in the timing or rhythmicity of prenatal neuronal development, though future studies are needed to assess this hypothesis^88–90^.

Overlapping epigenetic alterations in ASD and MO-associated DMRs suggest convergent mechanisms linking maternal metabolic disruption and ASD development. Multiple maternal metabolic conditions—including diabetes, hypertension, and obesity—have been linked to increased ASD risk in offspring across epidemiological cohorts, yet the biological intermediaries of these associations have remained poorly defined^27–31,91–94^. Pre-pregnancy BMI was shown to associate with altered methylation of 27 loci in the placenta that were significantly enriched for oxidative stress-induced signaling pathway genes, and has been shown to associate with hypermethylation of the *LEP* promoter and reduced leptin expression, possibly making the placental environment more susceptible to inflammation^95,96^. Our findings fortify existing hypotheses that tie MO and ASD risk through shared genetic and epigenetic impacts on convergent biological pathways, particularly those related to inflammation^46,97,98^. For example, the shared KEGG enrichment in some ASD- and MO-associated DMR sets for pathways such as Rap1 signaling, retrograde endocannabinoid signaling, and Phospholipase D signaling highlight shared epigenetic profiles between ASD and MO concentrated in immune activation and cellular stress response. The presence of *NHIP* in all four cfDNA DMR gene lists suggests oxidative stress as an axis of potential MO-influenced ASD susceptibility^39^. While inflammation and oxidative stress have previously been proposed as mechanistic links between MO and ASD, our findings provide evidence that this connection is epigenetically encoded during prenatal development, supporting future studies into the regulatory consequences of DNA methylation changes on inflammation, reactive oxygen species levels, and synaptic development.

Crucially, our use of maternal cfDNA, which includes DNA from fetal trophoblast cells of the placenta, demonstrates the feasibility of capturing fetal epigenetic regulation in vivo using noninvasive methods^52–54^. Prior cfDNA studies in cancer, transplantation, and aging demonstrate broad utility, and we now extend this to the interrogation of a neurodevelopmental outcome^56–58,99–101^. Leveraging WGBS, we move beyond array-based coverage limitations and identify DMRs across both genic and intergenic regions, enhancing our ability to capture regulatory elements implicated in disease. These methodological advances open new avenues for investigating a range of prenatal exposures beyond MO, including maternal stress, infection, environmental toxicants, and other modifiable factors recognized as contributors to ASD and related outcomes^102^.

Moreover, the capacity to stratify cfDNA-based epigenetic signatures by fetal sex adds a critical dimension to biomarker development and mechanistic inference. Sex differences in placental development may interact with maternal exposures to yield differential risk, and our findings underscore the importance of sex-stratified analyzes in perinatal epigenetic studies^42,103,104^.

Our limitations warrant acknowledgment. Firstly, we did not adjust for estimated fetal fraction while defining our DMRs, and thus we cannot attribute differences in methylation between our cases and controls to fetal versus maternal cfDNA. This was an intentional choice, arising both from the unreliability of fetal fraction estimation for WGBS data as well as an interest in both maternal and fetal cfDNA as an aggregate source of potential etiological insight. Whether our reported ASD DMRs are driven by changes to maternal or fetal cfDNA is a question to be interrogated by future studies. A set of cfDNA DMRs where an attempt was made to correct for fetal fraction, despite unreliable quantification, has been provided for comparison [Supplementary Data 26–29]. Furthermore, cell-free DNA reflects a mixture of placental and fetal tissues, and although the ASD epigenomic signature was highly reflective of synaptic functions in the brain, it does not provide direct brain-specific resolution. Future integration with placental and fetal tissues from ASD animal models will clarify the functional consequences of observed methylation changes on brain and behavior. Additionally, we were limited in sample size due to the high volume of plasma required for cfDNA isolation. Larger studies are needed to confirm DMR reproducibility. Finally, the association of any single DMR reported in this study should be replicated in other populations, as the definition of DMRs by the passing of an empirical permutation *p*-value threshold holds the potential for Type 1 errors.

In conclusion, our work supports a model in which both inherited and environmental risk factors for ASD converge in altered DNA methylation at synaptic gene pathways in the fetal brain. The ability to detect epigenetic signatures of fetal brain in maternal blood via cfDNA provides a minimally invasive approach to discover the origins of neurodevelopmental conditions and their modulation by the intrauterine environment. This framework could be extended to interrogate other prenatal risk factors and conditions, expanding our understanding of how maternal physiology shapes fetal neurodevelopment. As methods for cfDNA profiling continue to mature, they may ultimately inform diagnostic biomarkers aimed at the rapid detection of ASD early in life, maximizing the efficacy of early interventions.

## Methods

### Sample population and diagnostic classification

All samples in this study were taken from participants in the Markers of Autism Risk in Babies – Learning Early Signs (MARBLES) prospective cohort study, which recruits women of enriched risk for ASD pregnancies in order to study etiologic factors of ASD^66^. The University of California, Davis Institutional Review Board and the State of California Committee for the Protection of Human Subjects approved the MARBLES study protocols (IRB# 225645). No data or biospecimens were collected for this study until written informed consent was obtained from the parents. All ethical regulations relevant to human research participants were followed.

The MARBLES study enrolls women who are either pregnant or planning an additional pregnancy and already have a child diagnosed with ASD. Women whose children would be the half-siblings of a child with ASD are also enrolled. Additional inclusion criteria are as follows: the mother is 18 years of age or older; the mother speaks, reads, and understands English proficiently enough to complete the protocol and the younger sibling will be taught to speak English; and the mother lives within 2.5 h of the Davis/Sacramento region at time of enrollment. Recruitment into the MARBLES study is performed primarily via the California Department of Developmental Services (DDS). ASD diagnosis in the proband (older affected sibling or half-sibling) is confirmed during the enrollment process. Enrolled mothers participate in phone interviews and complete questionnaires both pre-pregnancy and during pregnancy, collecting demographic, diet, and medical information, including maternal pre-pregnancy BMI. After the child is born, ASD symptomatology is assessed at 6, 12, 24, and 36 months of age by trained psychologists and study staff applying age-appropriate instruments. Participant children were not screened for any specific chromosomal abnormalities or copy number variants known to be associated with ASD, and were not excluded from consideration in this study based on the presence or absence of ASD-associated genetic variants. The final neurodevelopmental outcome of the child is determined by an outcome algorithm based on ADOS score and MSEL score results, using methods previously published^105^. Children classified as non-Typically Developing but not ASD were grouped with Typically Developing children into a “non-ASD” group for the purpose of DMR identification.

For this analysis, MARBLES participants were chosen based on the availability of blood plasma samples of sufficient volume (1$\ge \,$ ml) for cfDNA extraction. Actual sample volumes in the 0.6–1 ml range were able to yield sufficient cfDNA for 93.7% (59/63) of samples. Plasma samples were derived from 2 ml whole blood collected in EDTA storage tubes. Whole blood samples were collected prior to delivery and stored on ice for no more than 4 h prior to centrifugation. Samples were centrifuged at 1000 × *g* for 15 min to separate out the plasma, which was subsequently aliquoted in 0.5 ml amounts into fresh cryovials. Plasma aliquots were stored at −80 °C. Two frozen plasma aliquots from selected participants were thawed and pooled for cfDNA isolation.

### Cell-free DNA extraction and whole-genome bisulfite sequencing (WGBS)

CfDNA was extracted from the supplied plasma aliquots with the QIAamp Circulating Nucleic Acids Kit (QIAGEN, 55114) by following recommended procedures and quantified with the Qubit DNA Assay Kit (ThermoFisher Scientific, Q32851). All purified cfDNA samples were also analyzed with the Bioanalyzer High Sensitivity DNA Kit (Agilent, 5067-4626) to verify sample concentrations and validate that samples contained only the small nucleic acids typical of cfDNA. Any samples containing nucleic acids of ≥200 bp length were subjected to a size-selective clean-up with SPRIselect (Beckman Coulter, B23317) beads.

CfDNA was bisulfite-converted using the EZ-DNA Methylation Direct Kit (Zymo Research, D5020) according to manufacturer recommendations. No fragmentation was performed, as cfDNA are naturally of a short length amenable to library preparation^106^. Whole genome bisulfite sequencing libraries were then prepared from the bisulfite-converted cfDNA using the xGen Methylation-Sequencing DNA Library Preparation Kit (IDT, 10009860) with UDI Primer Pairs (IDT, 10005922) and an 11-cycle indexing PCR. Libraries were assessed for uniform size with Bioanalyzer High Sensitivity DNA Kit and pooled to 10 nM concentration with a 10% PhiX spike-in. Pooled libraries were sequenced on 4 lanes with 150 bp paired end reads of Illumina NovaSeq 6000 S4 (Illumina, 20012850) by the DNA Technologies Core at UC Davis, producing an average 209M sequences per sample and an average of 9.6X CpG coverage.

### WGBS alignment and quality control

Raw sequencing files were preprocessed, aligned to hg38, and used to generate cytosine reports with Epigenerator^107^. In short, Epigenerator provides a user-friendly means of generating cytosine reports from fastq files by applying the FASTQ_Me and CpG_Me workflows in tandem^108,109^. Merged reads were trimmed of adaptors, corrected for potential methylation bias at the 5′ and 3′ ends, and aligned to hg38. The aligned reads were then deduplicated, and CpG methylation was extracted to generate cytosine reports for further analysis. FastQC and MultiQC were also applied to check the sequencing quality of all samples^110,111^. Fetal fraction was estimated by calculating ChrY:ChrX reads and via SeqFF, although these estimates should be interpreted cautiously due to the nature of WGBS libraries defying many of the assumptions of conventional fetal fraction estimation^112–115^ [Supplementary Fig. 30]. While the SeqFF-derived estimate of fetal fraction varied substantially from expected values for third-trimester plasma, it did demonstrate a positive linear correlation with ChrY:ChrX read ratios, thus indicating a rough relationship to true fetal fraction in aggregate, even if individual sample estimates are suspect [Supplementary Fig. 31].

### Detection of DMRs

DMR calling and some downstream enrichment analyzes were performed with DMRichR v1.7.1^116^. DMRichR combines dmrseq and bsseq tools to enable the inference of DMRs based on user-supplied variables of interest. The dmrseq algorithm pools the differences in CpG methylation between the case (ASD) and control (non-ASD) groups and performs smoothing to assemble background regions. Possible regions were defined as sections of the genome that had a minimum of 5 CpGs with a maximum gap of 1000 bp, with each CpG being required to have at least 1X coverage in 75% of our sample group. A statistic for each possible region was then estimated through a generalized least squares regression, and permutation tests were used to identify regions with significantly differential methylation between case and control groups. No curated regions of the genome were masked in the process of calling DMRs, enabling DMRs to be called from any regions in the genome meeting the specified coverage standards. This includes regions of known high variance. A methylation difference of 5% was used as the minimum threshold for the magnitude of difference between regions to be considered significant as a DMR.

DMRs were identified for both ASD and MO in fetal sex-stratified groups, as prior investigations have shown sex-specific DNA methylation signatures in perinatal tissues^42^. A child was defined as having ASD if their ADOS overall total score at 36 months surpassed the ADOS cutoff and the child met *DSM-5* criteria for ASD. All children not meeting these criteria, including those with developmental delays on the MSEL, were defined as non-ASD controls. A mother was defined as having MO if her reported pre-pregnancy BMI in the intake questionnaire was ≥30 kg/m^2^, with all mothers of lesser BMI, including those meeting overweight criteria, classified as non-obese Control. Four distinct sets of cfDNA DMRs were identified: Male ASD DMRs (*N* = 33, 14 ASD and 19 non-ASD control), Female ASD DMRs (*N* = 18, 10 ASD and 8 non-ASD control), Male MO DMRs (*N* = 33, 9 MO and 24 non-obese control), and Female MO DMRs (*N* = 18, 9 MO and 9 non-obese control) [Supplementary Data 25]. The two ASD DMR sets were adjusted for MO as a significant covariate, as MO is a known upstream risk factor for ASD development, but the reverse causality was not expected, so there was no adjustment for ASD in MO versus Control^27^. All DMRs were called with the default variable specifications, with the exception that the maximum number of permutations tested was increased to 33 for the males and 18 for the females to improve statistical rigor. Significant DMRs were defined as those with a permutation *p*-value of < 0.05. DMRs were annotated with their nearest known gene via the application of “one closest” rule of the Genomic Regions Enrichment of Annotations Tool (GREAT)^117^. These DMR gene name lists were used in future enrichment analyzes.

Despite concerns regarding the accuracy of SeqFF estimated fetal fraction, this metric was included as an adjusted for covariate in a separate group of DMRichR runs to identify ASD and MO DMRs from cfDNA. Region inclusion parameters for this batch of DMRs were identical to previous batches, and a minimum threshold of 5% difference between compared groups was still used as a cutoff to call a given region a DMR. In both males and females, the ASD and MO DMRs identified after adjustment for SeqFF as a covariate did not exhibit sizable deviation from the previously identified DMRs [Supplementary Data 26–29]. All SeqFF-adjusted ASD and MO DMRs exhibited significant overlap with the previously identified DMRs as quantified both by gene name overlap and genomic region overlap [Supplementary Figs. 32–37]. Regardless of whether SeqFF is adjusted for during DMR identification, the resulting ASD DMR genes are significantly enriched for KEGG pathways involved in synapse development and cell signaling in both males and females [Supplementary Fig. 38]. The inclusion of SeqFF as an explicit adjusted-out covariate also has no impact on whether the resulting cfDNA ASD DMRs are significantly enriched for placenta and cortex ASD DMRs [Supplementary Fig. 39].

To visualize the correlations between the identified DMRs and other covariates of the sample set, regression analyzes were conducted for every covariate ~ DMR combination, with significance of a given association reported as a *p*-value from a *t*-test of the regression coefficient [Supplementary Figs. 40–43].

For the ASD DMRs identified from previously published placenta and cortex samples, data were acquired in the form of cytosine reports and subjected to sex-stratified DMR identification under the same parameters as previously described. Placenta and cortex DMRs were tested for overlap with age-predictive CpGs from previously published models using the GRanges package [Supplementary Data 30]^118,119^. Average CpG coverage over placenta ASD DMRs was compared between ASD and Control groups to investigate whether copy number variants may be driving differences in methylation, although no significant associations (*q* < 0.05) were discovered [Supplementary Data 31 and 32]. Fisher’s exact tests of DMRs with nominally significant (*p*-value < 0.05) associations with CpG coverage were performed to identify potential overrepresentation of chromosomes [Supplementary Data 33 and 34].

### Global methylation averages between male and female samples

Per-sample global averaged methylation values per CpG were calculated for all cfDNA samples with the “globalStats” function from the DMRichR package. Samples were grouped by fetal sex and plotted with Prism v10.3.0. Significance of the difference between male and female groups was calculated by an independent two-tailed *t*-test under the assumption of a normal distribution.

### DMR enrichment analysis

Enrichment of DMR sets for genic and CpG annotations was performed with the “DMRichGenic” and “DMRichCpG” functions from the DMRichR package, respectively. In both cases, a Fisher’s exact test was used to determine significant overrepresentation (*q* < 0.05) of a given genic or CpG annotation in the DMR set, with the background universe set as possible regions assessed for differential methylation within a given sample set. The resulting enrichments were then plotted with “DMRichPlot” from the DMRichR package.

Enrichment of DMR sets for gene ontology was performed with the “submitGreatJob” function from the rGREAT package v4.0.4 using the one closest rule to assign genes to regions and slimmed with the “slimGO” function from DMRichR. A binomial distribution is assumed, and the input region set was tested for overrepresentation of any ontologies relative to background regions. GREAT enrichments were plotted with “ggplot” from ggplot2.

KEGG pathway enrichments were determined with the “enrichKEGG” function from the clusterProfiler package v4.16.0. A Fisher’s exact test was used to test for overrepresentation, with all KEGG-annotated genes as the background universe. A Benjamini–Hochberg false discovery rate correction was applied, and a *q*-value threshold of 0.05 determined significance.

### DMR overlap analysis

All testing of the significance of region-level overlaps between sets of DMRs was performed using the “permTest” function of the regioneR package v1.32.0. Both numOverlaps and meanDistance are used for the evaluation of the number of region overlaps and the average distance between those overlaps, respectively. Regions were randomly reassigned across the entirety of the genome (hg38) and tested over 10,000 permutations. The results were then plotted with the “plot” function from the R base package v. 4.5.0.

### SFARI enrichment analysis

All SFARI Gene database enrichments tests were performed with tools from the GeneOverlap package v1.44.0. First, the “newGeneOverlap” function was applied to all DMR gene lists, with the genome size defined as the length of the gene list containing all possible background regions from our sequencing data. Next, the “testGeneOverlap” function performed Fisher’s exact tests to determine whether a given input DMR gene list coincided with the SFARI gene list more frequently than expected by chance. The SFARI Gene list used for these enrichment tests was the 2023 Q4 release. Plotting of SFARI Gene intersections with DMR gene lists was performed with the “plot” function from R base package v4.5.0.

### Developmentally differentially methylated genes overlap analysis

A list of 28,718 differentially methylated positions across fetal brain development was acquired from a pre-existing study of fetal epigenetic dynamics^70^. The genic annotation of these positions was extracted, providing a list of 7693 genes with positions of dynamic methylation throughout pregnancy. The “newGeneOverlap” and “testGeneOverlap” functions from the Gene Overlap package v1.44.0 were then used to perform Fisher’s exact tests on the enrichment of these 7693 genes with the male and female cfDNA ASD DMR gene lists. For this test, the background universe for comparison was the intersecting genes between the Illumina Infinium HumanMethylation 450 BeadChip (Illumina WG-314) and the background regions from the appropriate cfDNA DMR set.

### Chromatin state enrichment analysis

Chromatin state enrichment analysis was performed using the ChromHMM 15-state model for hg38 from the Roadmap Epigenomics Project via the LOLA framework^71,120^. Significant cfDNA DMRs and matched background regions were submitted with the “runLOLA” function of the LOLA R package v1.38.0 with a reference database of ChromHMM segmentations across multiple tissue and cell types. Chromatin state enrichment *p*-values were computed using Fisher’s exact test. Chromatin states were annotated with human-readable labels, and infinite values were replaced with the highest finite odds ratio to enable plotting. Heatmaps of enriched states across tissues were generated with the “heatmap.2” function of the gplots R package v3.2.0.

### Statistics and reproducibility

Details of the statistical methodology used for DMR identification have been provided earlier in this section. A total of 51 cfDNA samples from 51 unique pregnancies were used in the identification of cfDNA DMRs. For the identification of male ASD cfDNA DMRs, 14 replicates of pregnancies of children later diagnosed with ASD and 19 replicates of pregnancies of children not later diagnosed with ASD were employed. For the identification of female ASD cfDNA DMRs, 10 replicates of pregnancies of children later diagnosed with ASD and 8 replicates of pregnancies of children not later diagnosed with ASD were employed. In the identification of male MO DMRs, 9 replicates of MO and 24 replicates of non-MO mothers were used. In the case of female MO DMRs, 9 replicates of the MO condition and 9 replicates of the non-MO condition were assessed. Further details on the statistical analyzes employed throughout this manuscript are documented within the relevant Methods subsection.

### Reporting summary

Further information on research design is available in the Nature Portfolio Reporting Summary linked to this article.

## Supplementary information

Supplementary Information

Description of Additional Supplementary Materials

Supplementary Data 1-37

Reporting Summary

Transparent Peer Review file

## Data Availability

The sequencing data generated by this study from all subjects that have provided consent to share genomic data (*n* = 48) have been deposited in the Gene Expression Omnibus (GEO) database under accession code GSE307129. Previously published WGBS data for placenta and cortex can be accessed in the GEO database under accession codes GSE178206 (placenta) and GSE119981 (cortex). All data supporting the findings of this study are available within the paper and its Supplementary Information. See Supplementary Data for more information on the source data for all figures and tables shown in this manuscript. More specifically, source data for the primary figures can be found as described: Fig. 1 - Supplementary Data, Fig. 2A – Supplementary Data 5, 6, and 7, Fig. 2B – Supplementary Data 7, Fig. 2C – Supplementary Data 9 and 10, Fig. 2D – Supplementary Data 5 and 6, Fig. 2E – Supplementary Data 5 and 6, Fig. 3A – Supplementary Data 15 and 16, Fig. 3B – Supplementary Data 17 and 18, Fig. 4A – Supplementary Data 5, 6, 15, 16, 17, 18, 19, and 21, Fig. 4B – Supplementary Data 5, 6, 15, 16, 17, and 18, Fig. 4C – Supplementary Data 20, Fig. 5A – Supplementary Data 22 and 23. Figure 5B – Supplementary Data 9, 10, 35 and 36, Fig. 6A – Supplementary Data 5, 6, 22, and 23. Figure 6B – Supplementary Data 5, 6, 22, and 23, Fig. 6C – Supplementary Data 37. All other data are available from the corresponding author upon reasonable request.

## Code availability

All original code has been deposited on GitHub^121^
