# Supplementary material for: Prenatal cell-free DNA methylome detects association with autism and maternal obesity
Source: Commun Biol. 2026 Jun 22;9:1319. doi: 10.1038/s42003-026-10500-9 (PMC13650157; doi:10.1038/s42003-026-10500-9)
Supplement: Supplementary file 1 [file 42003_2026_10500_MOESM1_ESM.pdf]

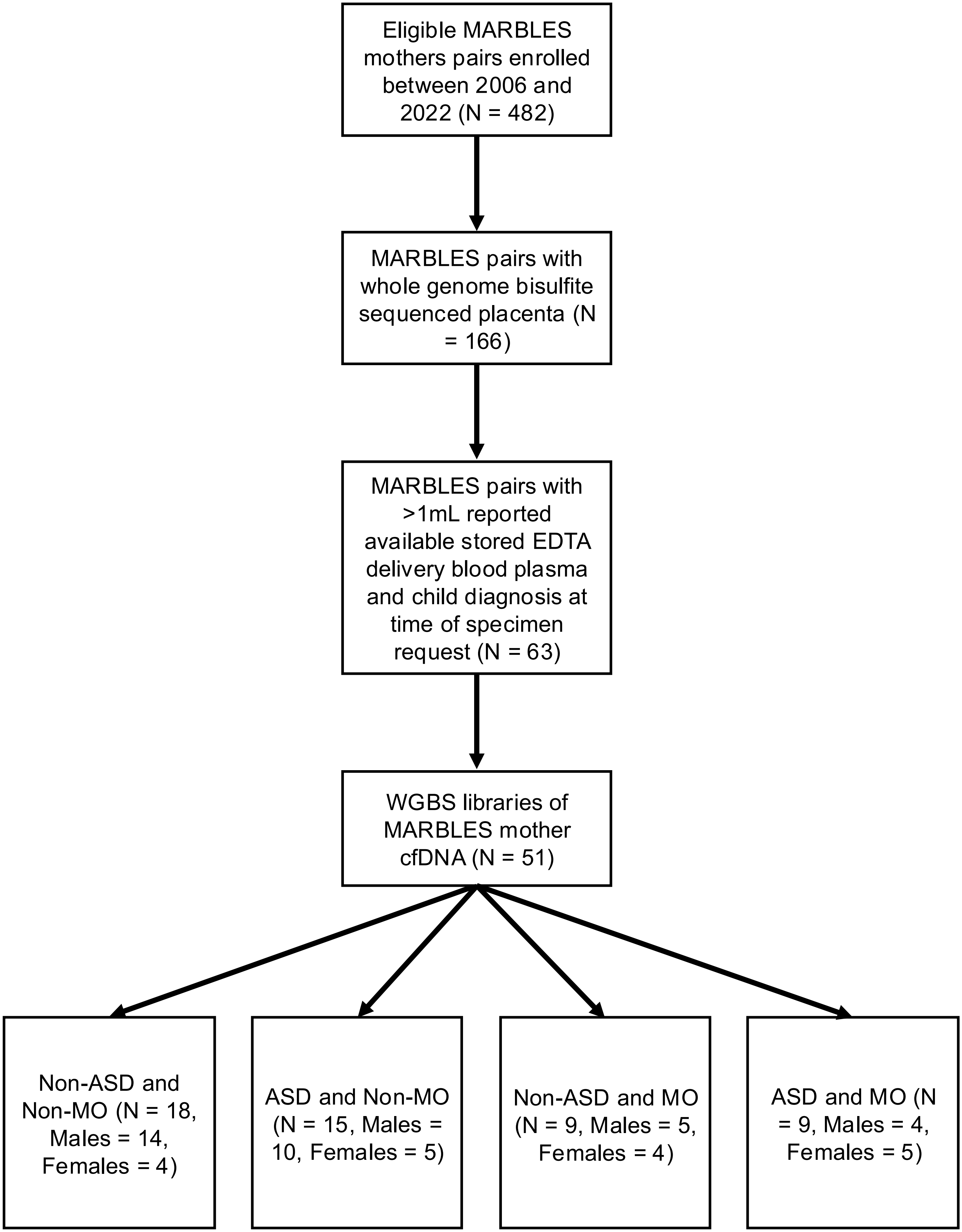

Supplementary Figure 1: Flowchart describing sample selection criteria and sample library covariates for the MARBLES samples used in this study.

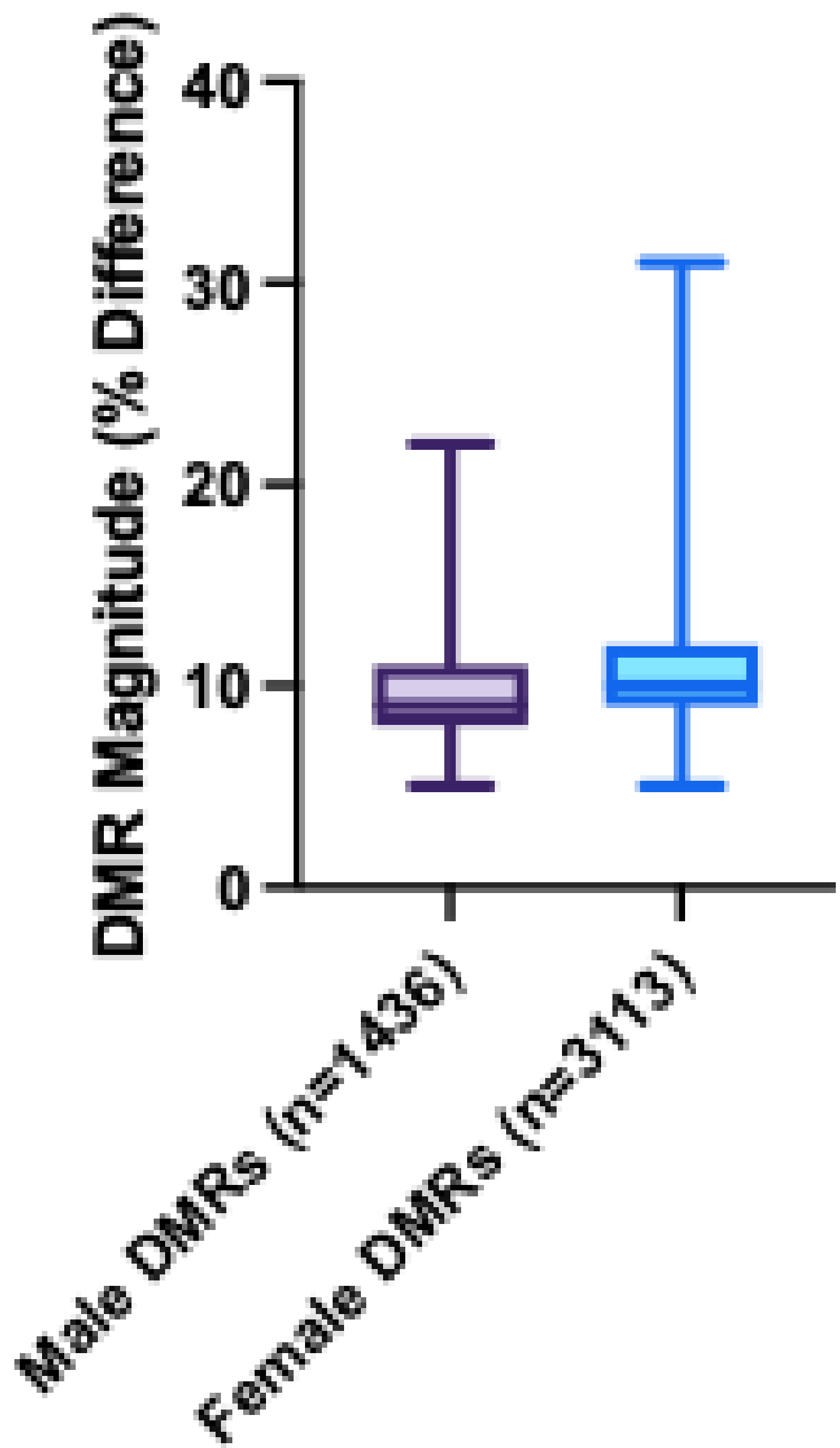

Supplementary Figure 2: Boxplot displaying the difference in DMR magnitude between male pregnancy ASD cfDNA DMRs (n = 1,436) and female pregnancy ASD cfDNA DMRs (n = 3,113). The female DMRs are significantly larger than the male DMRs (Welch's t-test, $p < 0.0001$) by a factor of +1.345% on average.

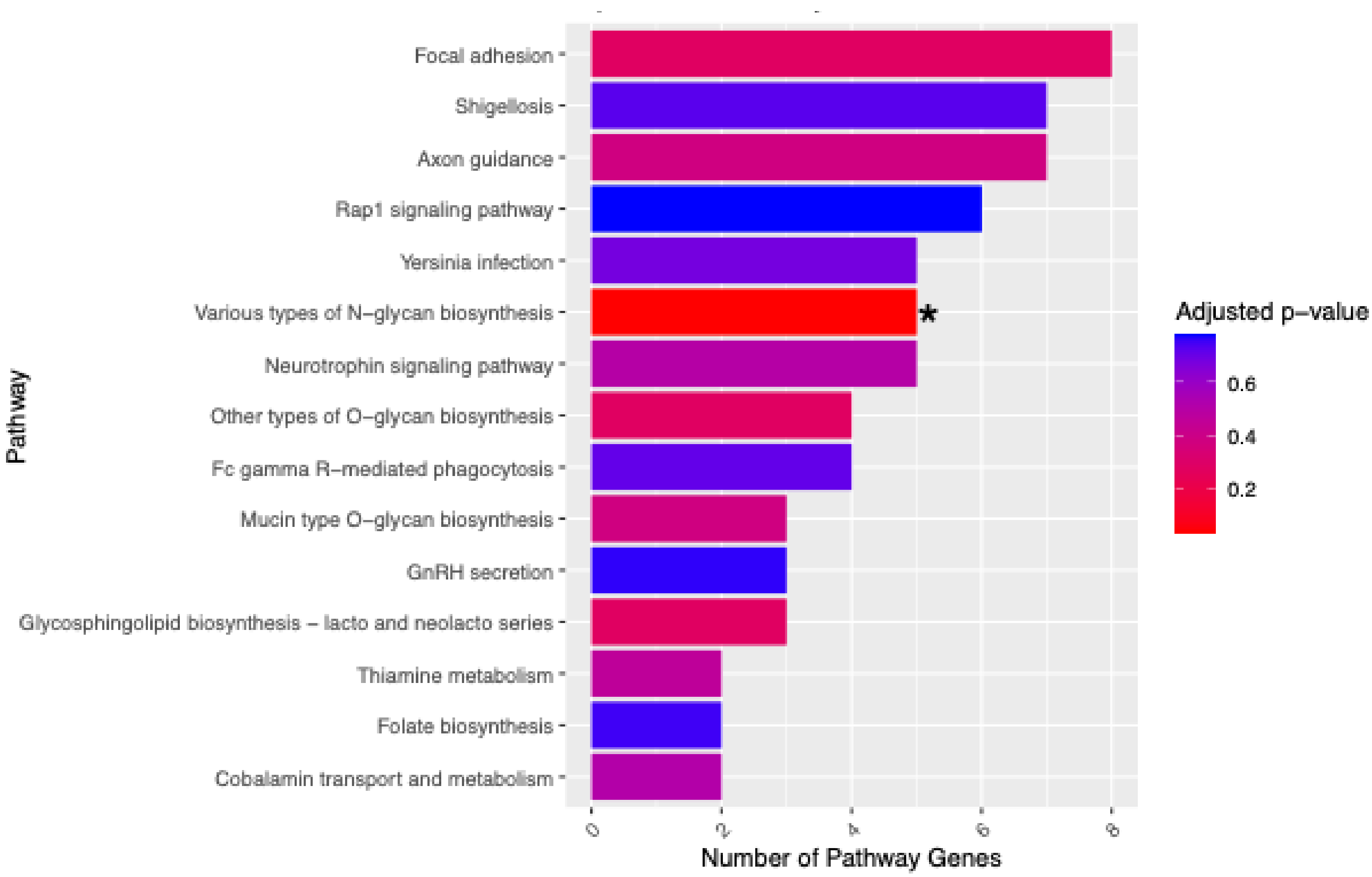

Supplementary Figure 3: The top 15 most significant KEGG pathway enrichments (by adjusted p-value) of the gene list from the cfDNA sex-combined ASD DMRs. The only significantly enriched pathway (n = 5 pathway genes) was “Various types of N-glycan biosynthesis” (hsa:00510).

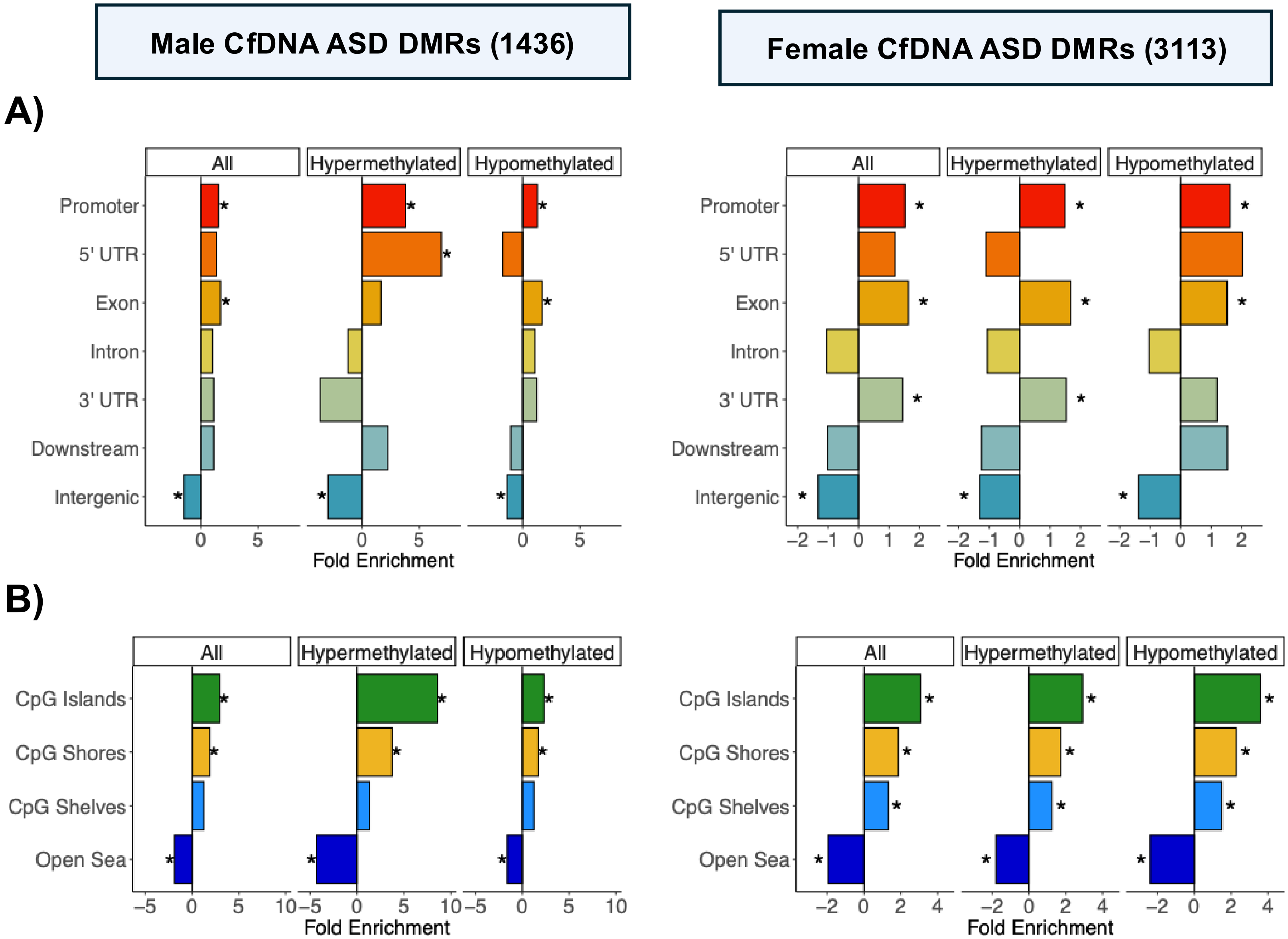

Supplementary Figure 4: Enrichment analyses of male (n = 1,436) and female (n = 3,113) cfDNA ASD DMRs (permutation p-value < 0.05) for both **A)** genic annotations and **B)** CpG annotations as performed and plotted by DMRichR. DMRs were compared to background regions for each comparison, with significance determined by Fisher's test with FDR correction (* = q-value < 0.05).

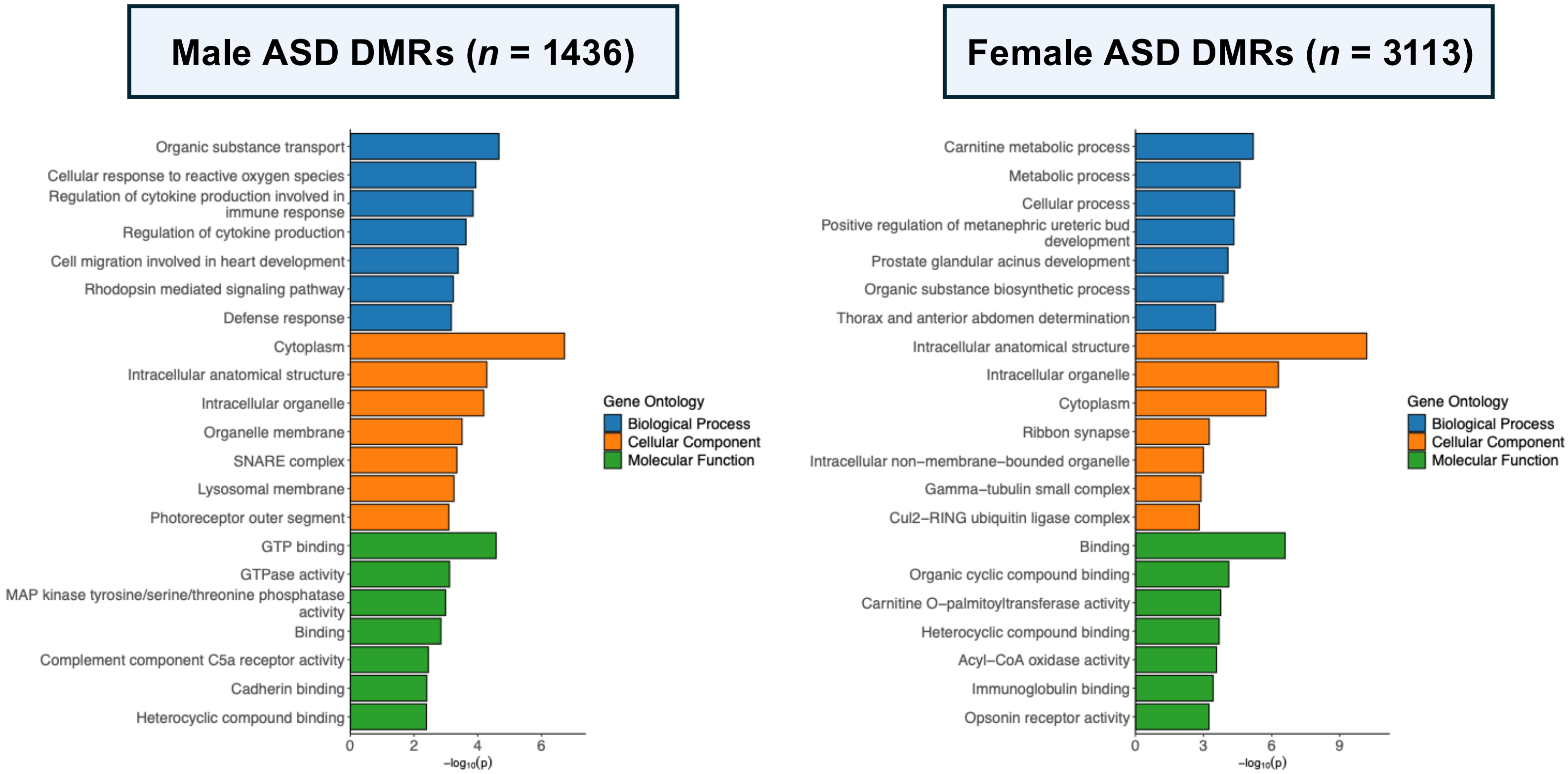

Supplementary Figure 5: The results of Gene ontology (GO) term enrichment analyses for male (n = 1,436) and female (n = 3,113) cfDNA ASD DMR gene lists, as performed by GREAT. The top seven most significantly enriched terms for each of the three gene ontologies are shown.

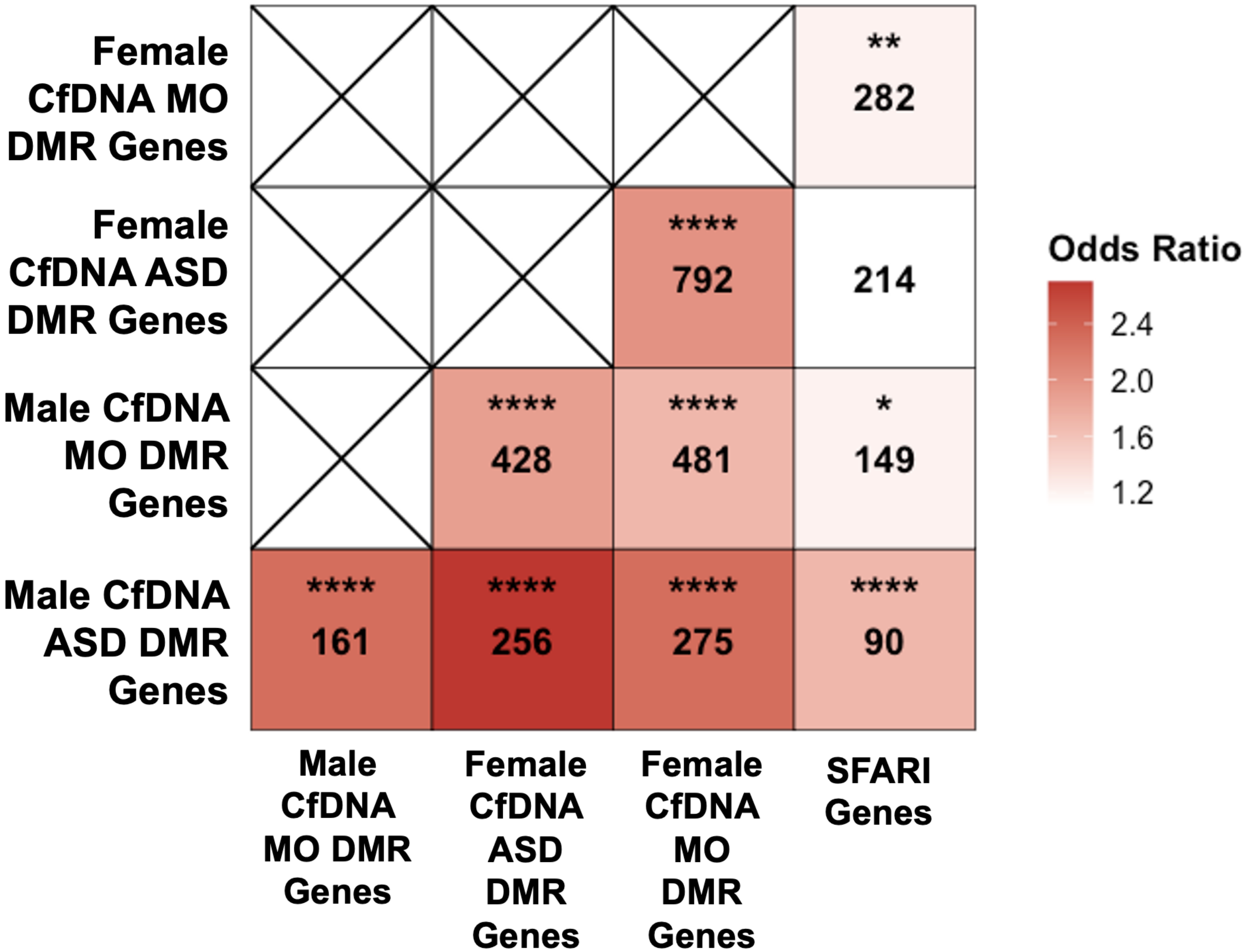

Supplementary Figure 6: Heatmap displaying the significance of gene-level overlap between all cfDNA DMRs with SeqFF fetal fraction adjustment and the SFARI Gene list. Significance determined by Fisher's Exact Test. The numbers shown on each tile correspond to the number of genes overlapping the two gene lists in question. Asterisks denote p-value as follows: * $p < 0.05$, ** $p < 0.01$, **** $p < 0.0001$

A)

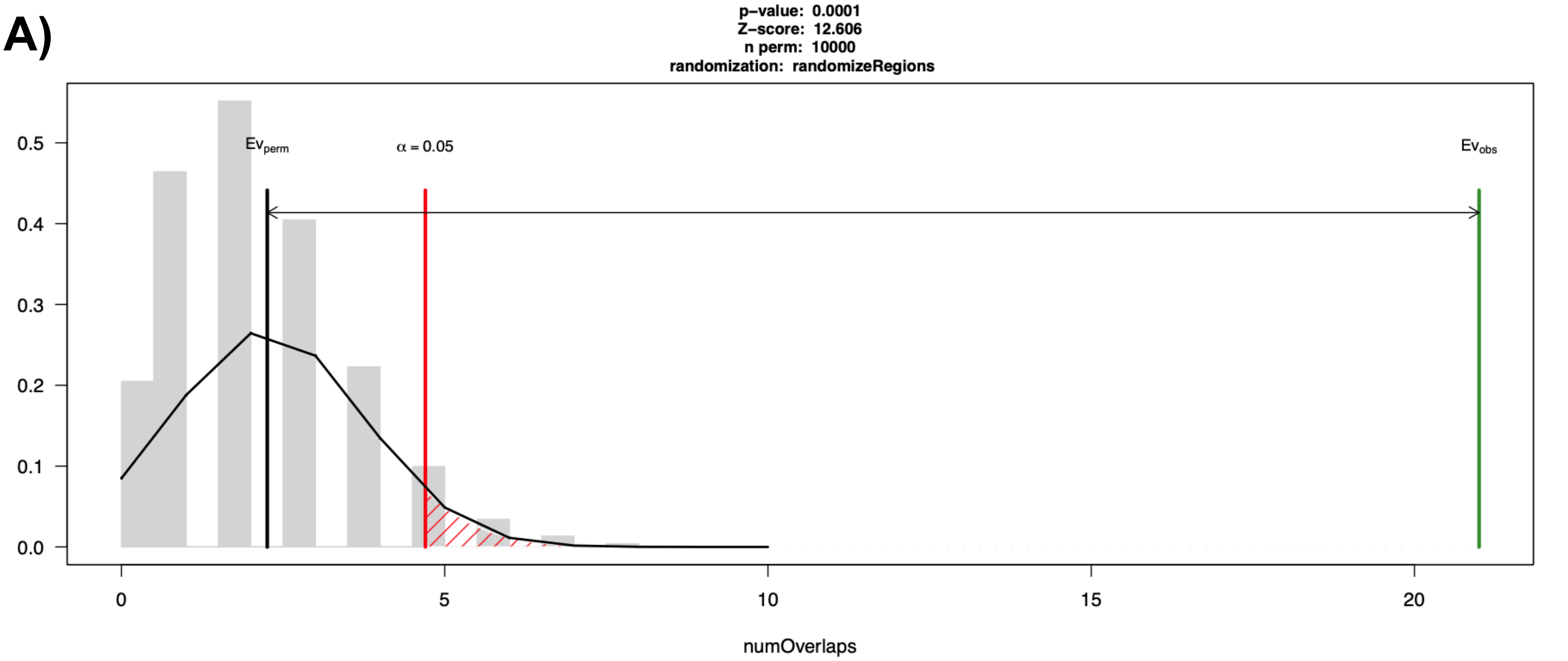

B)

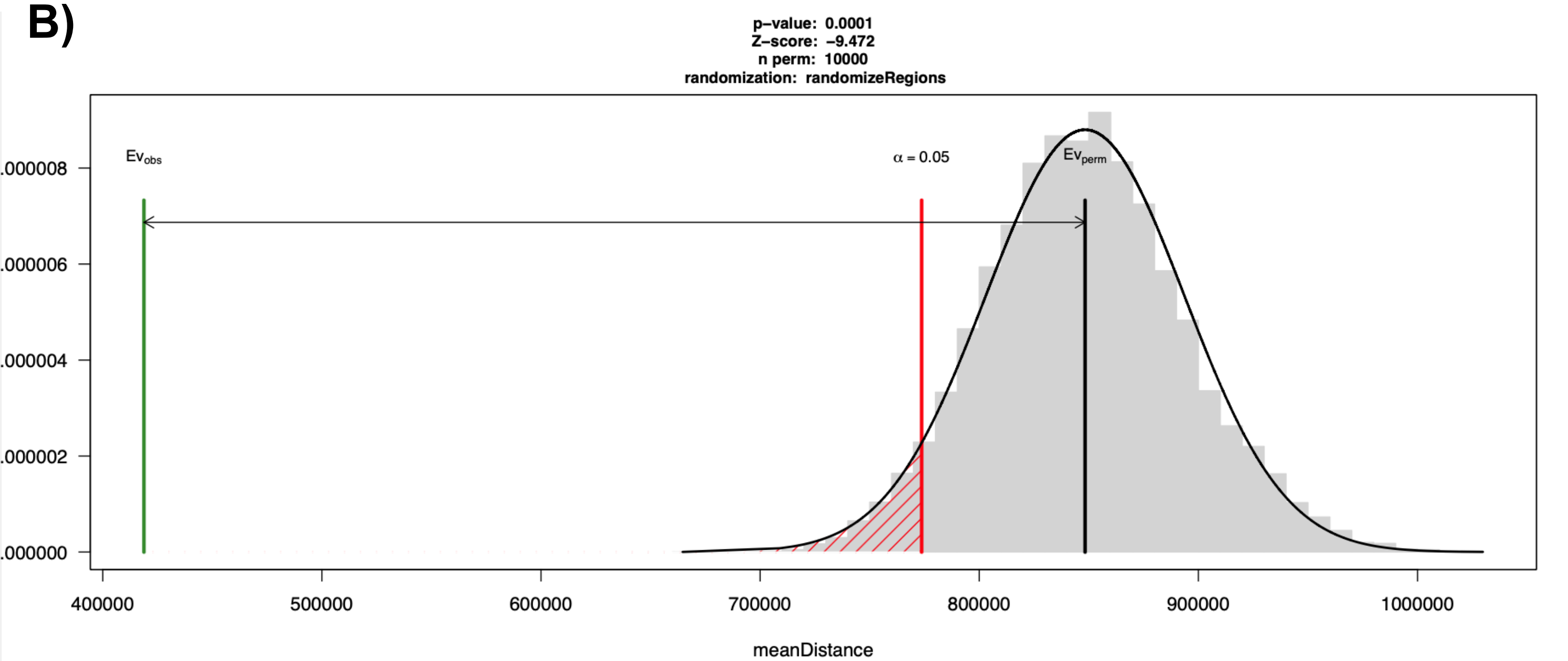

Supplementary Figure 7: Overlap analysis of the male and female cfDNA DMRs with the *regioneR* package. Analysis performed over 10,000 permutations, with the average number of **A)** overlaps between region sets and **B)** mean distance between region sets compared to the actual data, with significance calculated via permutation test.

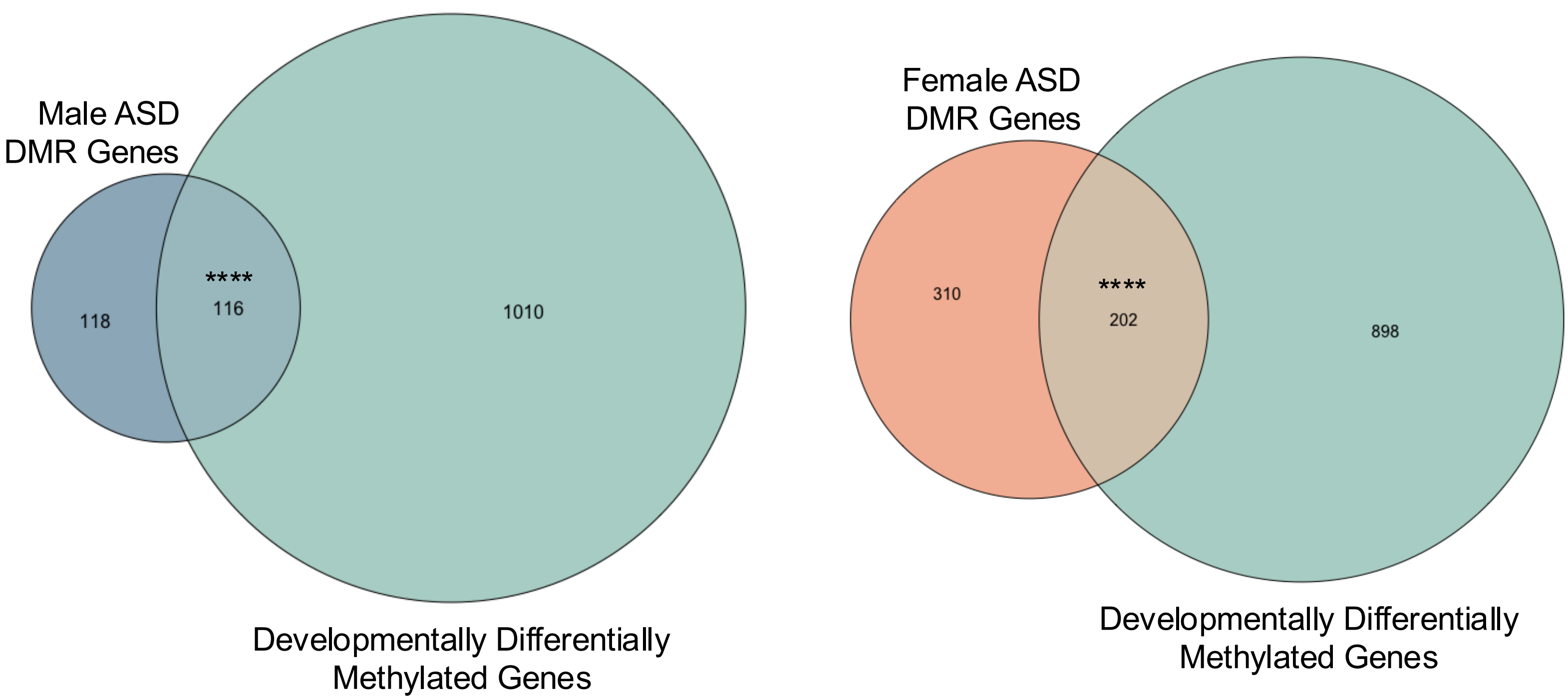

Supplementary Figure 8: Venn diagrams of male (n = 1,436) and female (n = 3,113) cfDNA ASD DMR gene lists's overlap with developmentally differentially methylated genes (DDMGs) as identified by the dDMPs in Spiers et al. 2015. Only genes appearing in the possible background of the DMRs and DDMGs included in analysis. Both overlaps determined significant compared to conserved background region overlap via Fisher's exact test (p = 2.6×10^{-13} for males and p = 2.2×10^{-10} for females).

A)

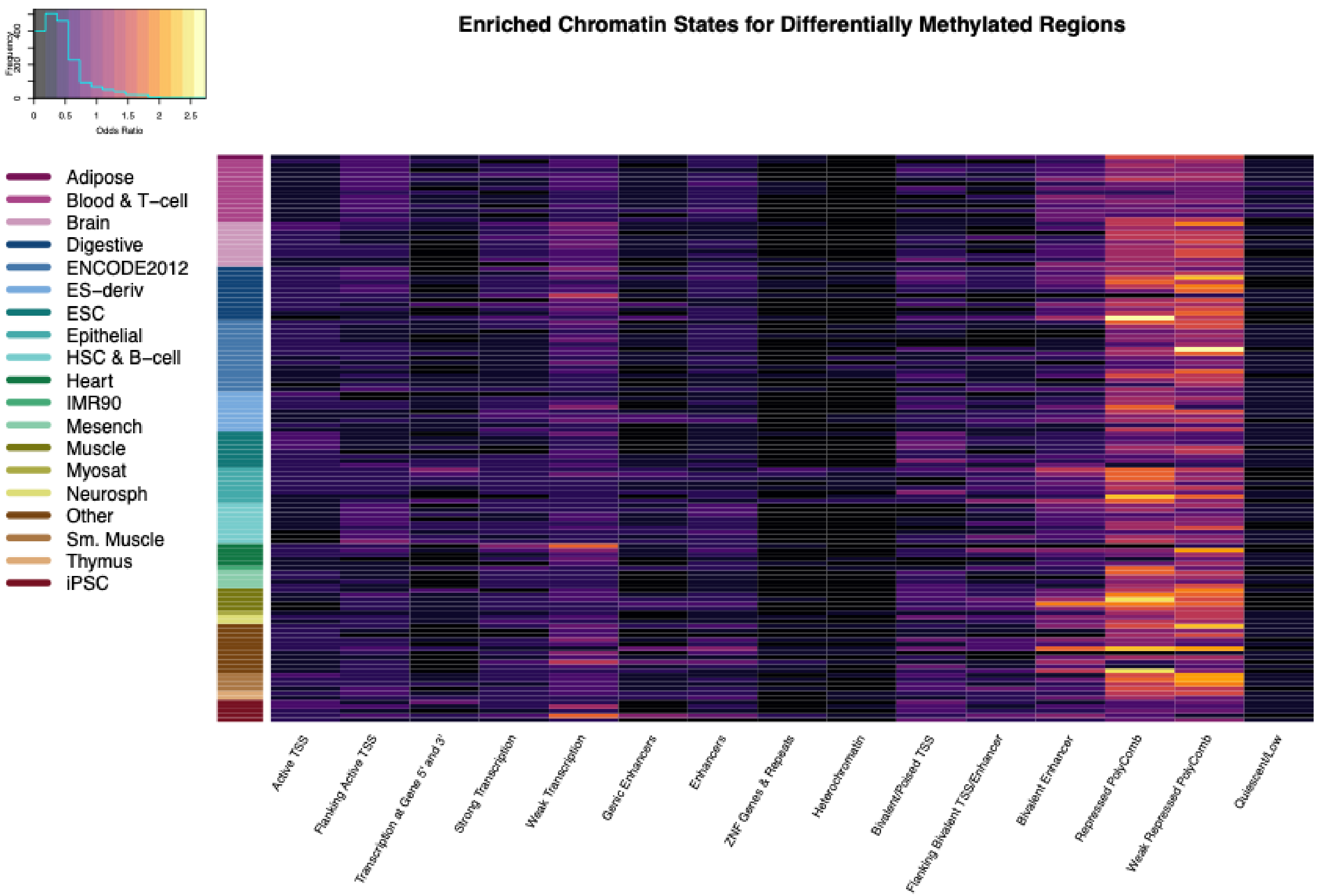

B)

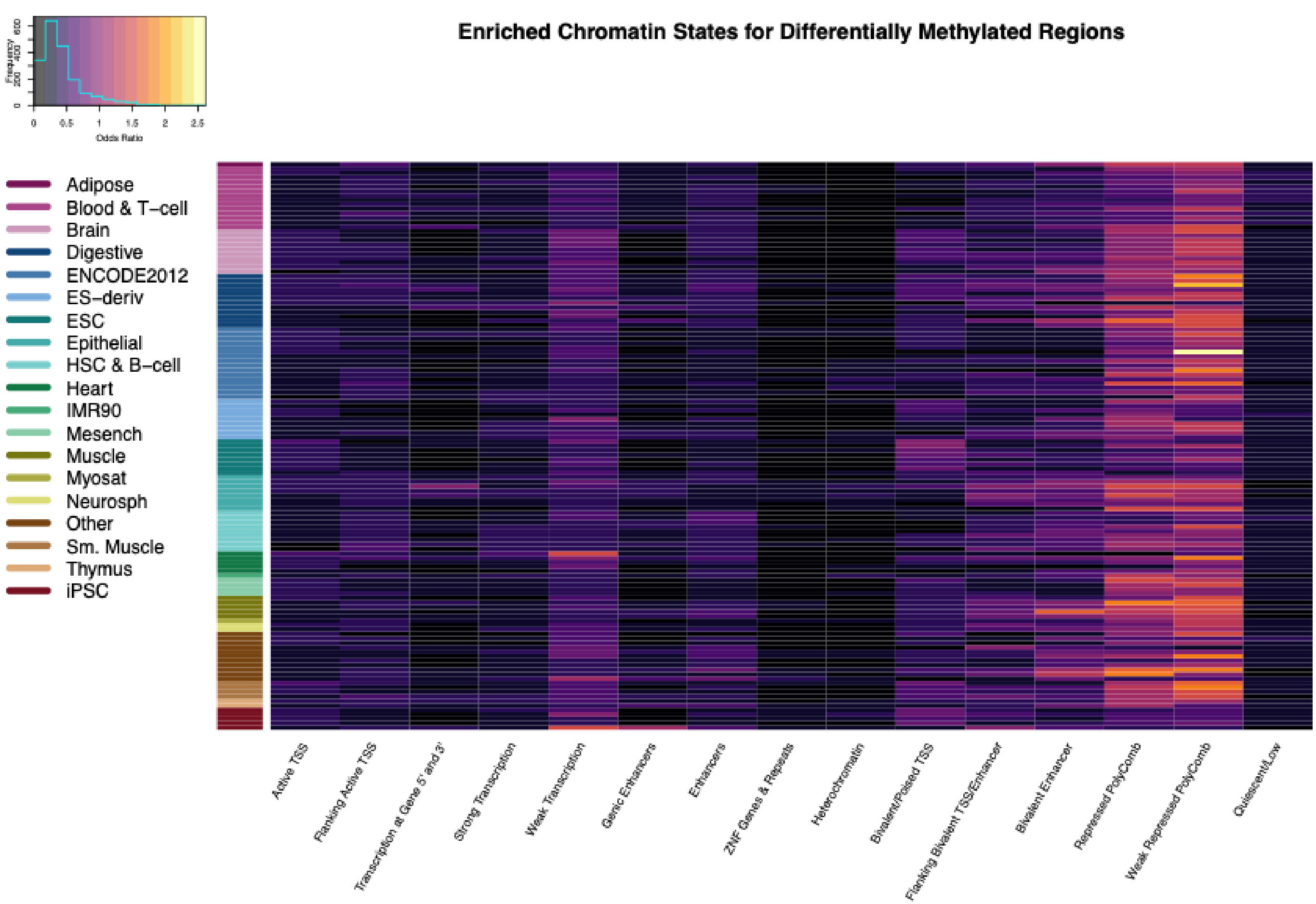

Supplementary Figure 9: The results of region epigenome enrichment analysis of **A)** male and **B)** female ASD cfDNA DMRs using the ChromHMM Epigenomics Roadmap as a reference. The intensity of the heatmap's tiles correspond to the magnitude of a given enrichment, as quantified by odds ratio.

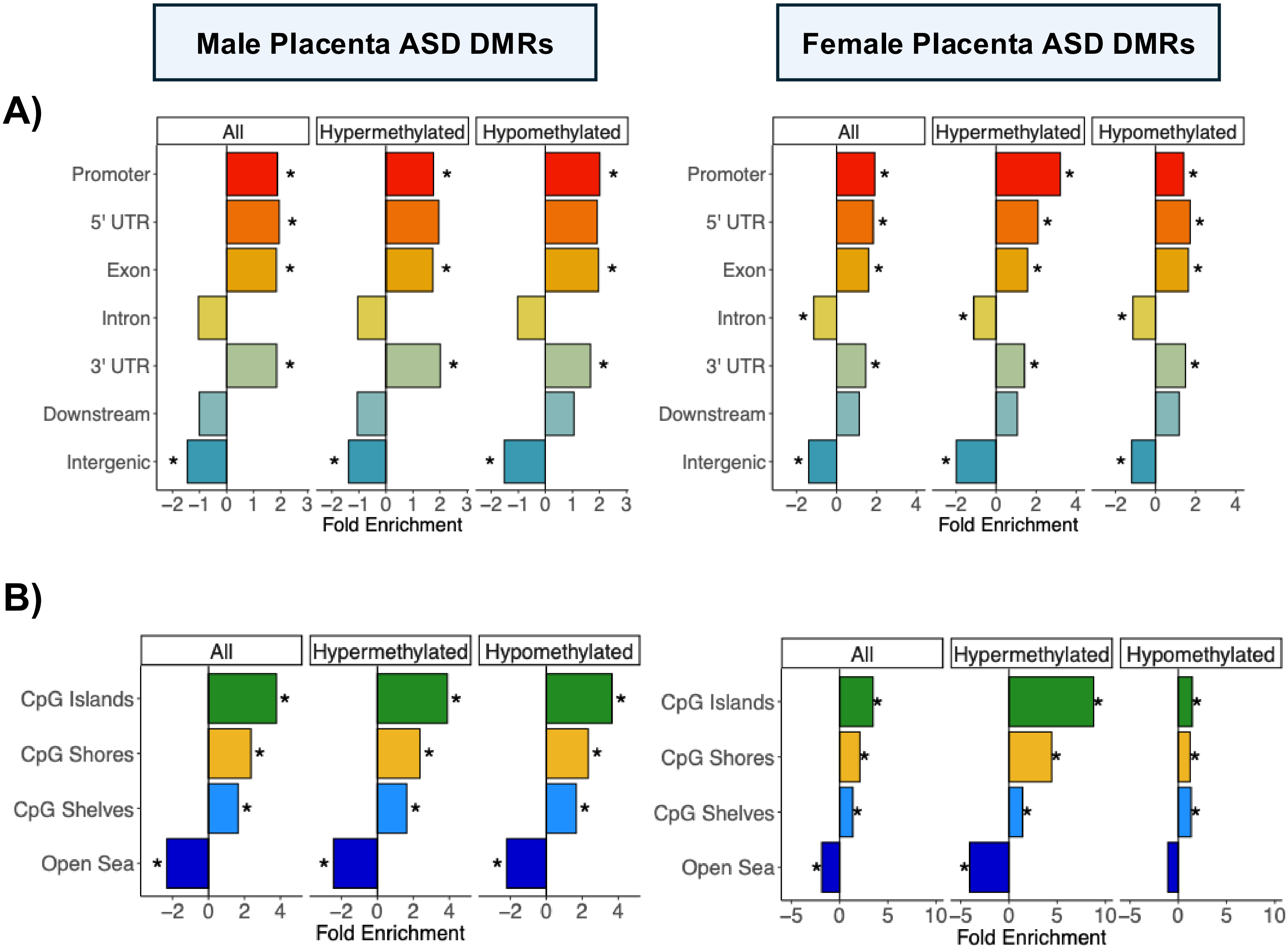

Supplementary Figure 10: Enrichment analyses of male (n = 2,678) and female (n = 8,447) placenta ASD DMRs (permutation p-value < 0.05) for both **A)** genic annotations and **B)** CpG annotations as performed and plotted by DMRichR. DMRs were compared to background regions for each comparison, with significance determined by Fisher's test with FDR correction (* = q-value < 0.05).

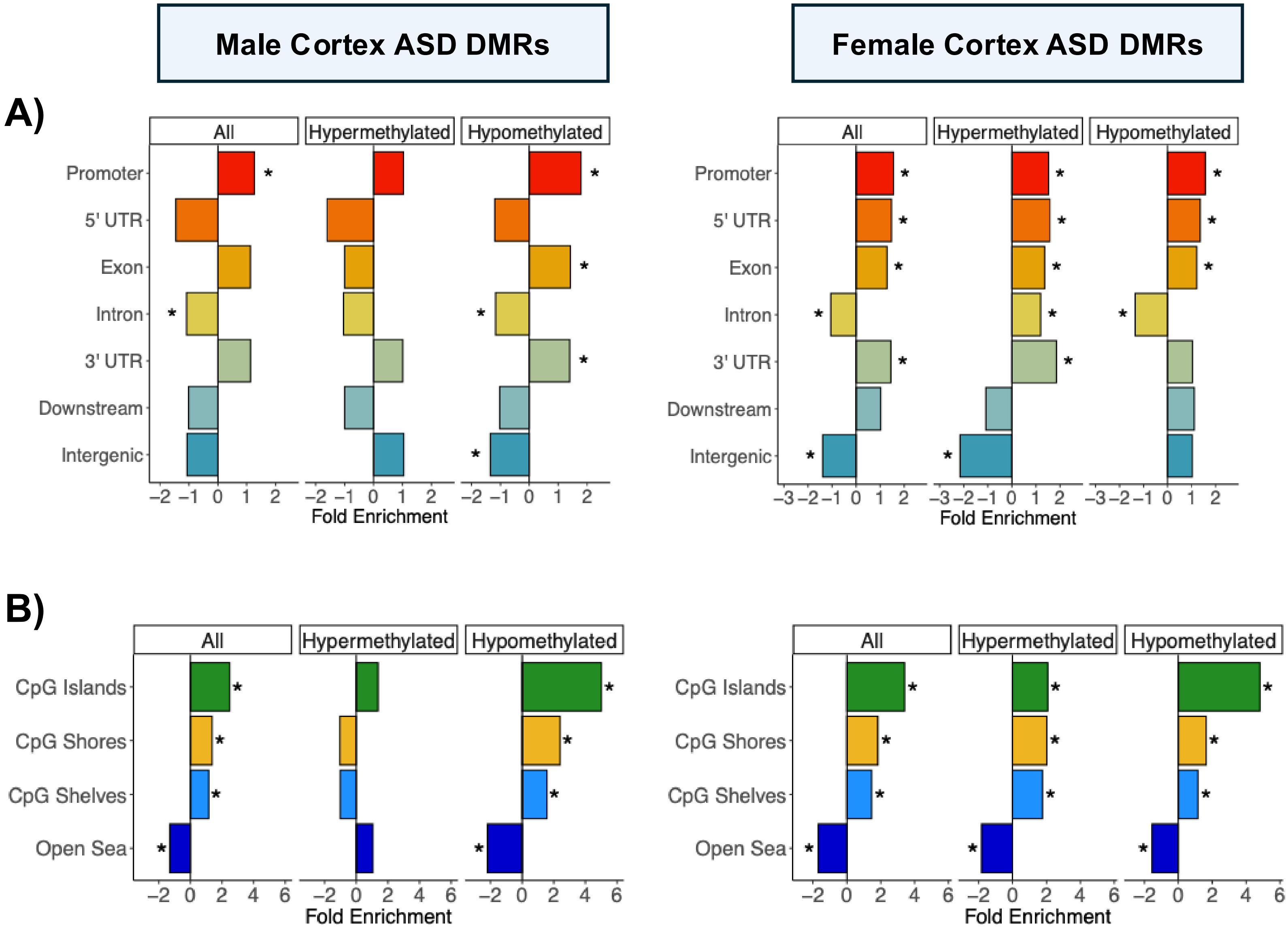

Supplementary Figure 11: Enrichment analyses of male (n = 5,191) and female (n = 16,644) Cortex ASD DMRs (permutation p-value <0.05) for both **A)** genic annotations and **B)** CpG annotations as performed and plotted by DMRichR. DMRs were compared to background regions for each comparison, with significance determined by Fisher's test with FDR correction (* = q-value < 0.05).

A)

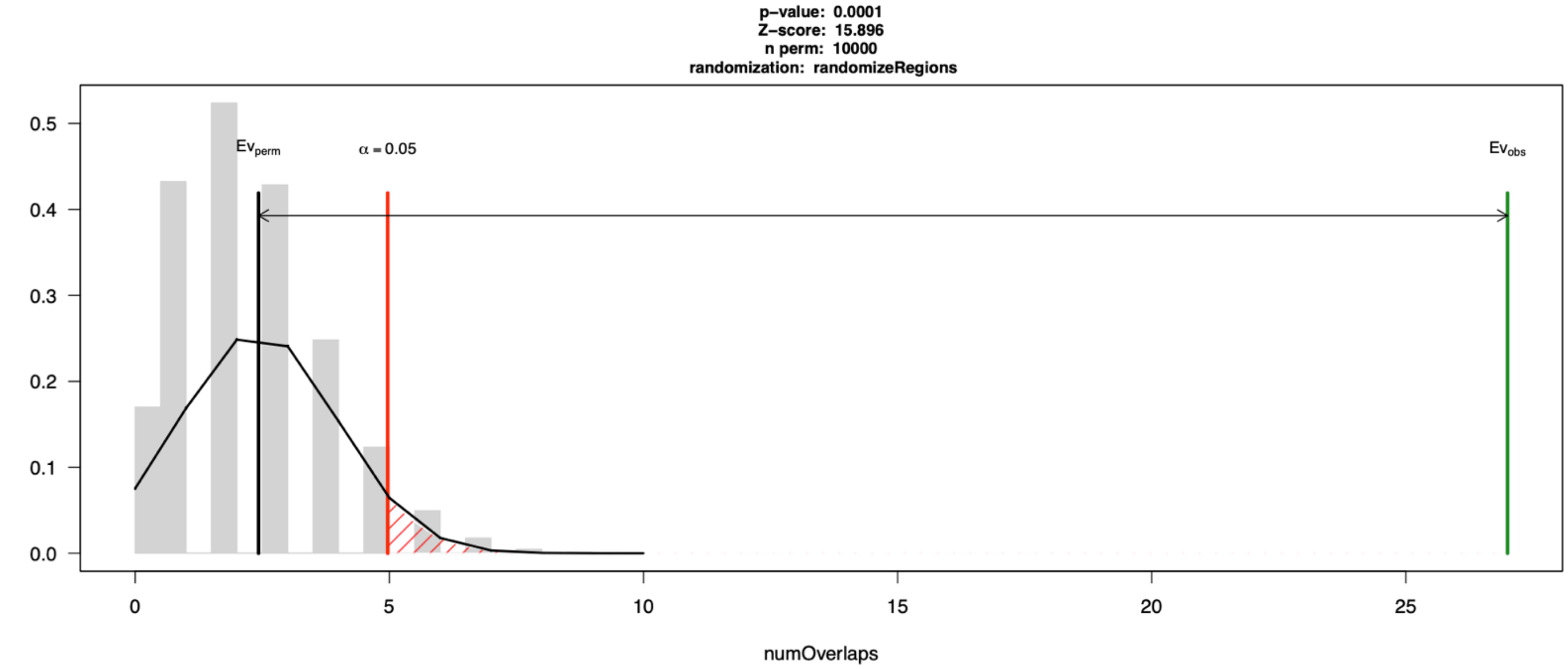

B)

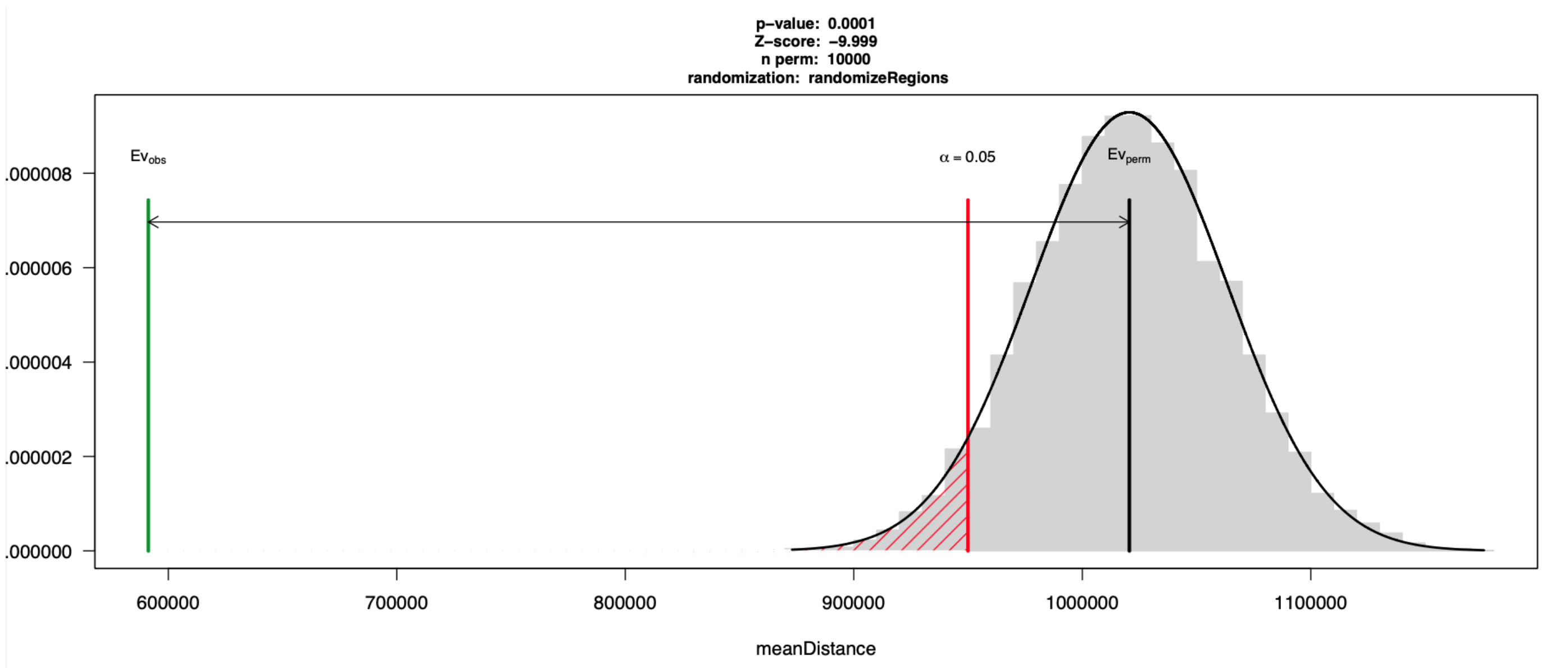

Supplementary Figure 12: Plots of the significance of region level overlap between Male cfDNA ASD DMRs and Male Placenta ASD DMRs as determined by permutations testing with respect to **A)** the number of overlaps between the two DMR sets and **B)** the mean distance between the DMRs in those two sets. Y-axis is probability density on both plots.

A)

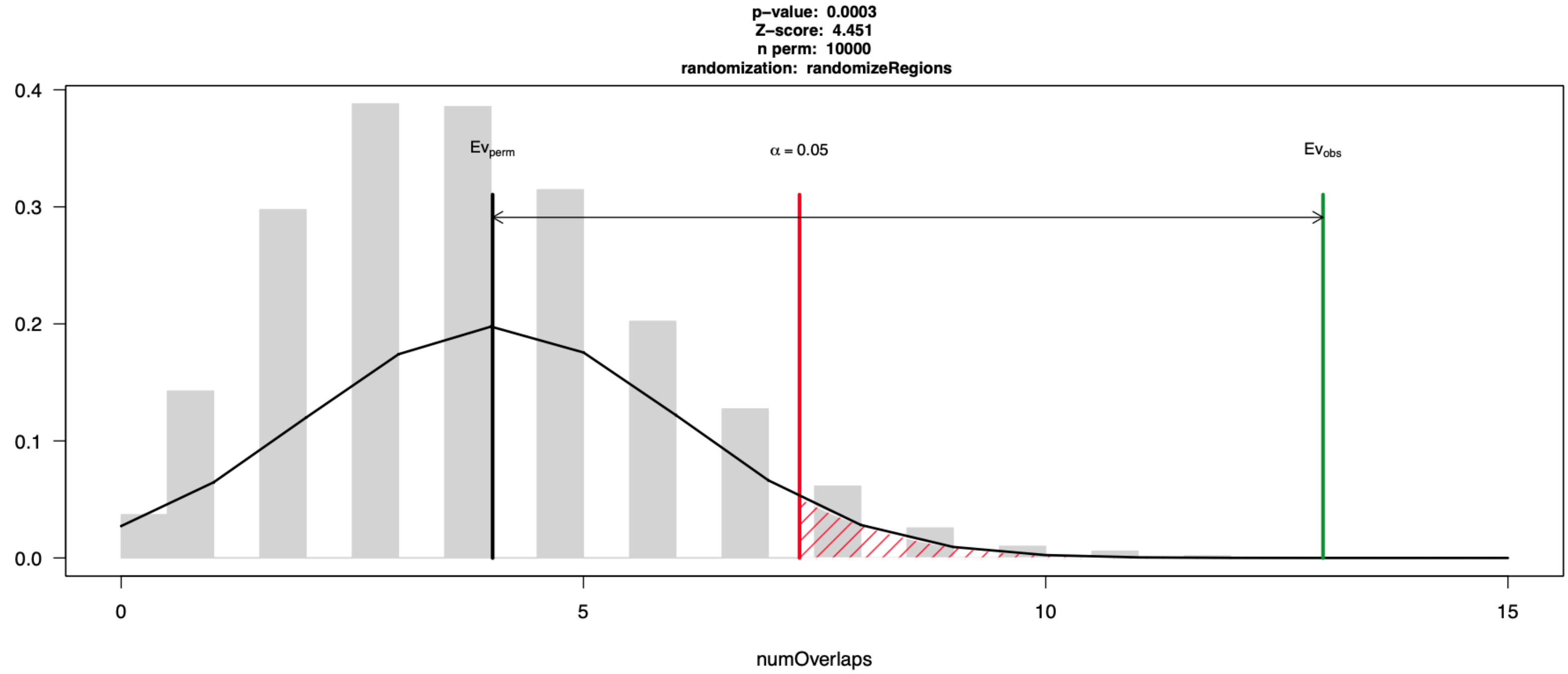

B)

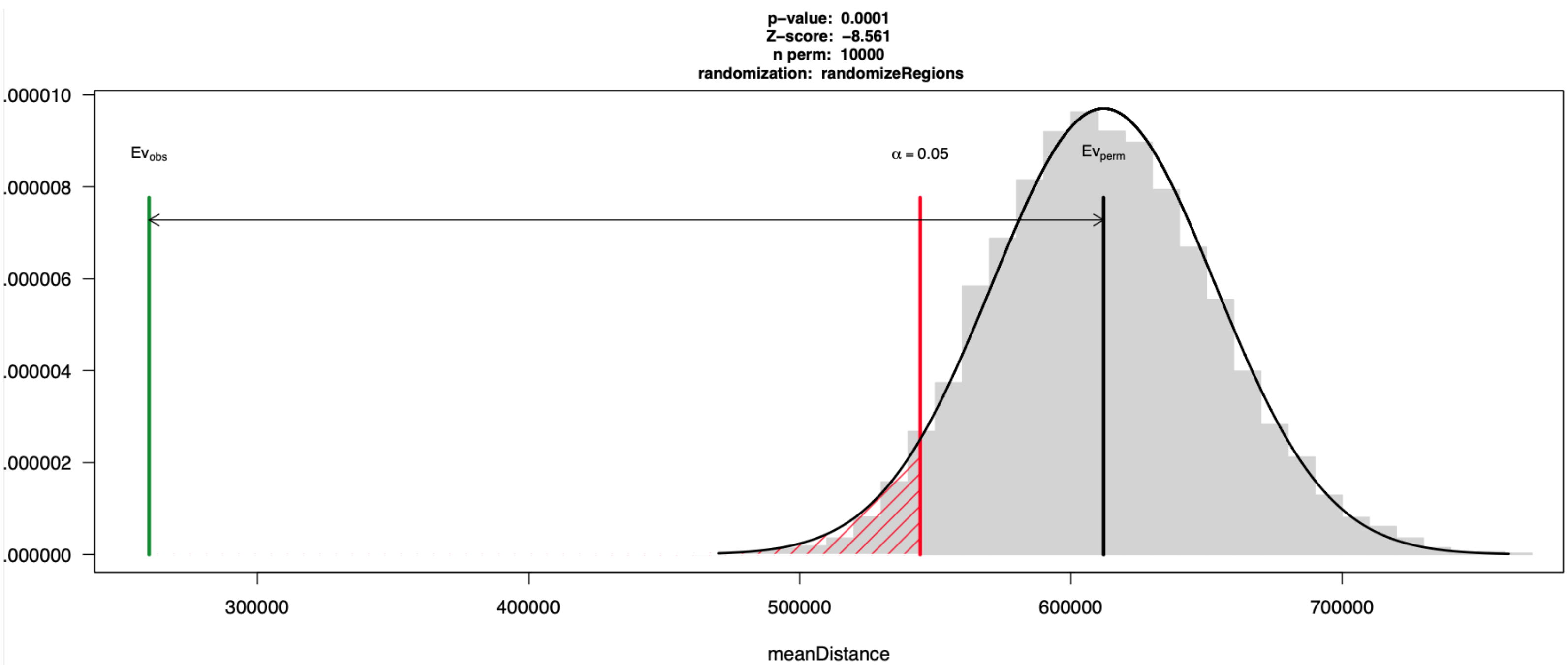

Supplementary Figure 13: Plots of the significance of region level overlap between Male cfDNA ASD DMRs and Male Cortex ASD DMRs as determined by permutations testing with respect to **A)** the number of overlaps between the two DMR sets and **B)** the mean distance between the DMRs in those two sets. Y-axis is probability density on both plots.

A)

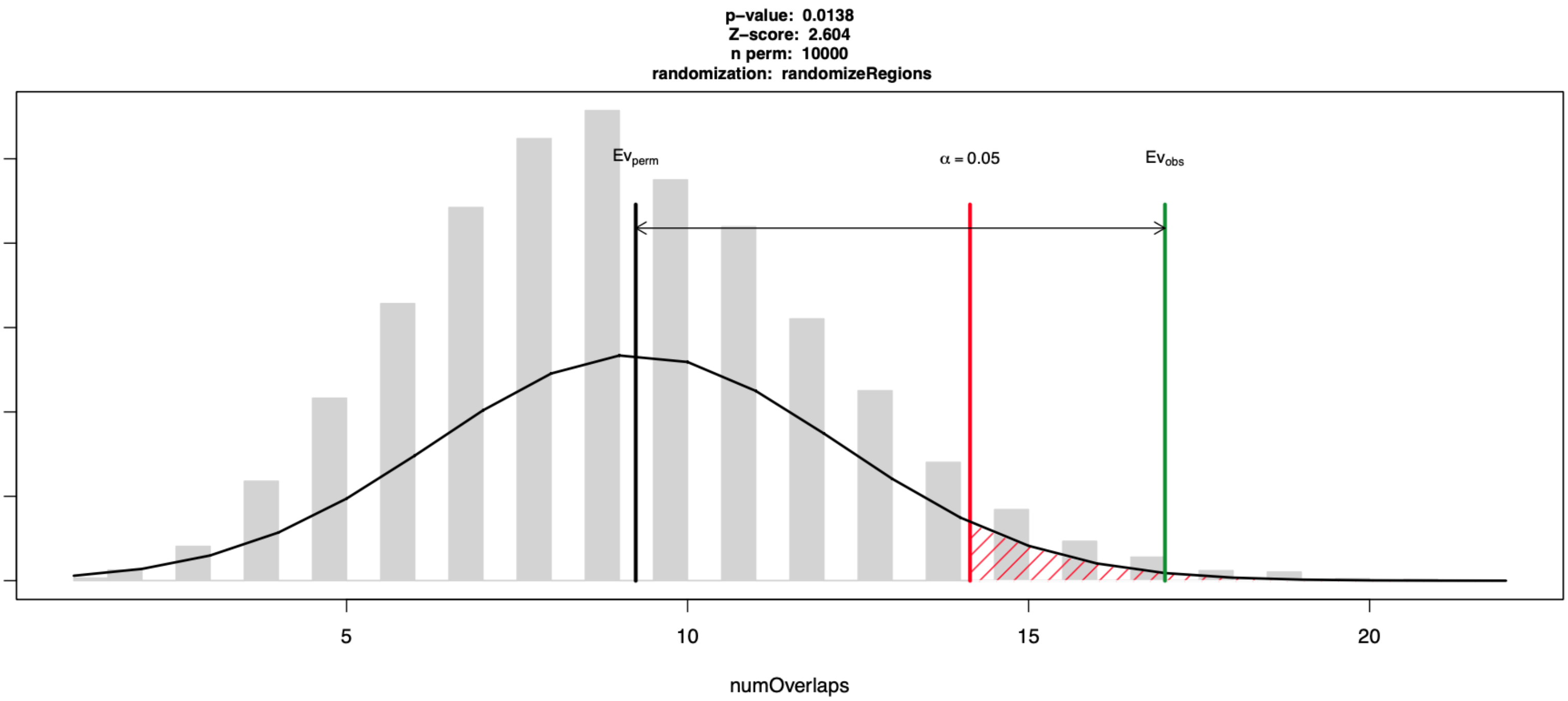

B)

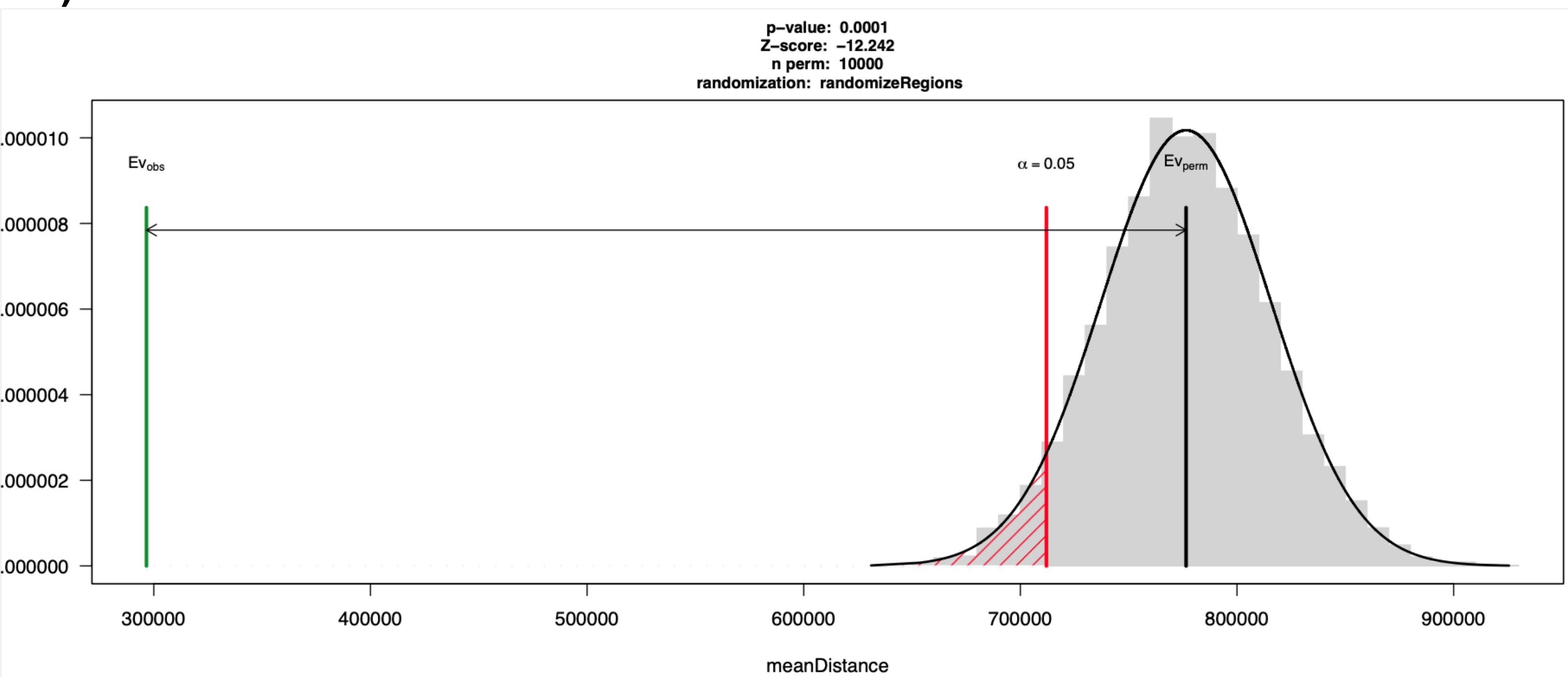

Supplementary Figure 14: Plots of the significance of region level overlap between Male Placenta ASD DMRs and Male Cortex ASD DMRs as determined by permutations testing with respect to **A)** the number of overlaps between the two DMR sets and **B)** the mean distance between the DMRs in those two sets. Y-axis is probability density on both plots.

A)

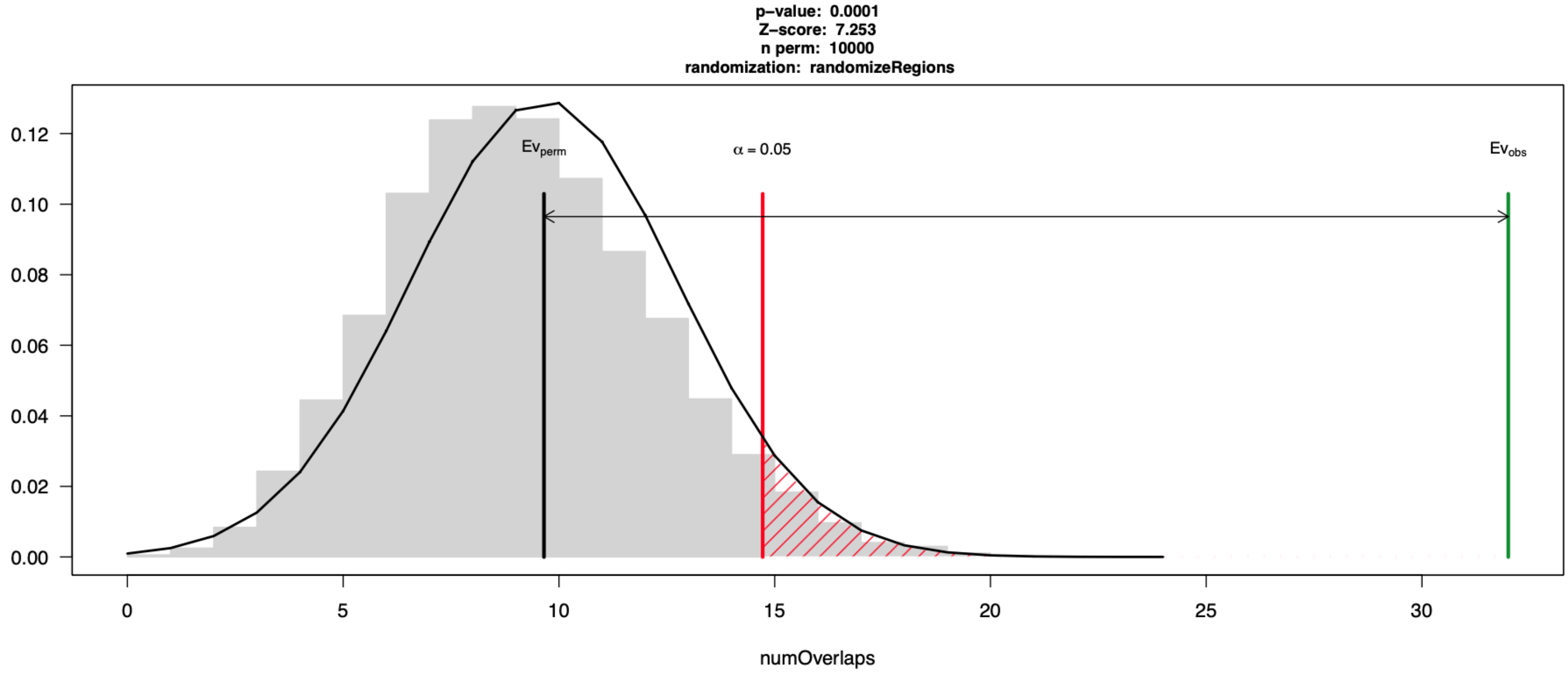

B)

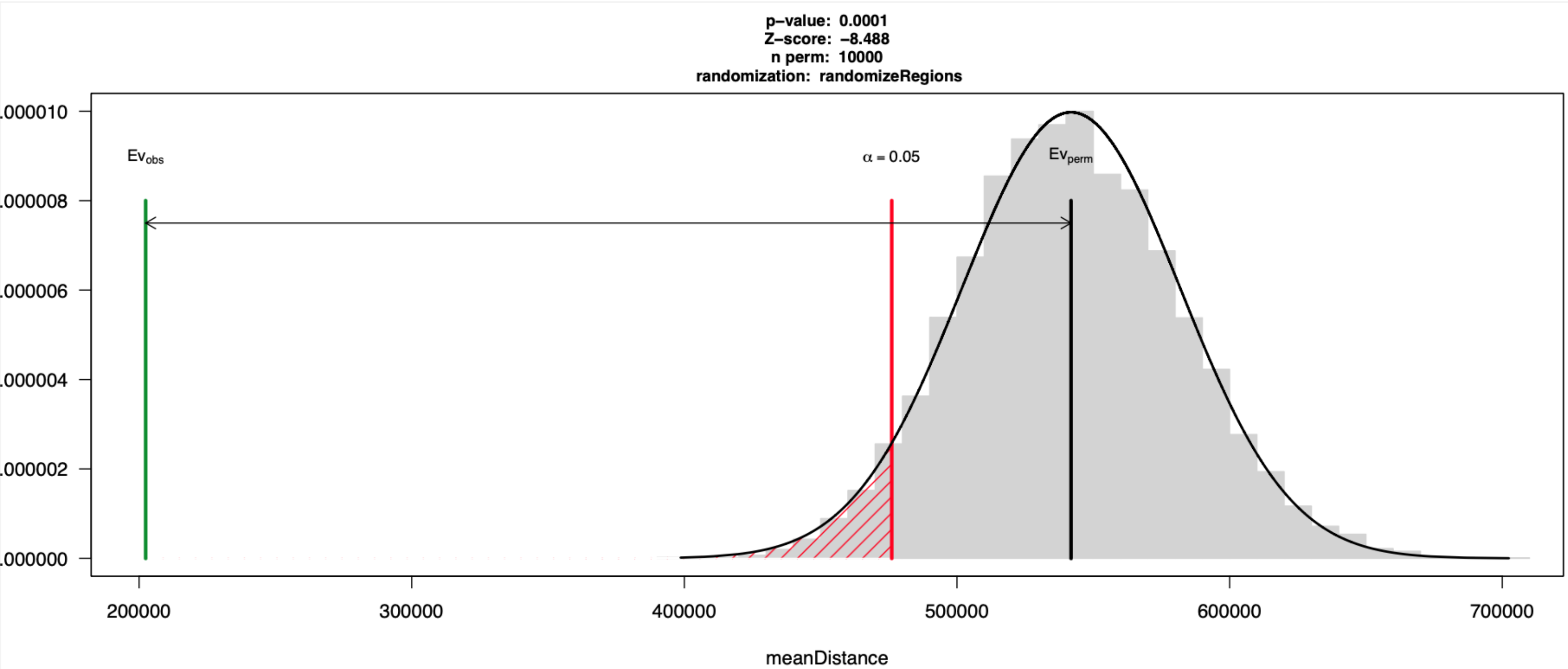

Supplementary Figure 15: Plots of the significance of region level overlap between Female cfDNA ASD DMRs and Female Placenta ASD DMRs as determined by permutations testing with respect to **A)** the number of overlaps between the two DMR sets and **B)** the mean distance between the DMRs in those two sets. Y-axis is probability density on both plots.

A)

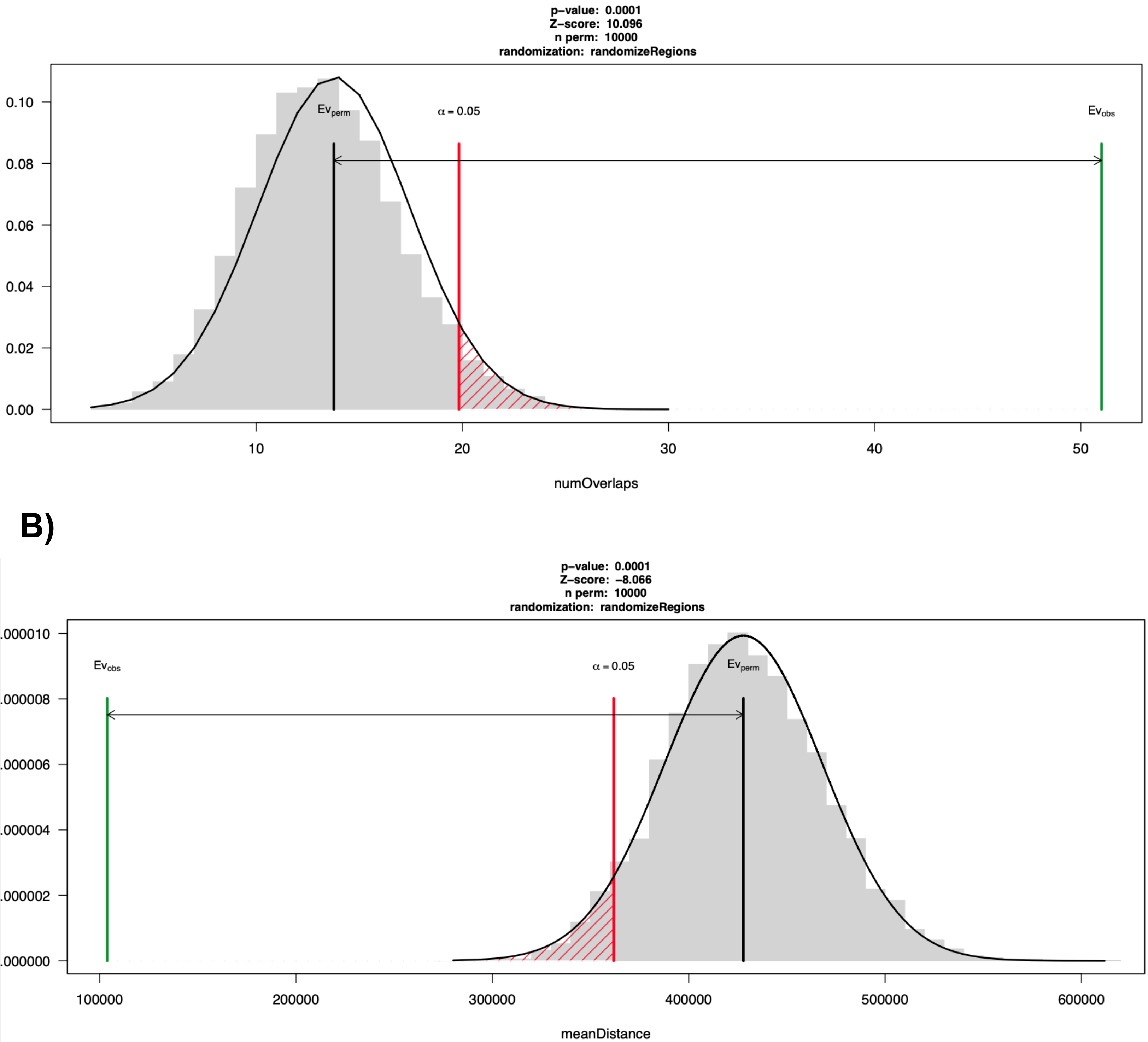

B)

Supplementary Figure 16: Plots of the significance of region level overlap between Female cfDNA ASD DMRs and Female Cortex ASD DMRs as determined by permutations testing with respect to **A)** the number of overlaps between the two DMR sets and **B)** the mean distance between the DMRs in those two sets. Y-axis is probability density on both plots.

A)

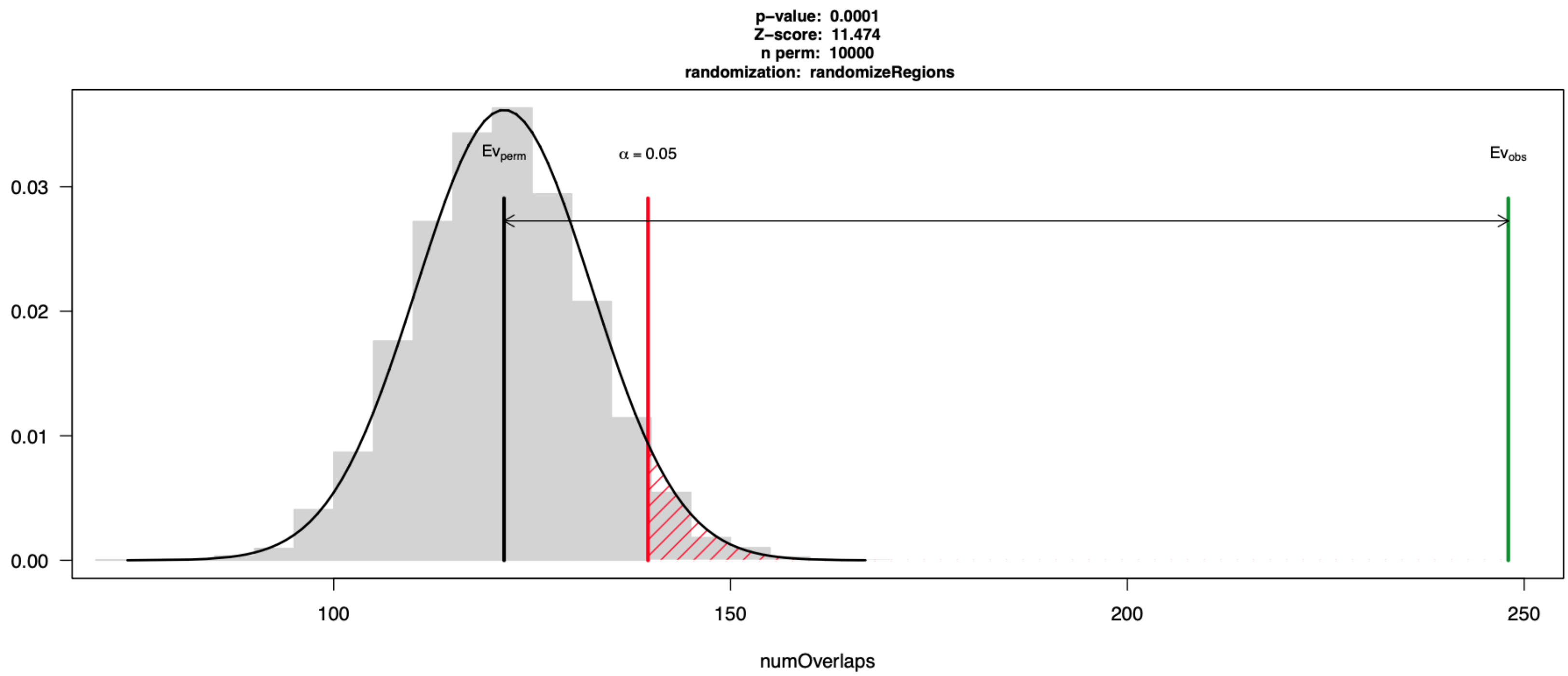

B)

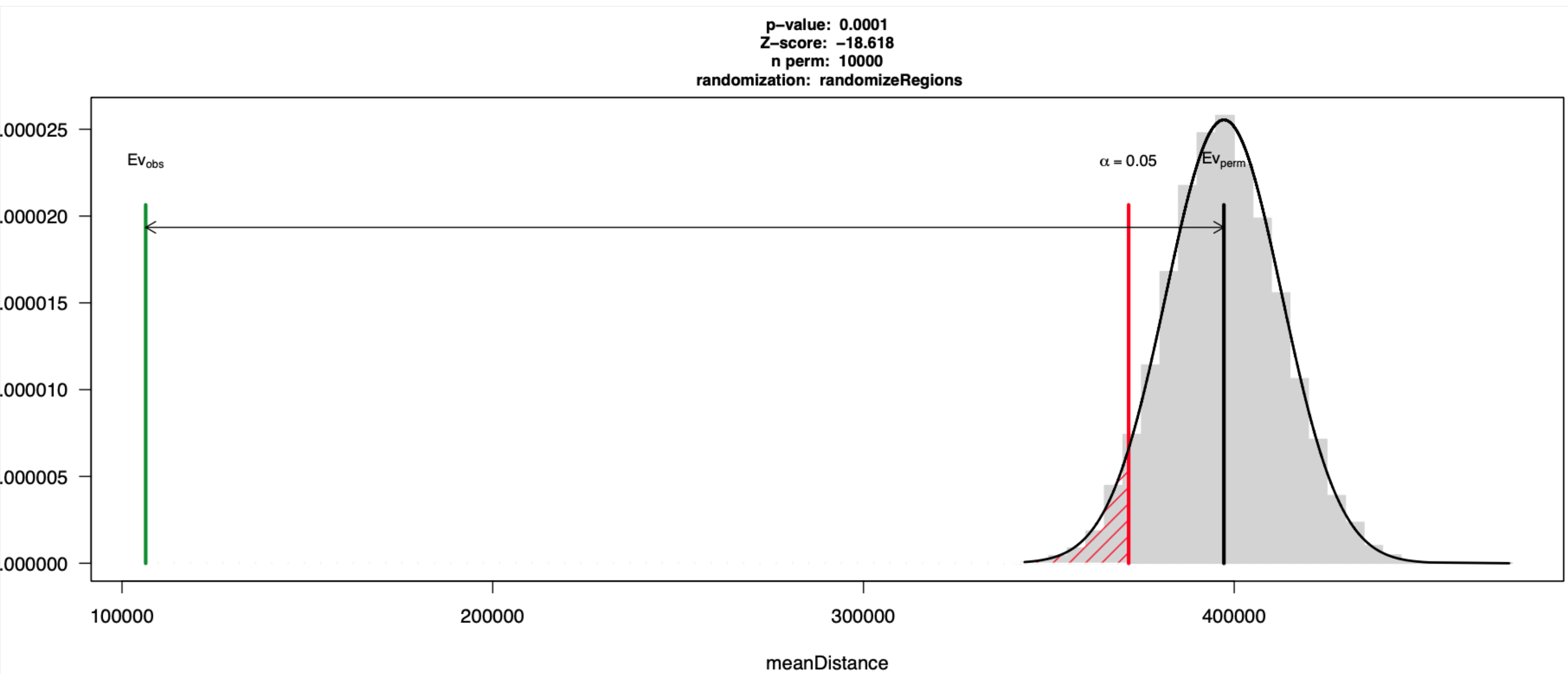

Supplementary Figure 17: Plots of the significance of region level overlap between Female Placenta ASD DMRs and Female Cortex ASD DMRs as determined by permutations testing with respect to **A)** the number of overlaps between the two DMR sets and **B)** the mean distance between the DMRs in those two sets. Y-axis is probability density on both plots.

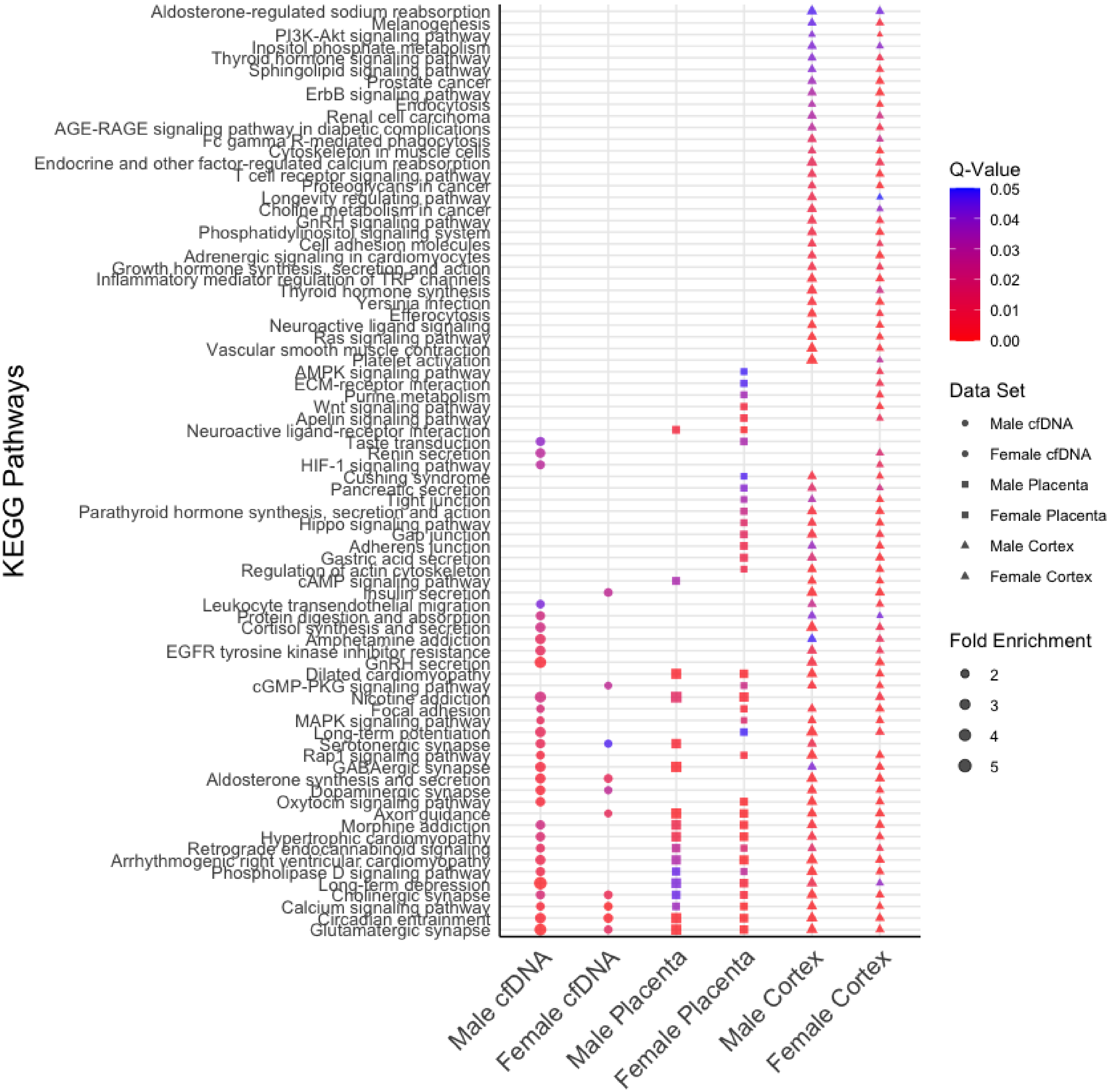

Supplementary Figure 18: The results of KEGG pathway enrichment analyses for all six ASD DMR gene lists, sex-stratified. Terms are ordered bottom to top on the y-axis by the number of DMR gene lists they are significantly enriched in. Significance determined by Fisher's Exact Test with BH correction.

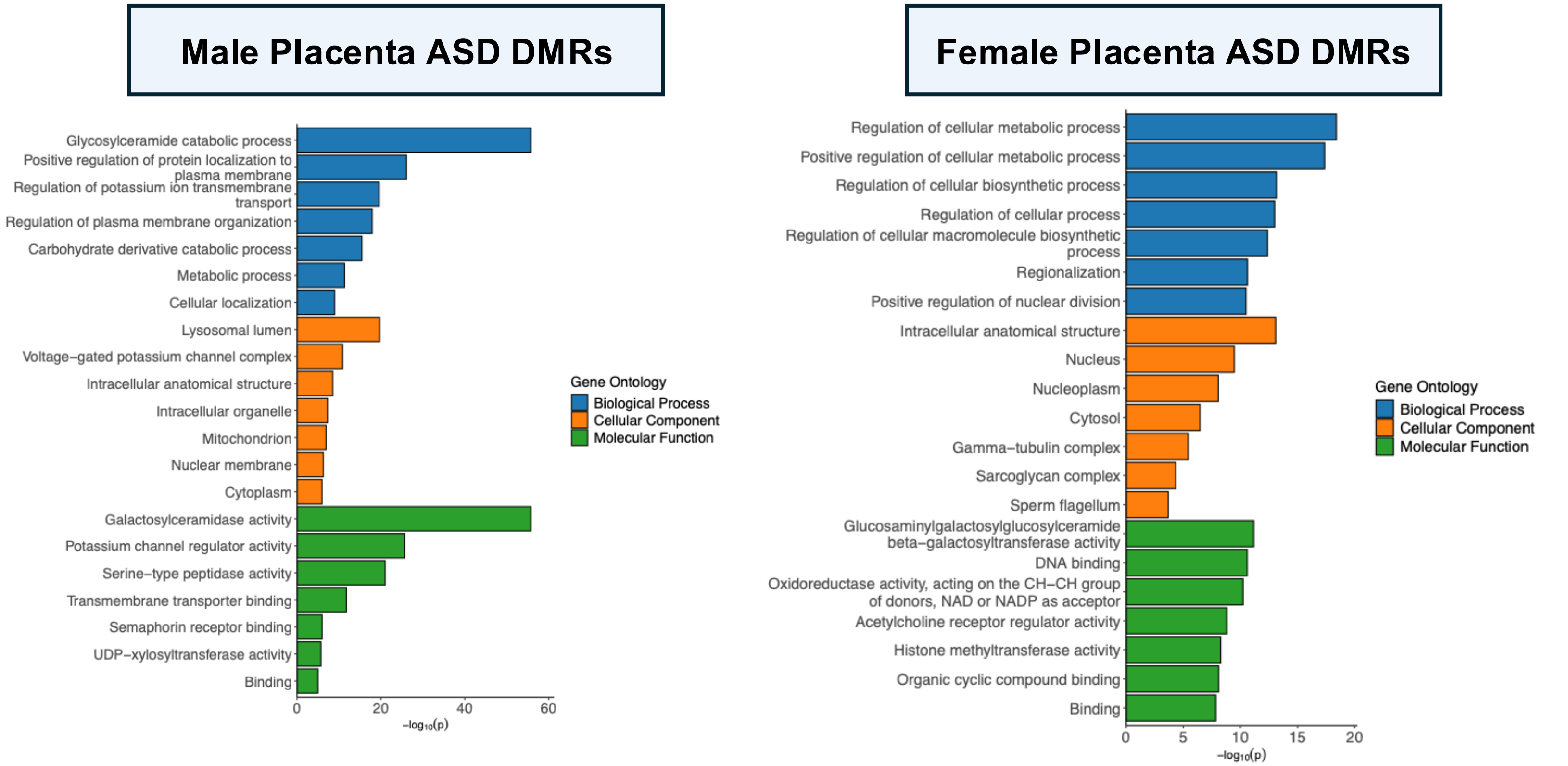

Supplementary Figure 19: The results of Gene ontology (GO) term enrichment analyses for male (n = 2,678) and female (n = 8,447) placenta ASD DMR gene lists, as performed by GREAT. The top seven most significantly enriched terms for each of the three gene ontologies are shown.

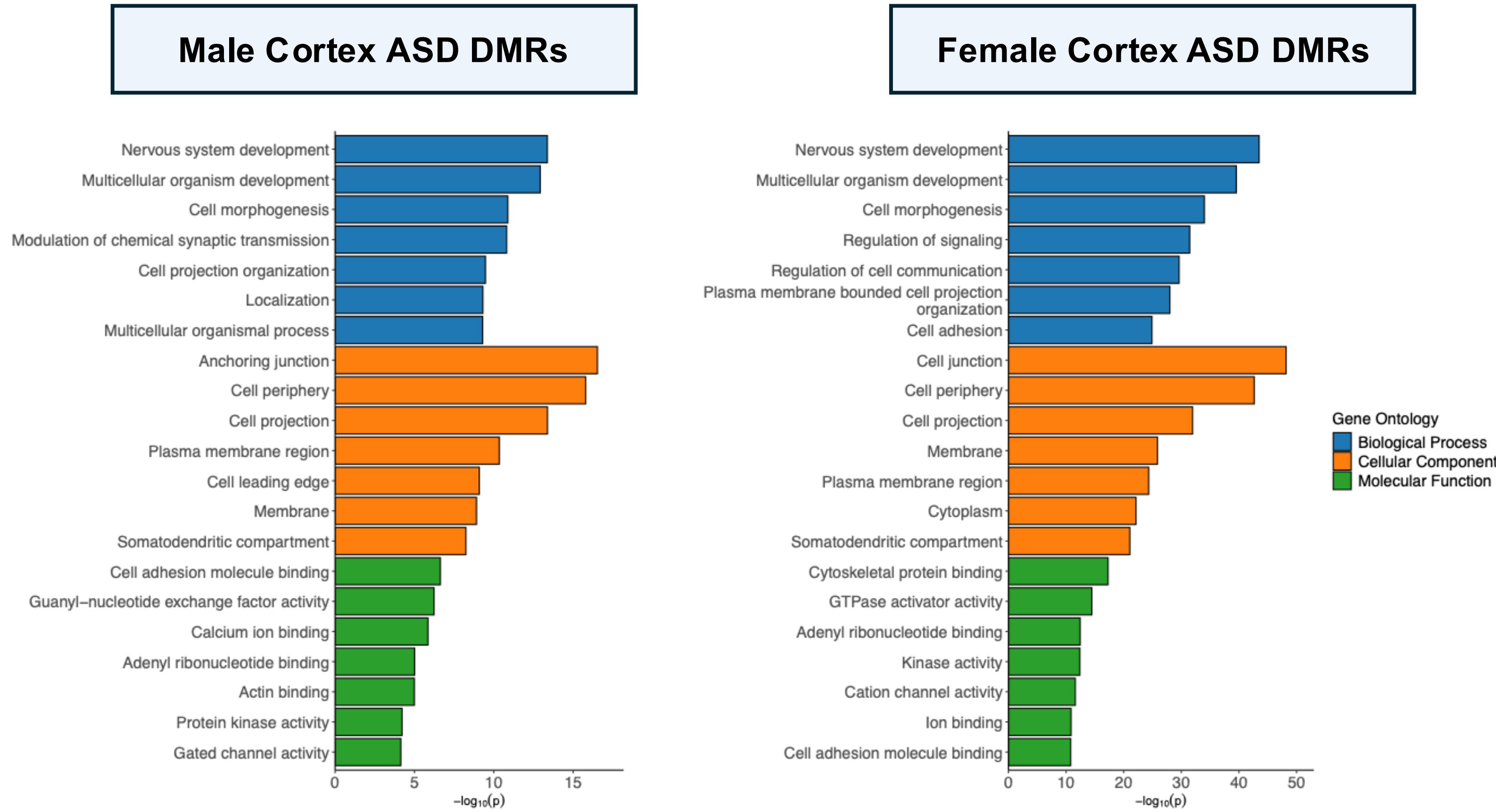

Supplementary Figure 20: The results of Gene ontology (GO) term enrichment analyses for male (n = 5,191) and female (n = 16,644) cortex ASD DMR gene lists, as performed by GREAT. The top seven most significantly enriched terms for each of the three gene ontologies are shown.

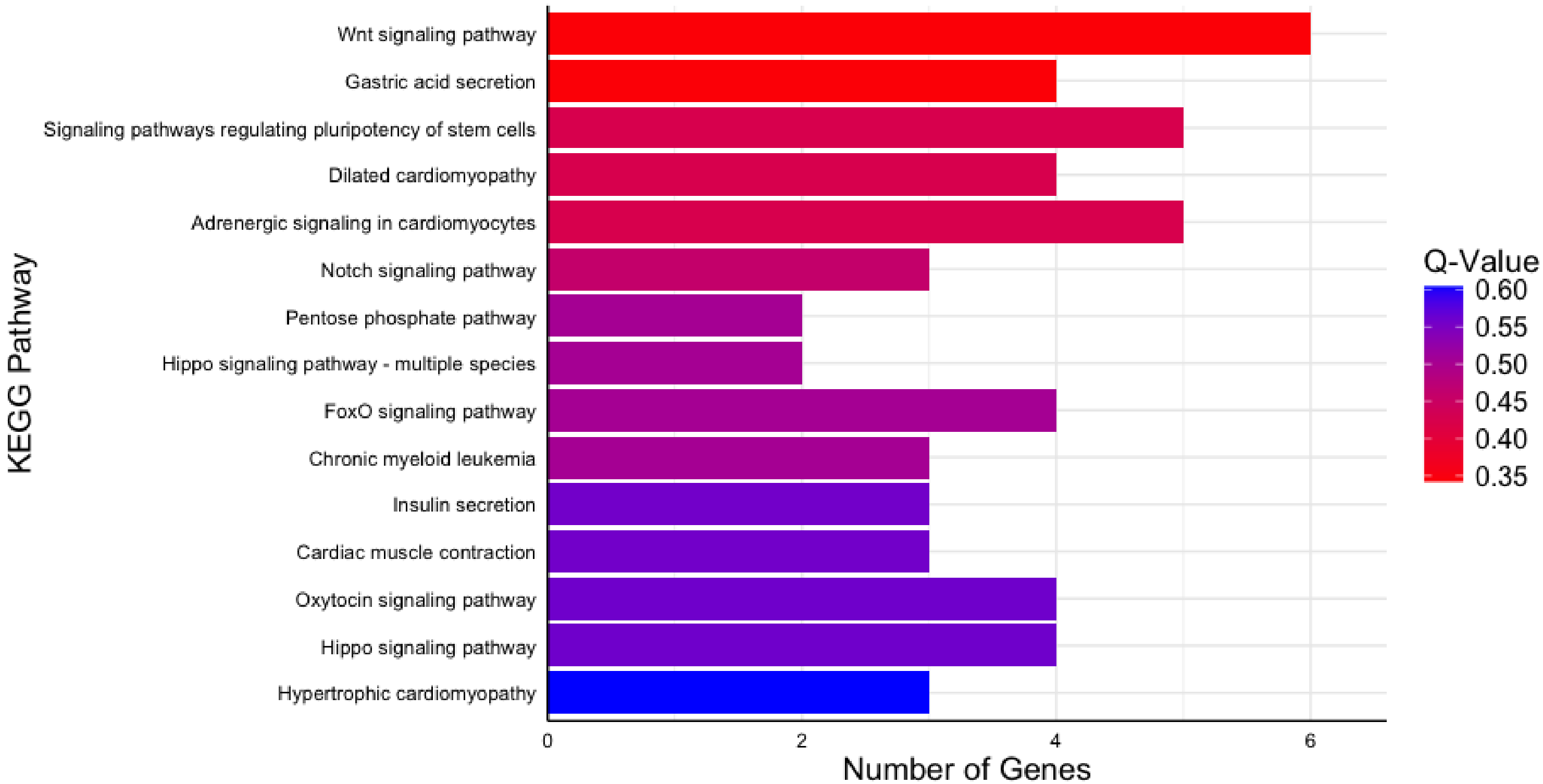

Supplementary Figure 21: Barplot of the top 15 most significantly enriched for KEGG pathways in the list of 227 genes whose overlap is specific to female cfDNA, placenta, and cortex ASD DMRs. No pathway enrichment passed a significance threshold of $q < 0.05$.

A)

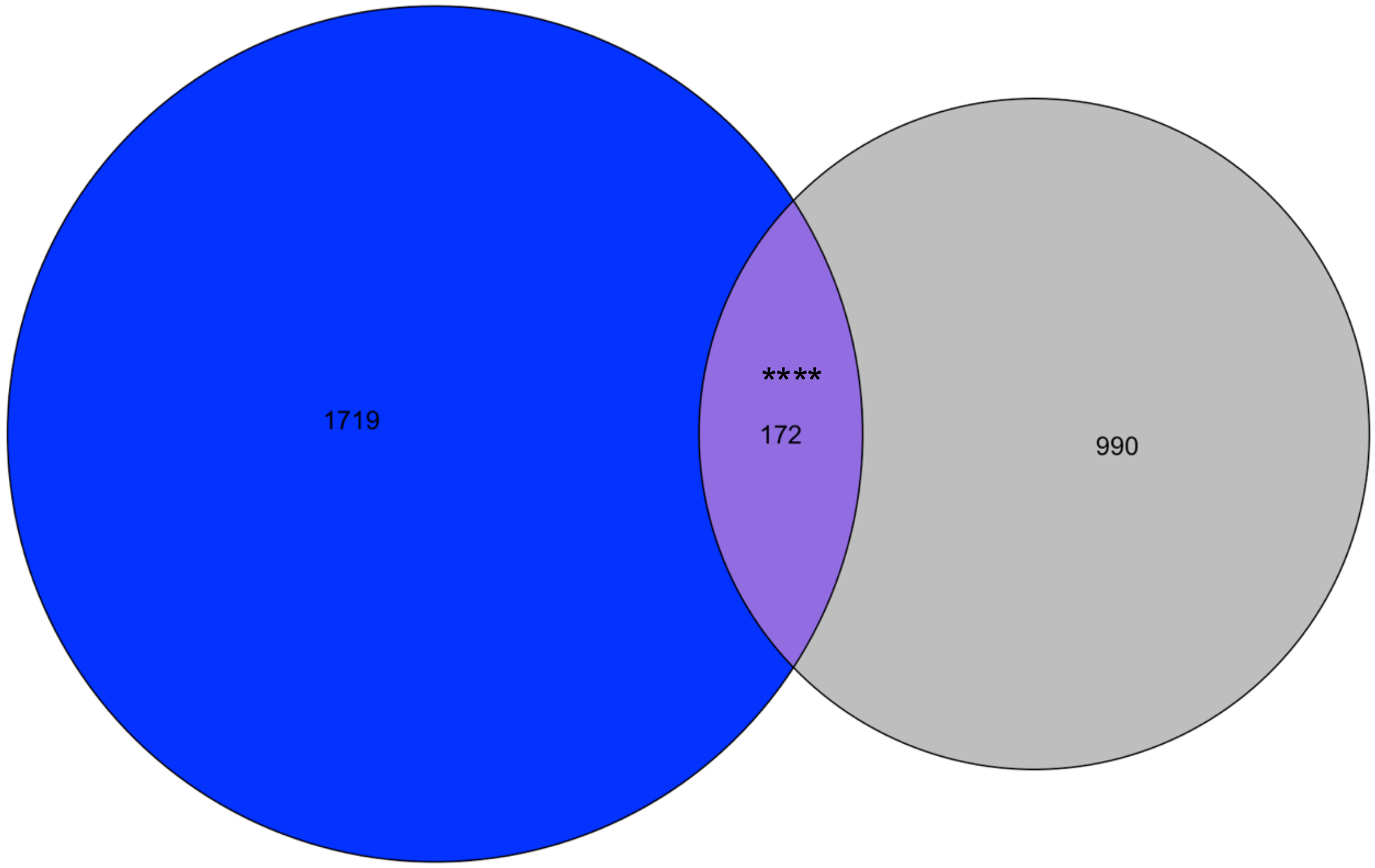

p-value = 6.1×10^{-6}

B)

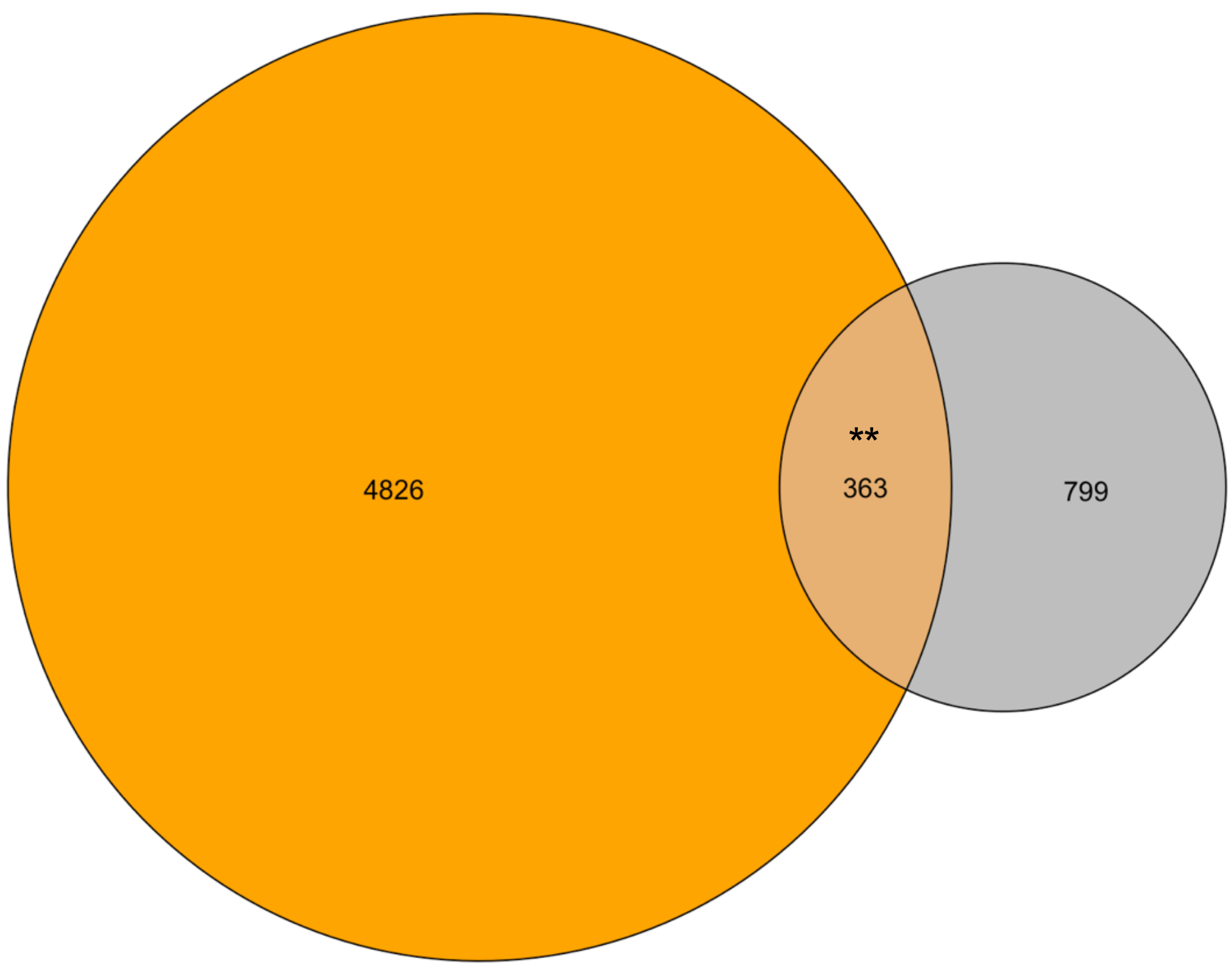

p-value = 0.0014

Supplementary Figure 22: Venn diagrams of the overlapping genes between the SFARI gene list and **A)** male placenta ASD DMRs (n = 2,678) and **B)** female placenta ASD DMRs (n = 8,447).

A)

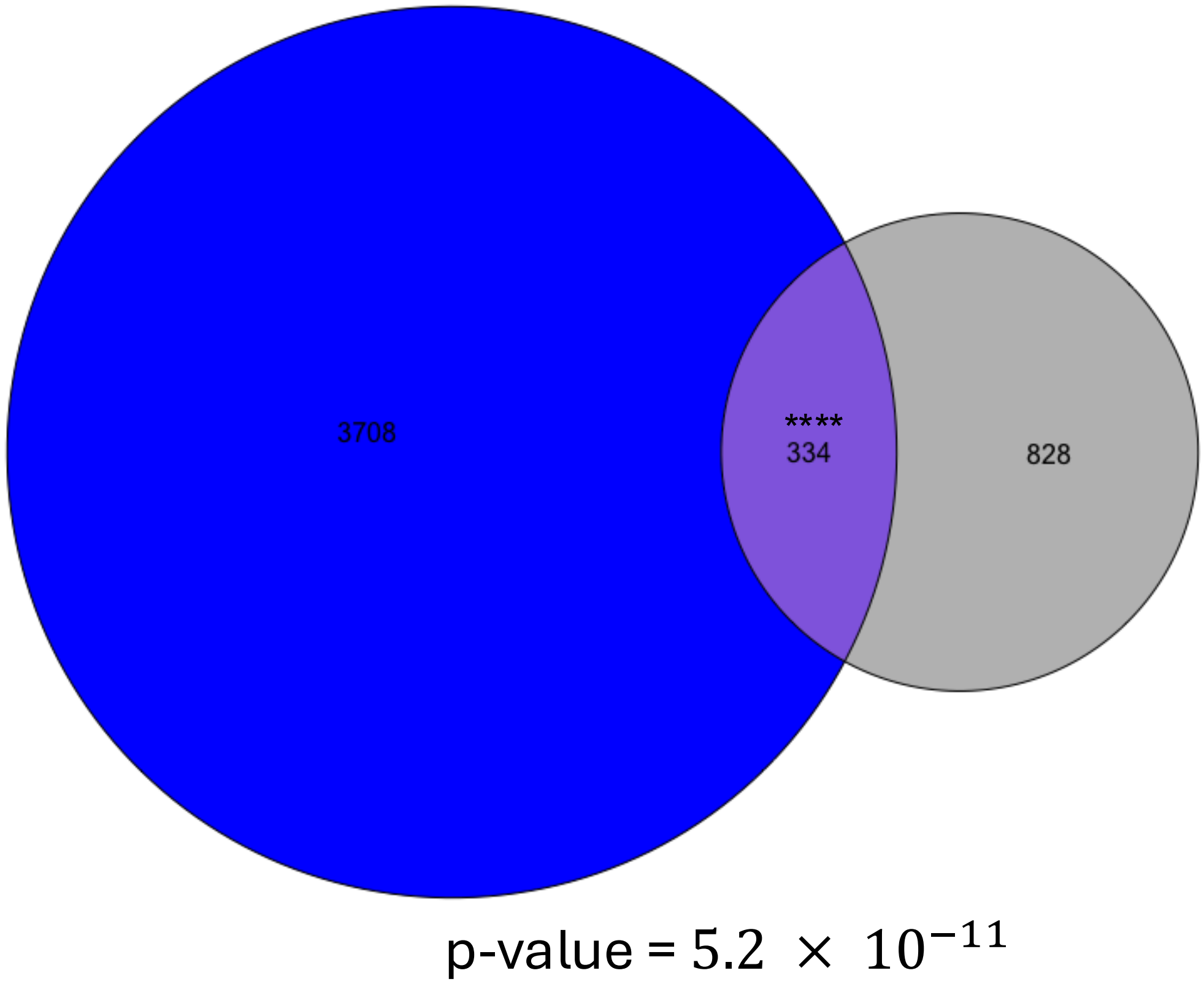

B)

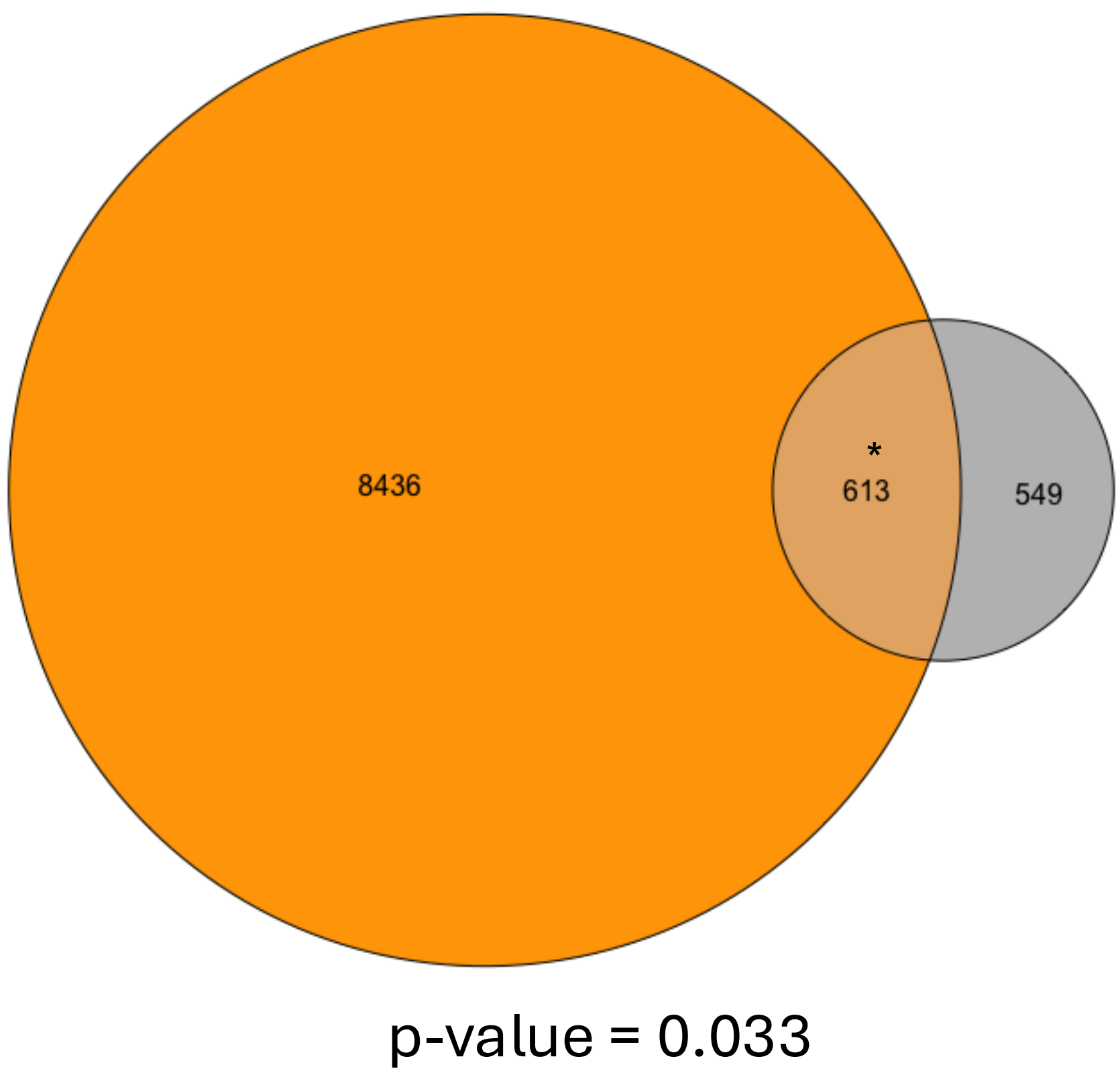

Supplementary Figure 23: Venn diagrams of the overlapping genes between the SFARI gene list and **A)** male cortex ASD DMRs (n = 5,191) and **B)** female cortex ASD DMRs (n = 16,644).

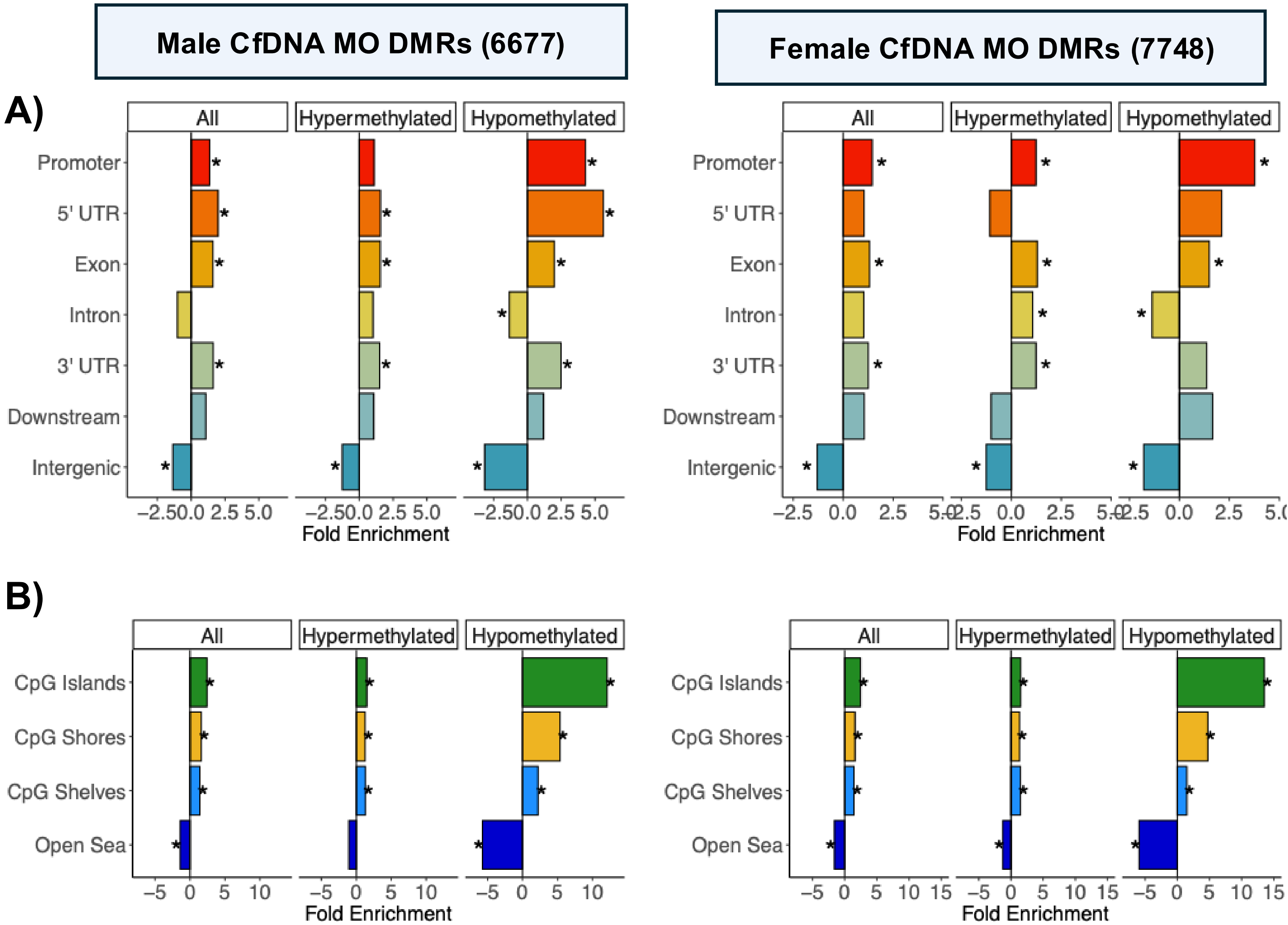

Supplementary Figure 24: Enrichment analyses of male (n = 6,677) and female (n = 7,748) cfDNA MO DMRs (permutation p-value < 0.05) for both **A)** genic annotations and **B)** CpG annotations as performed and plotted by DMRichR. DMRs were compared to background regions for each comparison, with significance determined by Fisher's test with FDR correction (* = q-value < 0.05).

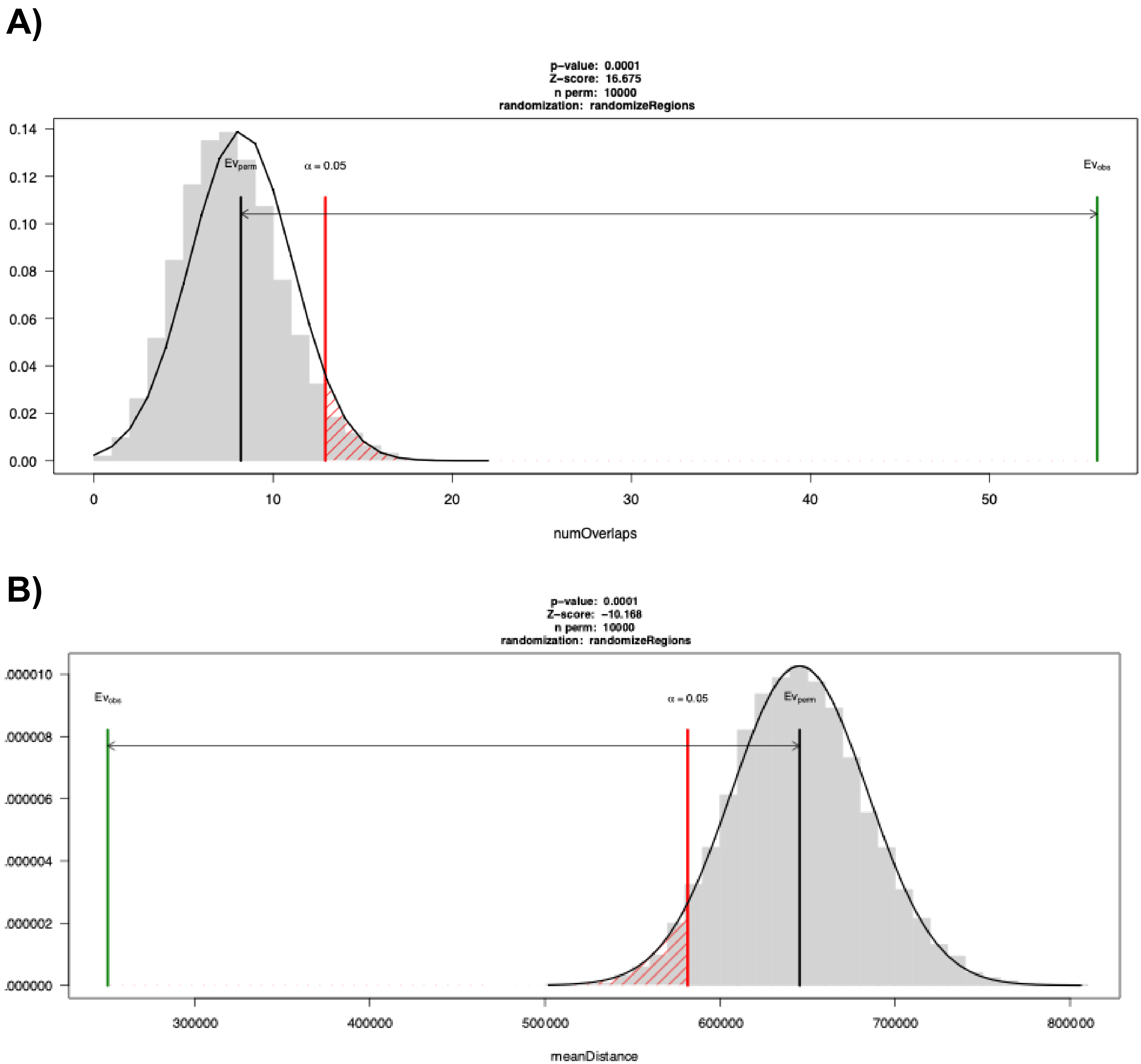

Supplementary Figure 25: Plots of the significance of region level overlap between Male cfDNA ASD and MO DMRs as determined by permutations testing with respect to **A)** the number of overlaps between the two DMR sets and **B)** the mean distance between the DMRs in those two sets. Y-axis is probability density on both plots.

A)

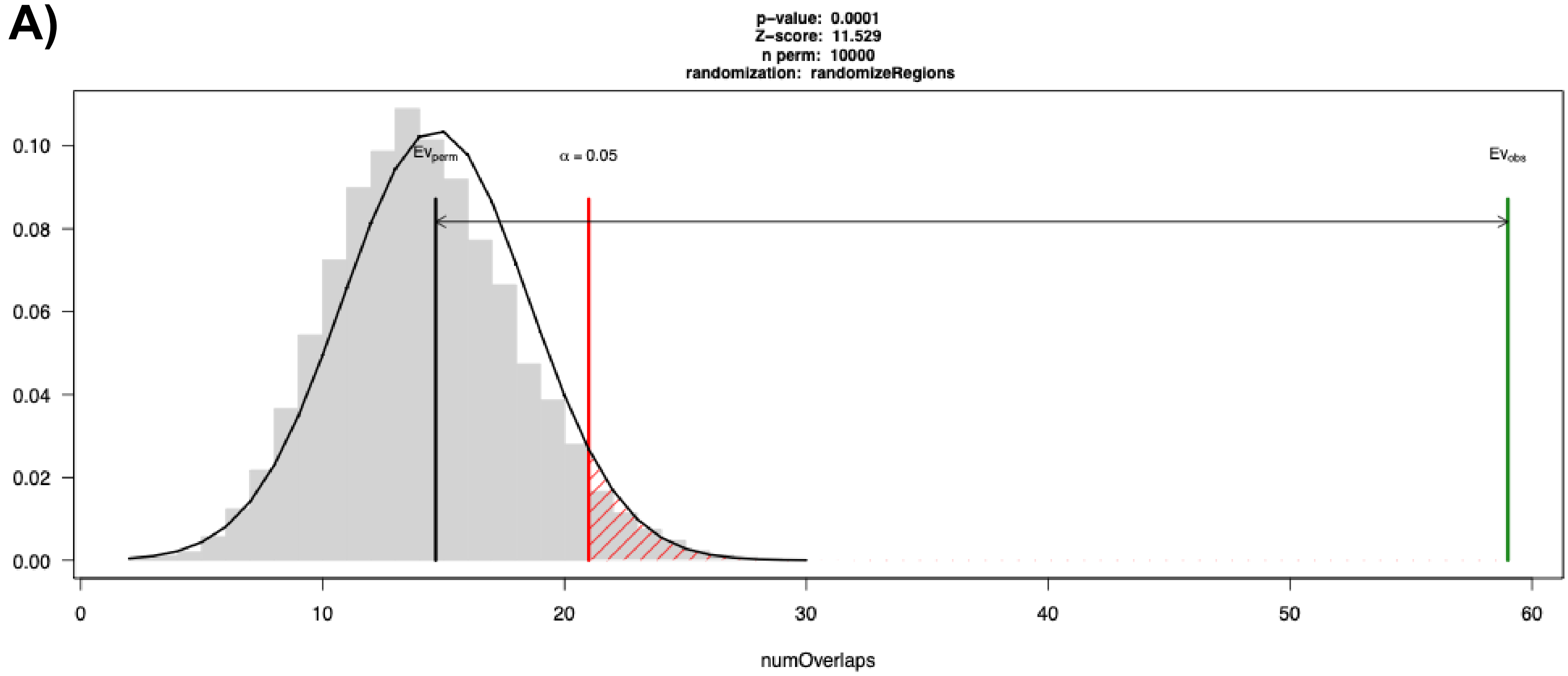

B)

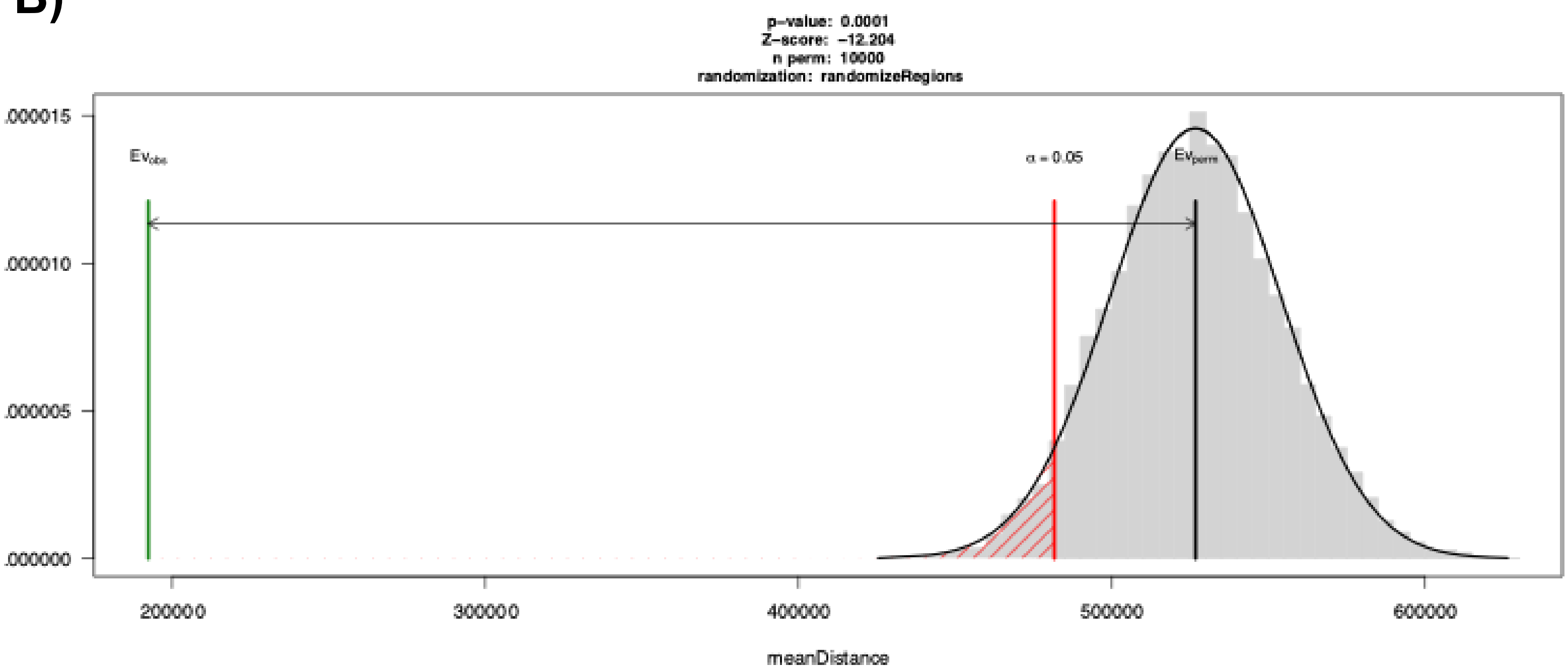

Supplementary Figure 26: Plots of the significance of region level overlap between Female cfDNA ASD and MO DMRs as determined by permutations testing with respect to **A)** the number of overlaps between the two DMR sets and **B)** the mean distance between the DMRs in those two sets. Y-axis is probability density on both plots.

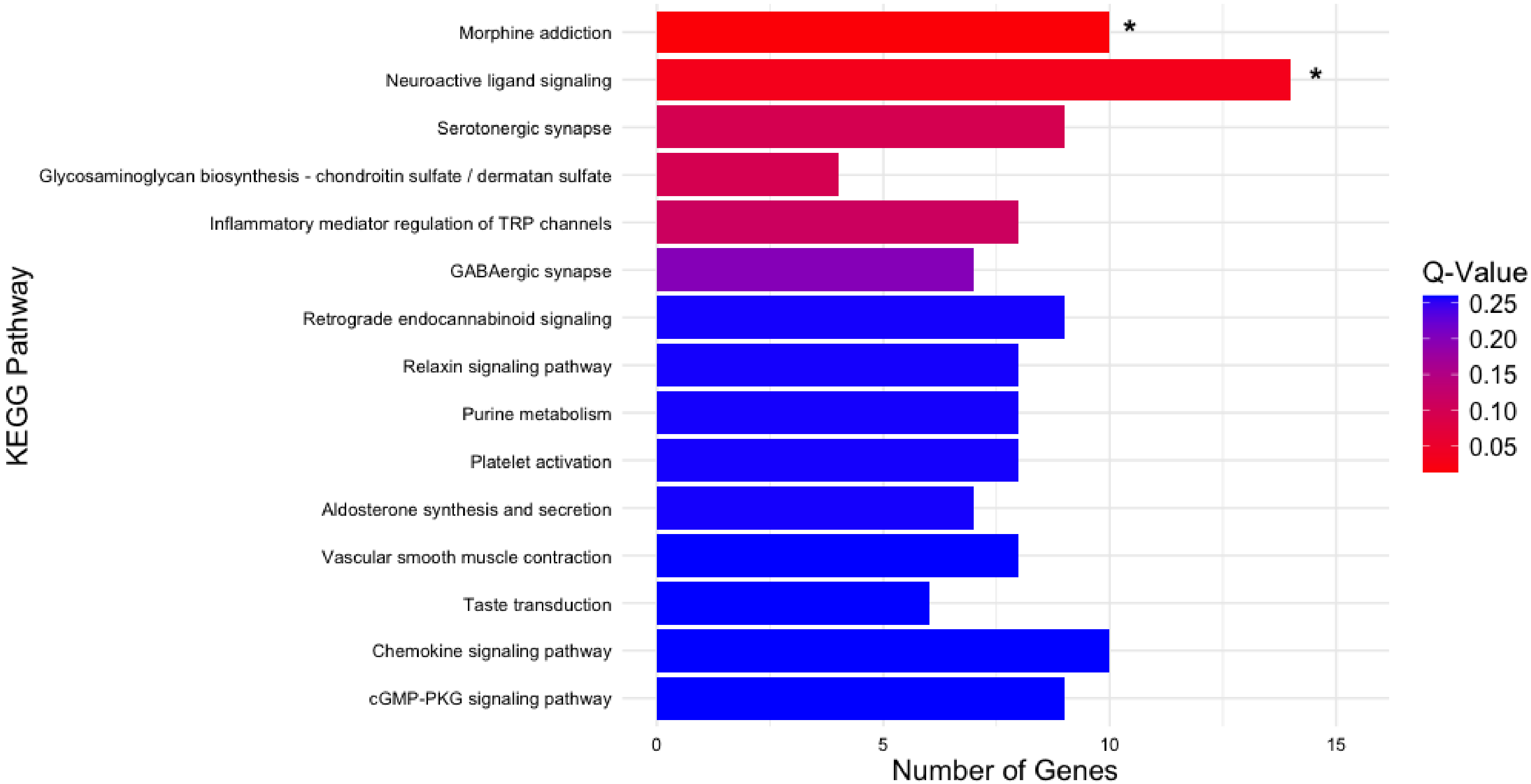

Supplementary Figure 27: Bar plot of the top 15 most significantly enriched for KEGG pathways in the list of 922 genes whose overlap is specific to male (n = 6,677) and female (n = 7,748) MO DMRs and not intersecting with any cfDNA ASD DMRs. Significance determined by Fisher's Exact Test (* $q < 0.05$).

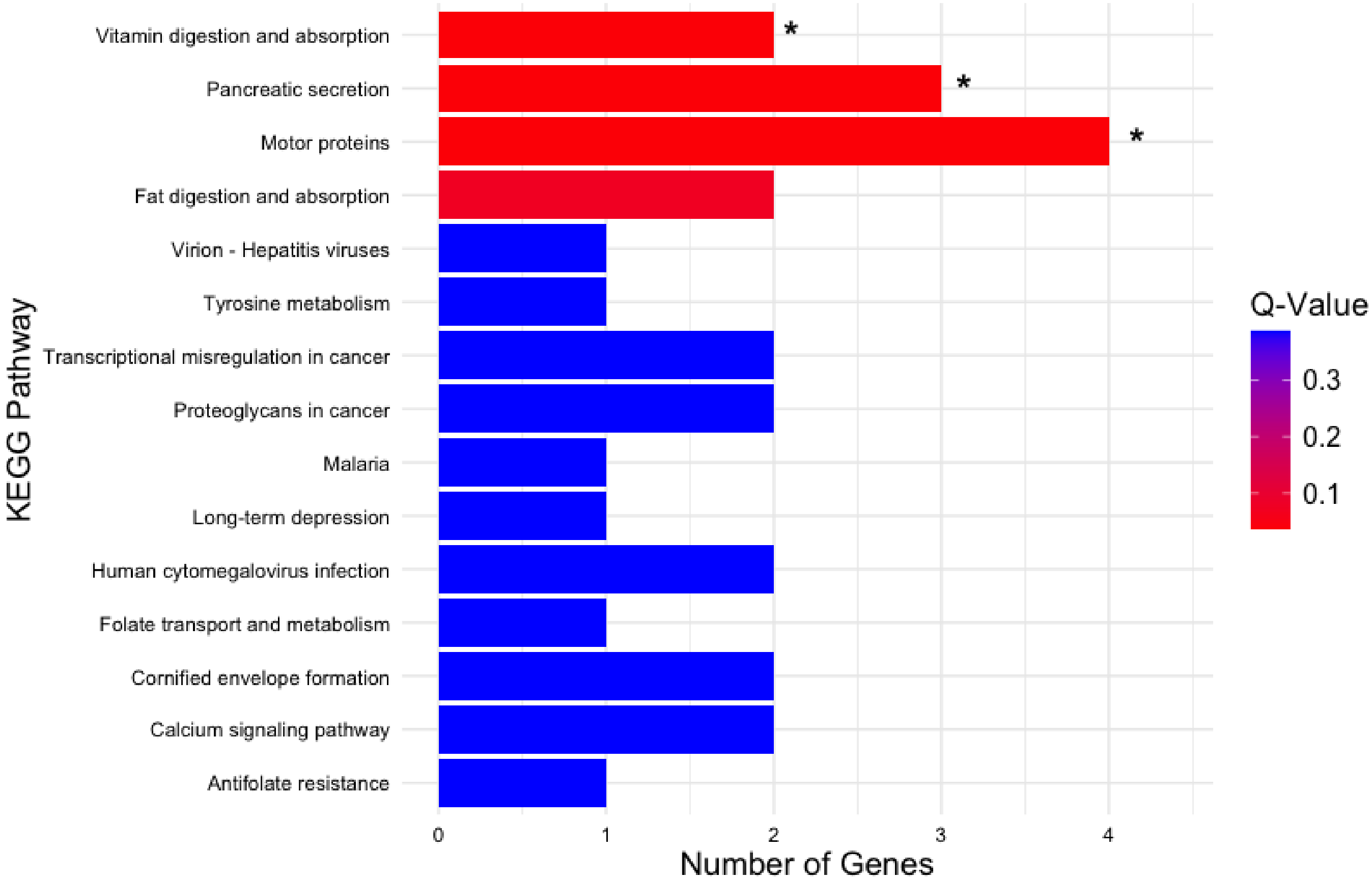

Supplementary Figure 28: Bar plot of the top 15 most significantly enriched for KEGG pathways in the list of 70 genes whose overlap is specific to male and female cfDNA ASD DMRs and not intersecting with MO DMR genes. Significance determined by Fisher’s Exact Test (* $q < 0.05$).

A)

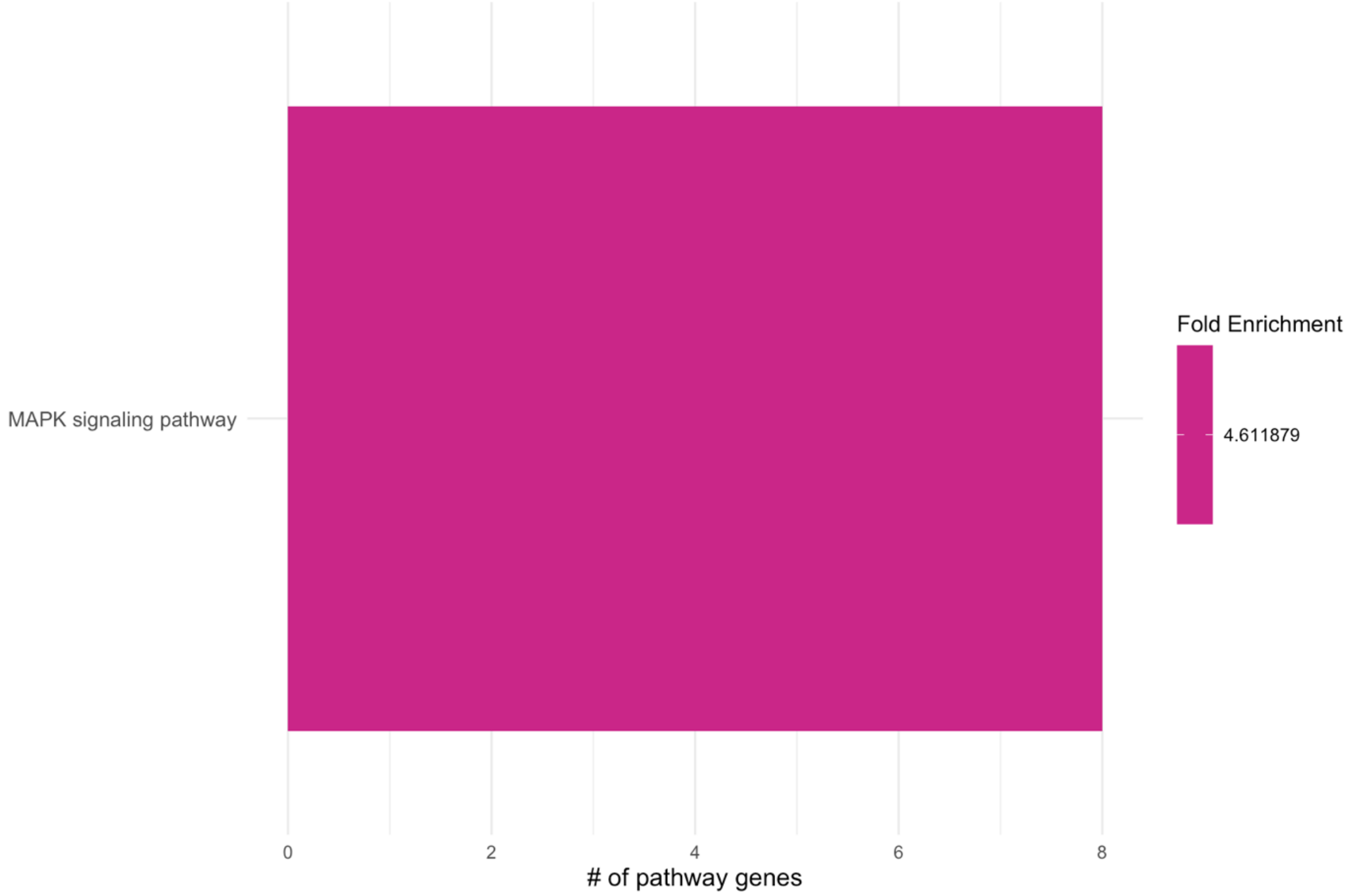

B)

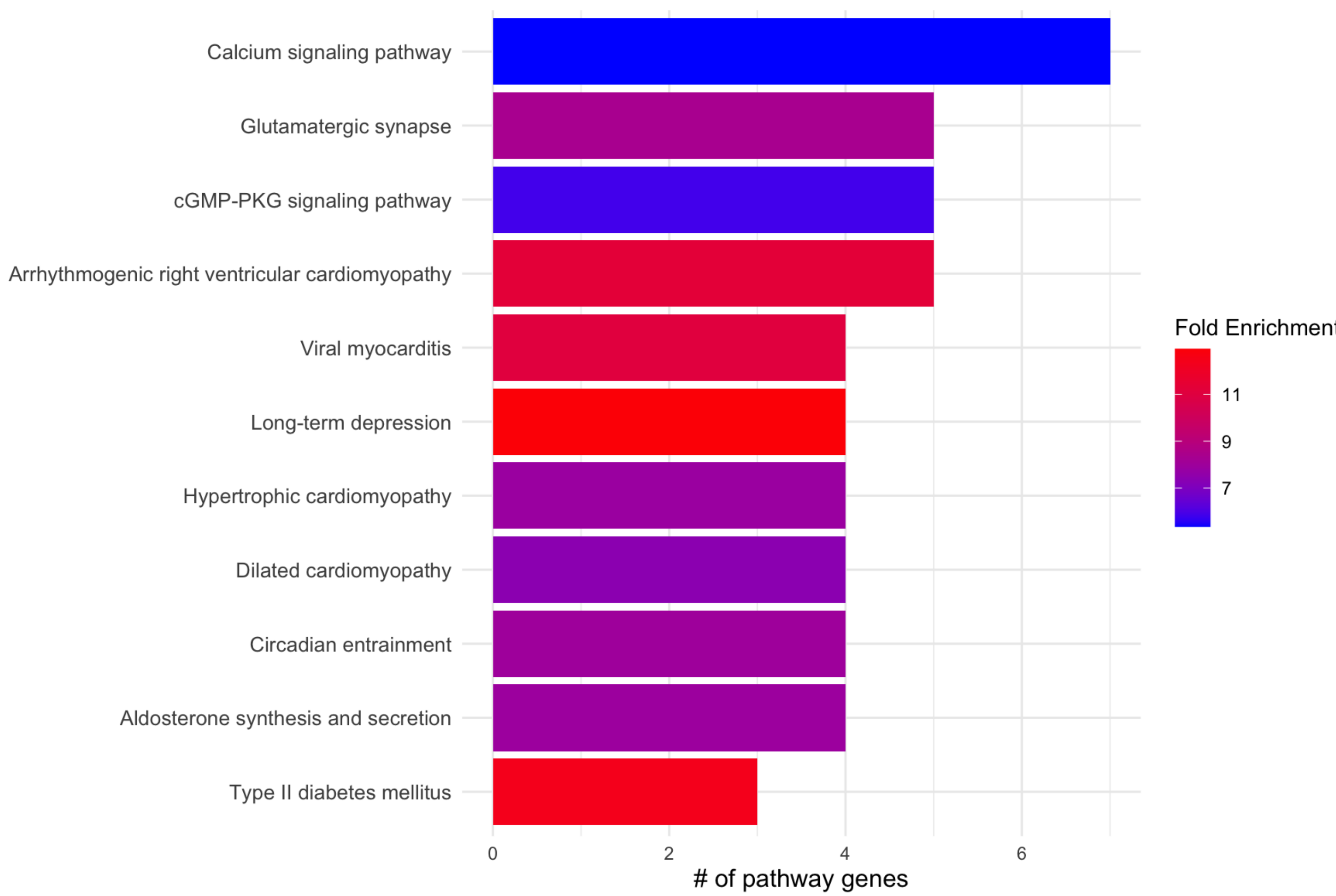

Supplementary Figure 29: Bar plot of all significantly enriched for KEGG pathways in the subset of cfDNA ASD DMR genes shared between male and females with **A)** diverging methylation between sexes and **B)** converging methylation between sexes**.** Significance determined by Fisher's Exact Test (* q < 0.05), and all pathways shown in these plots meet significance standard.

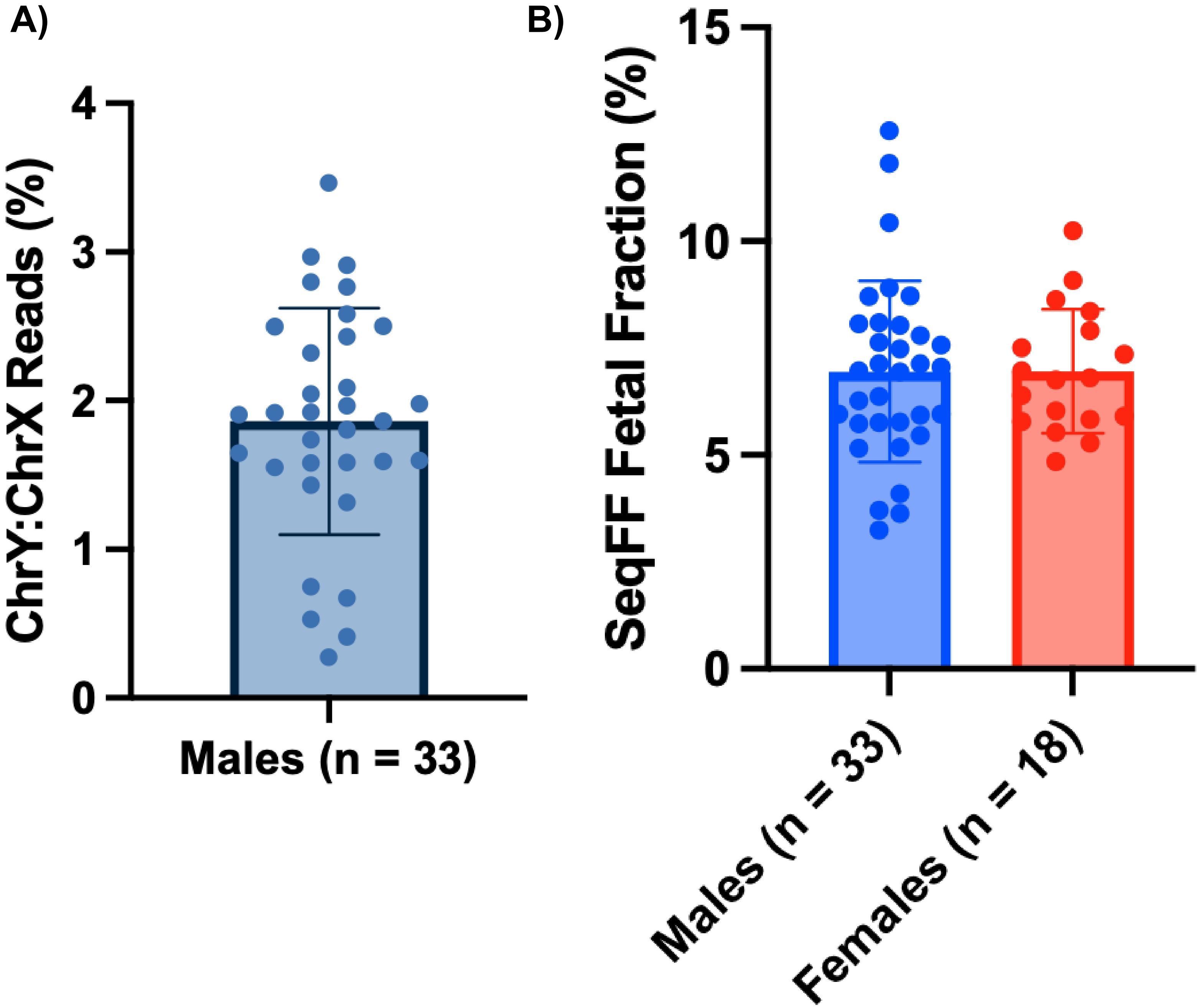

Supplementary Figure 30: Graphs depicting the fraction of cfDNA calculated to be of fetal origin as quantified by **A)** ChrY:ChrX ratios in the fetal male samples (n = 33) with error bars denoting standard deviation from the mean and **B)** SeqFF in all samples (33 males, 18 females) with error bars denoting standard deviation from the mean.

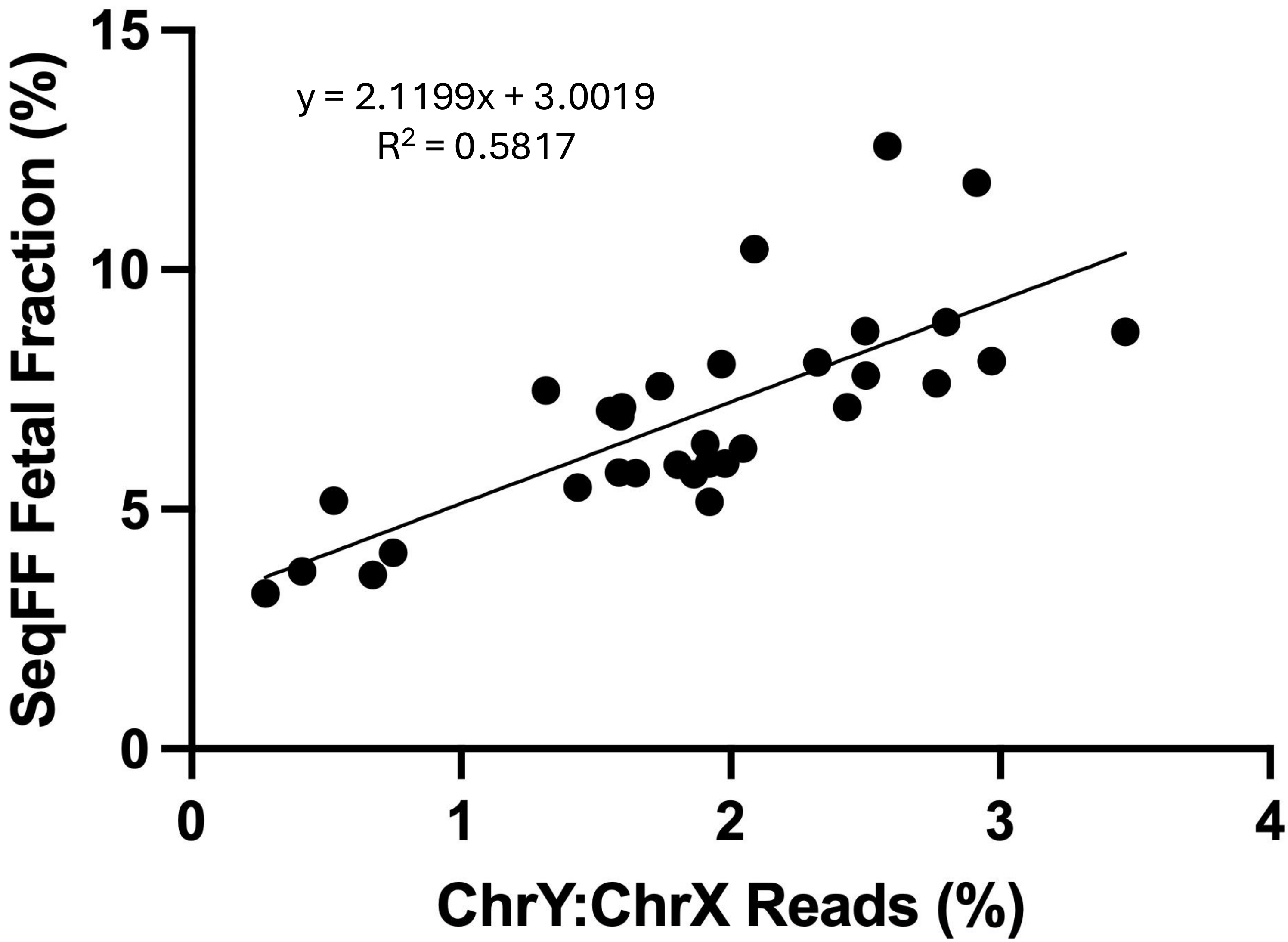

Supplementary Figure 31: Simple linear regression of ChrY:ChrX read fraction vs. SeqFF-estimated fetal fraction in males (n = 33). Equation of the regression provided.

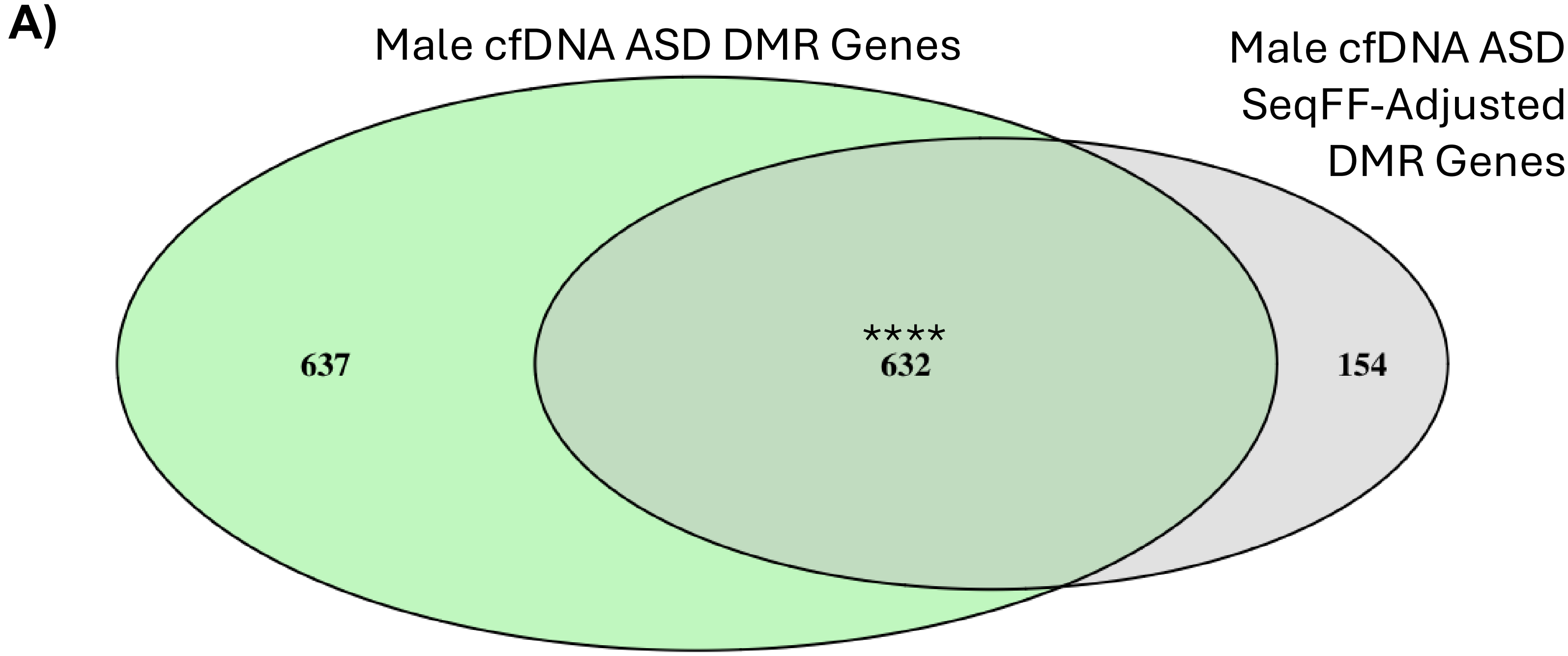

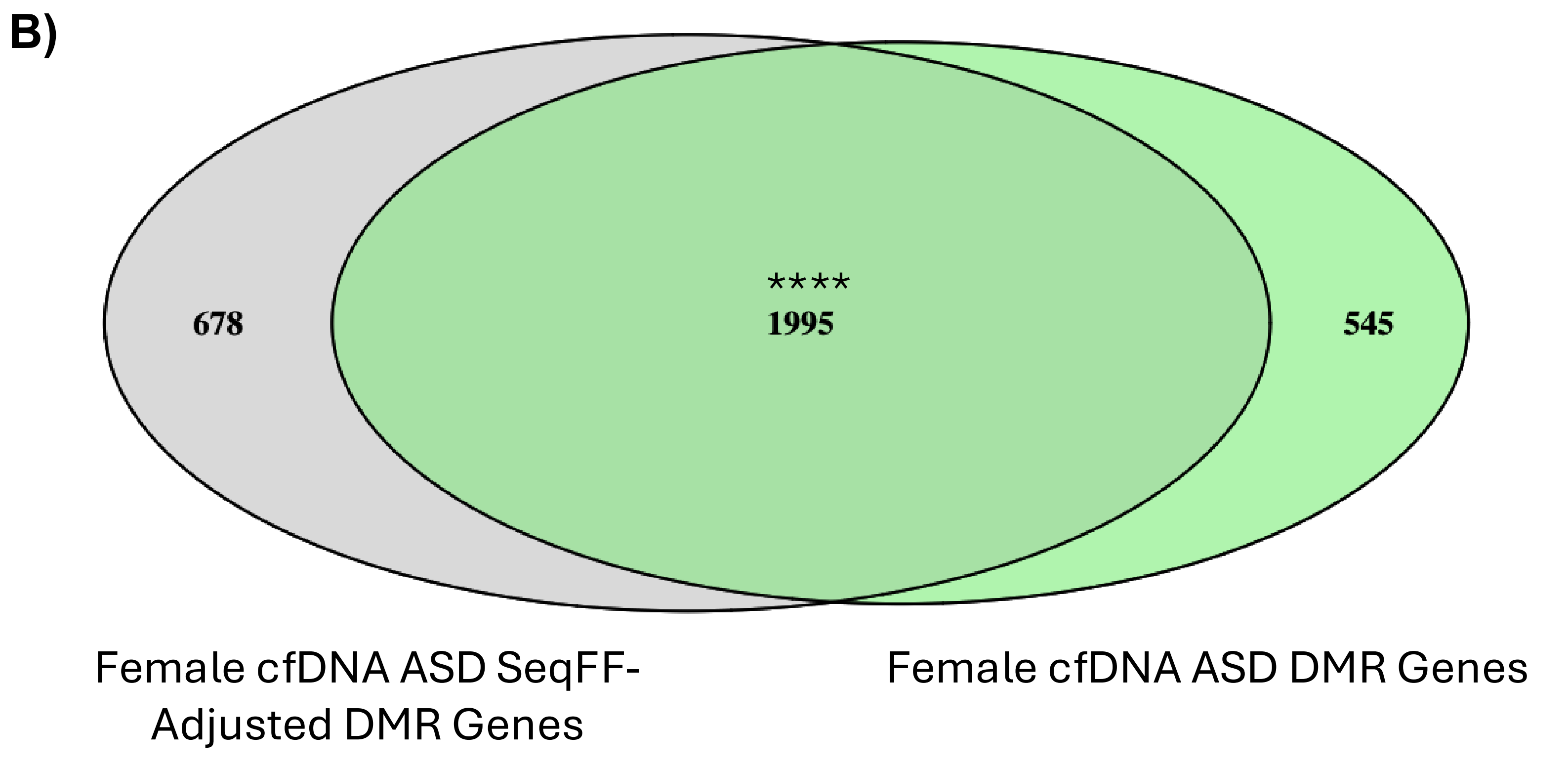

Supplementary Figure 32: Venn diagrams showing gene-level overlap of the genes conserved between reported cfDNA DMRs and cfDNA DMRs generated with adjustment for SeqFF-estimated fetal fraction. Significance of gene lists overlaps relative to the universe of background regions denoted with asterisks (****). Significant overlaps observed for **A)** Male ASD DMRs (p = 0, 632 intersecting genes) and **B)** Female ASD DMRs (p = 0, 1995 intersecting genes).

A)

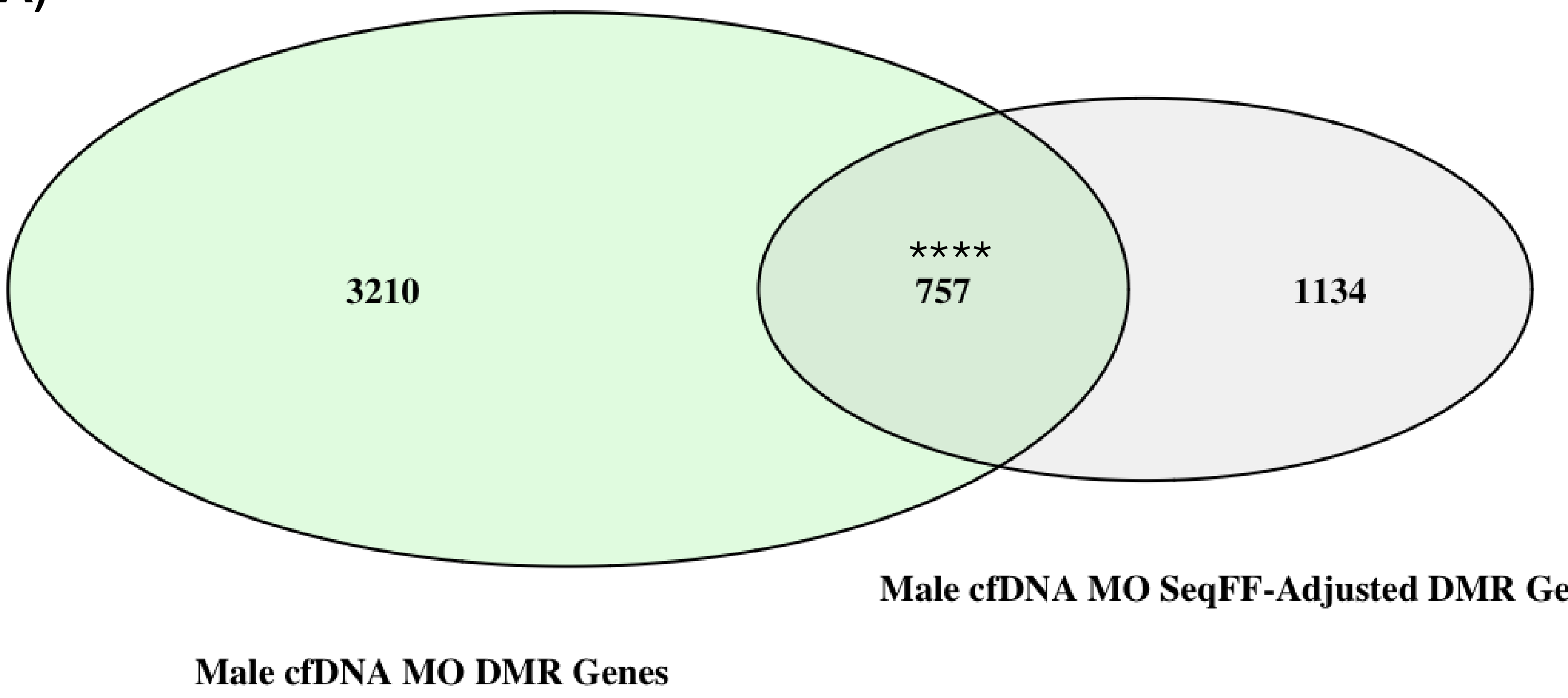

B)

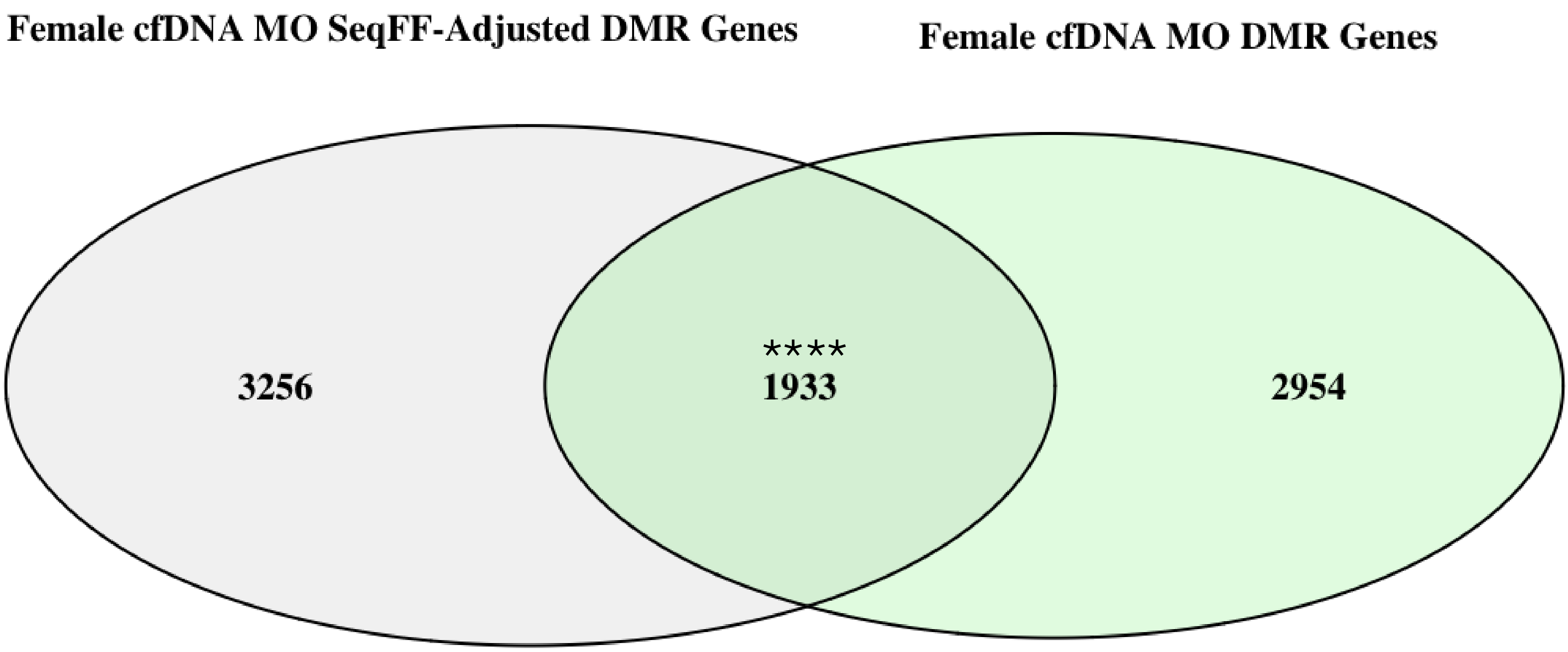

Supplementary Figure 33: Venn diagrams showing gene-level overlap of the genes conserved between reported cfDNA DMRs and cfDNA DMRs generated with adjustment for SeqFF-estimated fetal fraction. Significance of gene lists overlaps relative to the universe of background regions denoted with asterisks (****). Significant overlaps observed for **A)** Male MO DMRs (p = 5.8e-75, 757 intersecting genes), and **B)** Female MO DMRs (p = 4.8e-125, 1933 intersecting genes).

A)

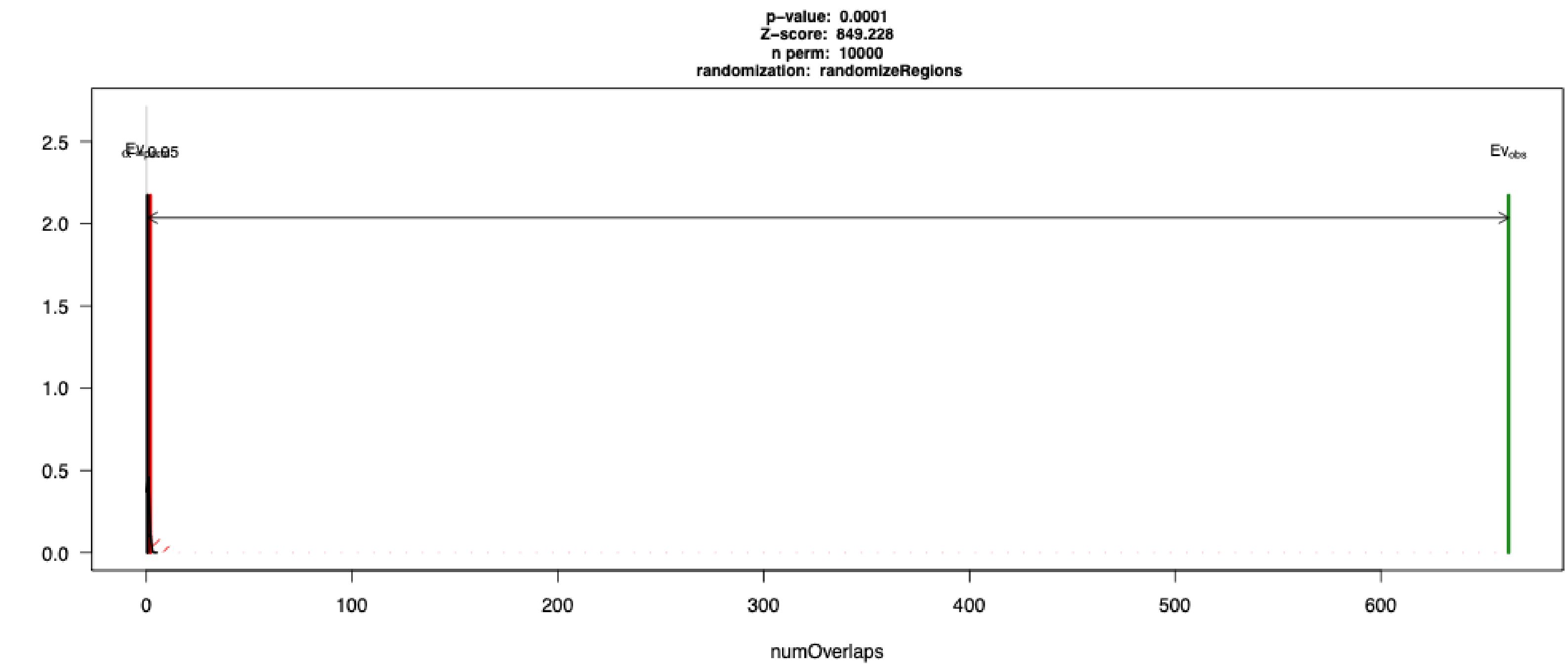

B)

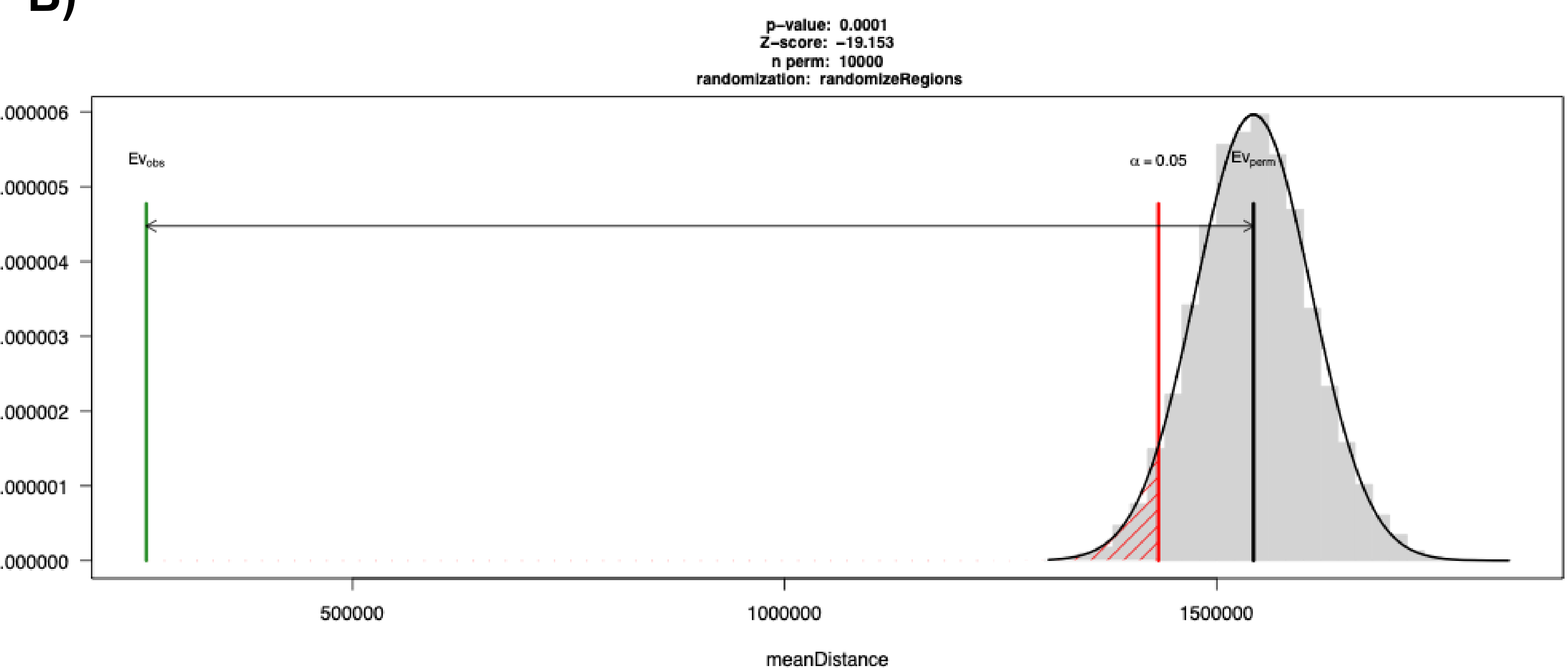

Supplementary Figure 34: Plots of the significance of region level overlap between Male CfDNA ASD DMRs and Male cfDNA ASD SeqFF-adjusted DMRs as determined by permutations testing with respect to **A)** the number of overlaps between the two DMR sets and **B)** the mean distance between the DMRs in those two sets. Y-axis is probability density on both plots.

A)

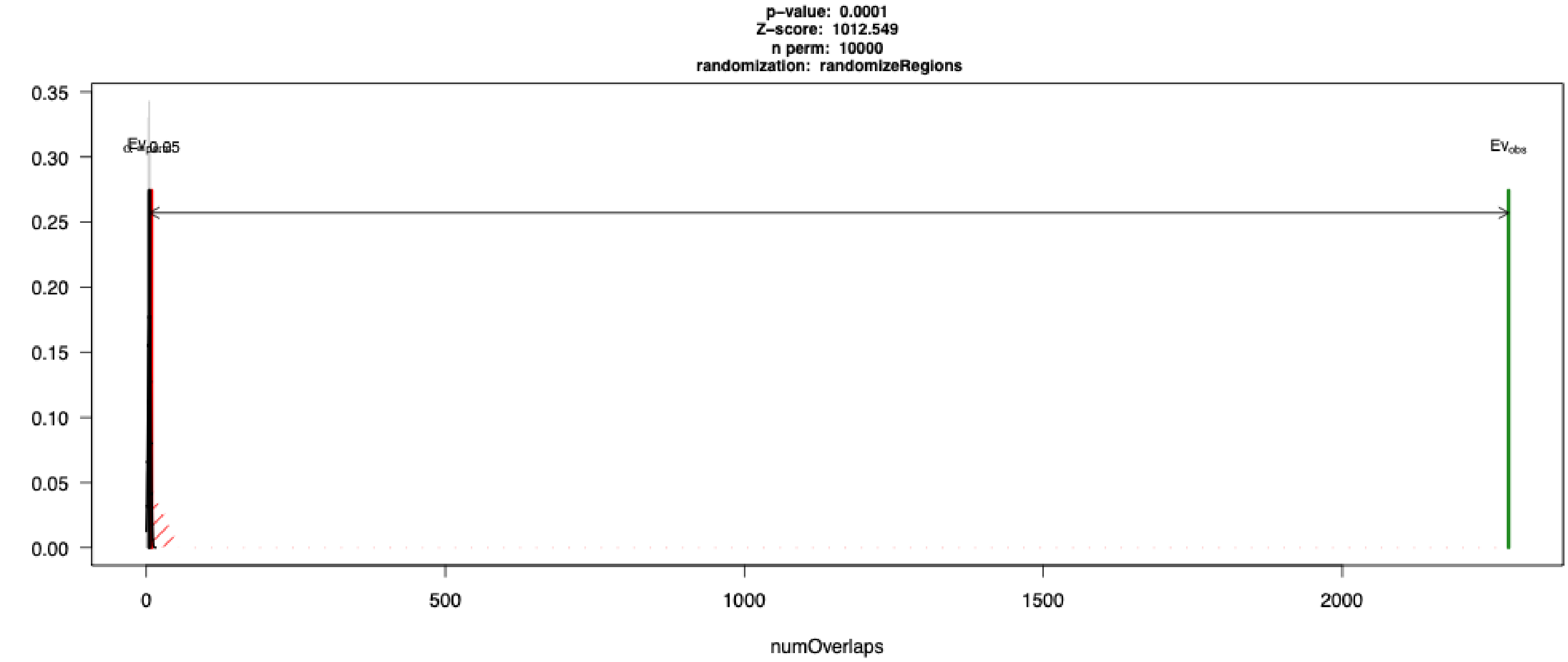

B)

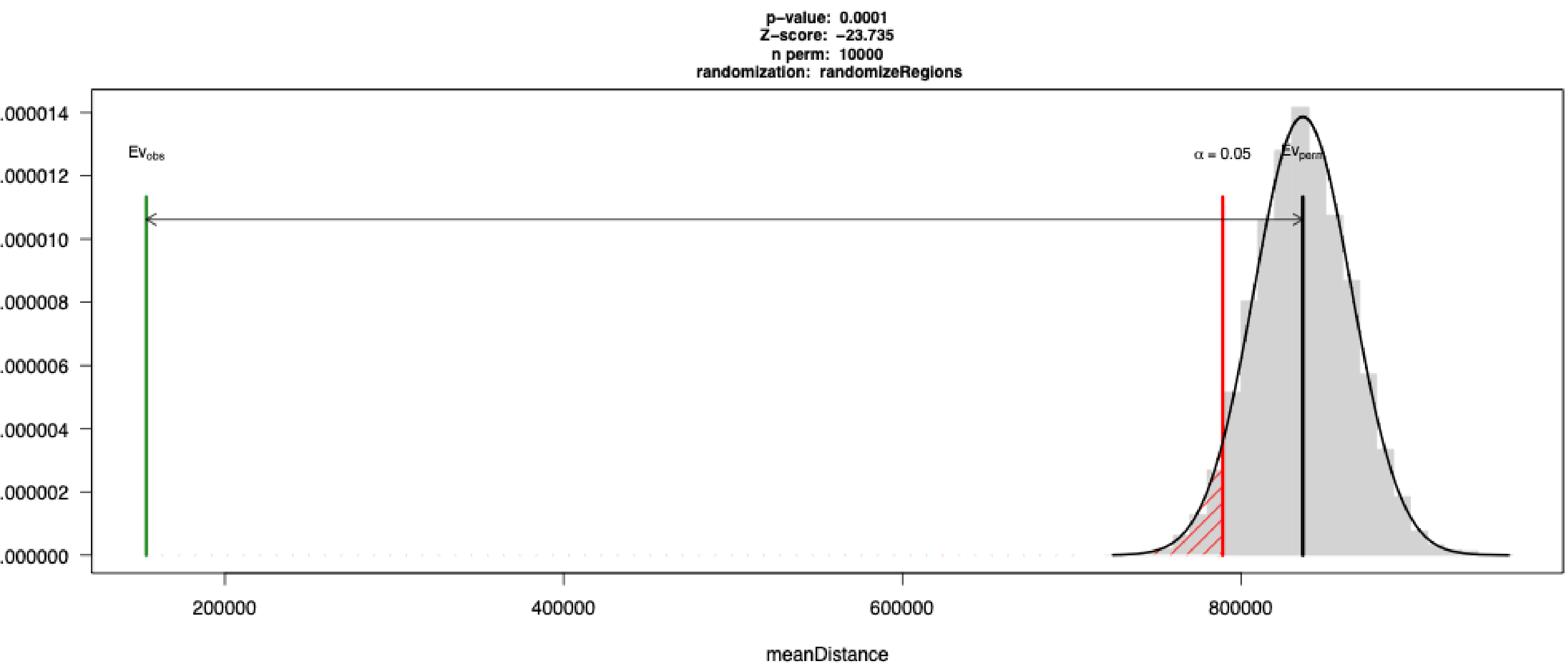

Supplementary Figure 35: Plots of the significance of region level overlap between Female CfDNA ASD DMRs and Female cfDNA ASD SeqFF-adjusted DMRs as determined by permutations testing with respect to **A)** the number of overlaps between the two DMR sets and **B)** the mean distance between the DMRs in those two sets. Y-axis is probability density on both plots.

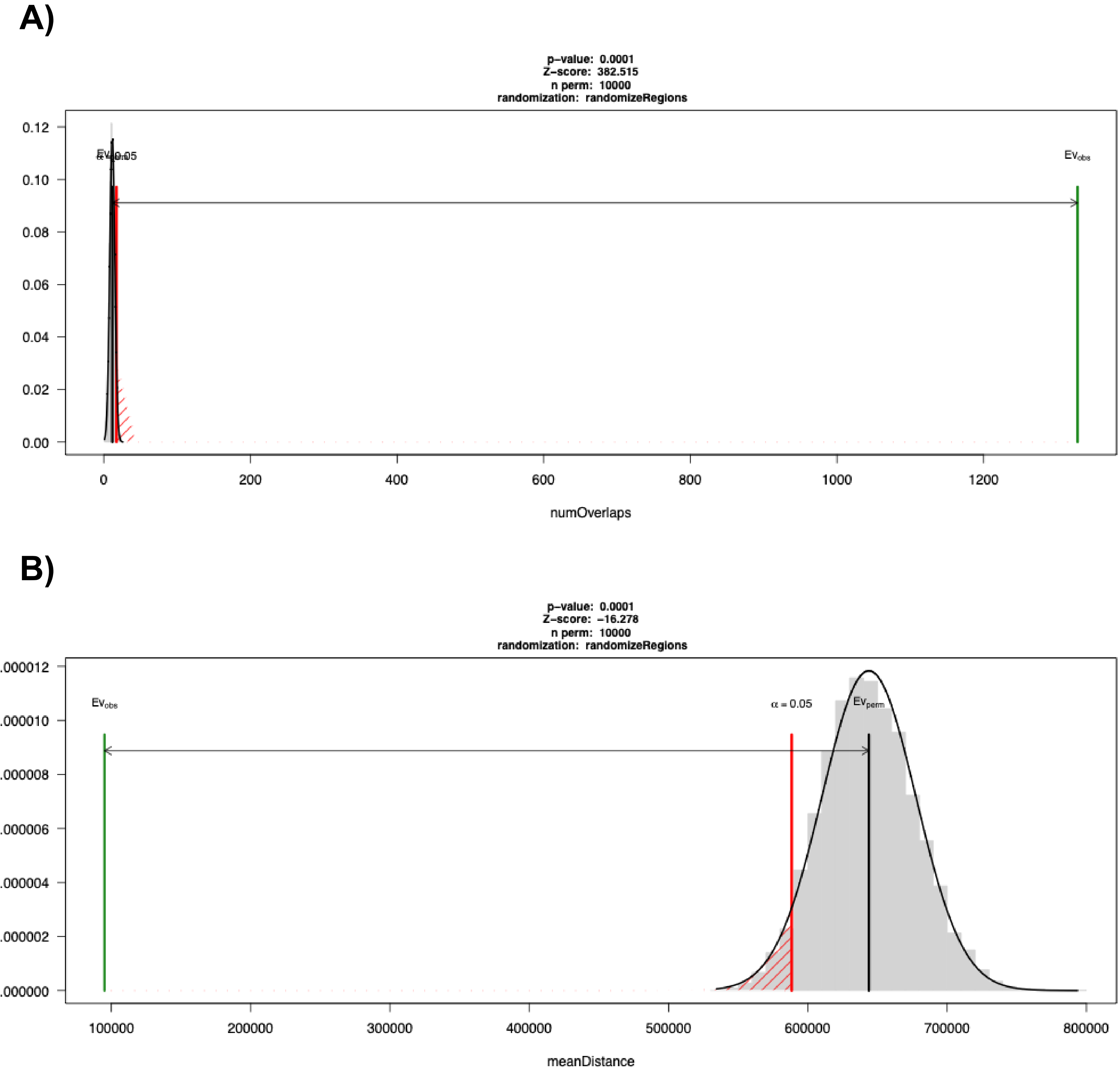

Supplementary Figure 36: Plots of the significance of region level overlap between Male CfDNA MO DMRs and Male cfDNA MO SeqFF-adjusted DMRs as determined by permutations testing with respect to **A)** the number of overlaps between the two DMR sets and **B)** the mean distance between the DMRs in those two sets. Y-axis is probability density on both plots.

A)

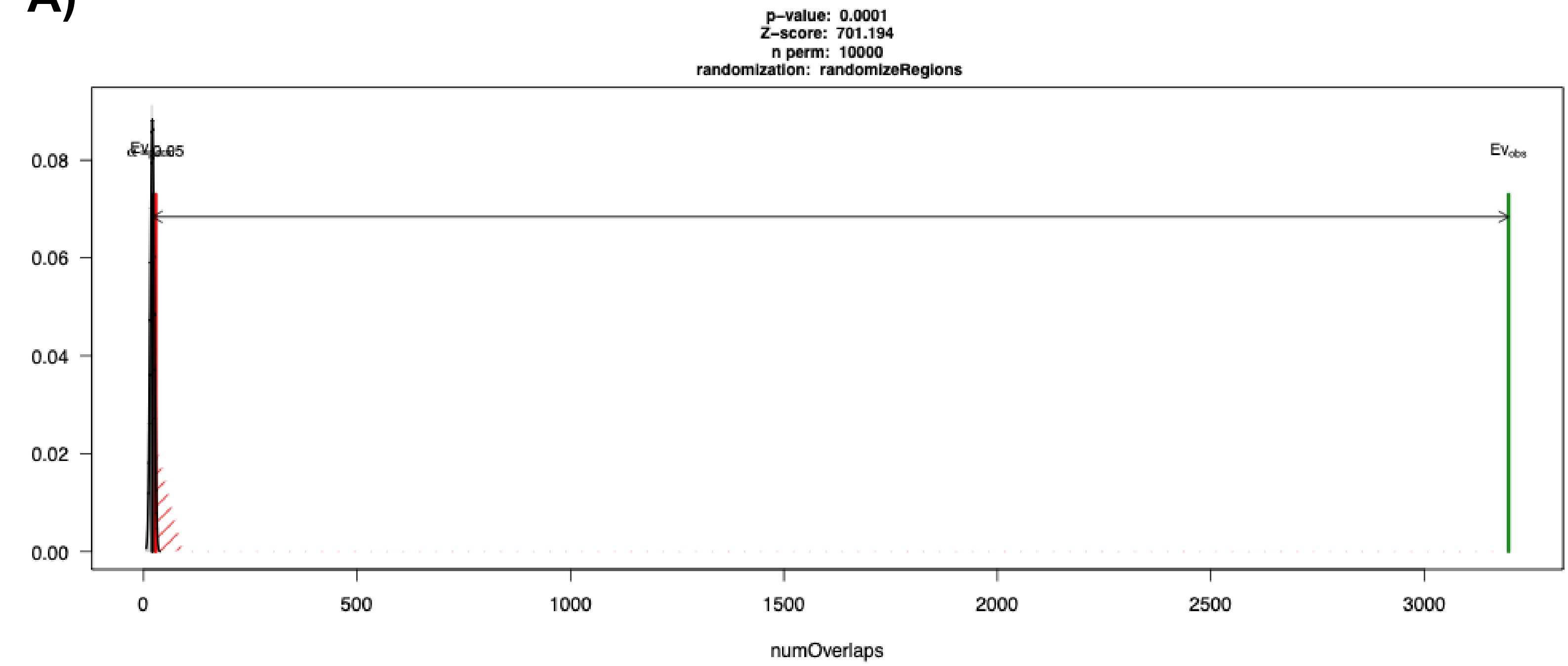

B)

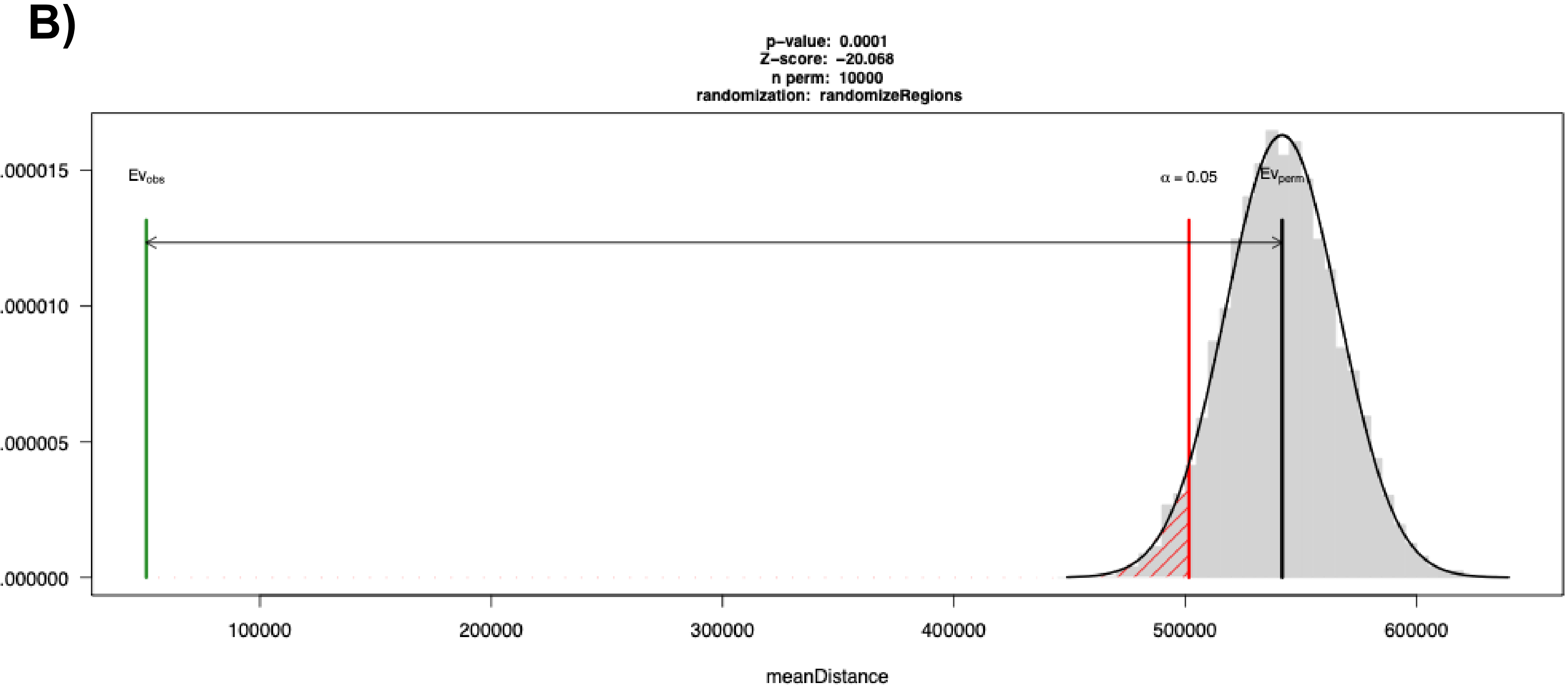

Supplementary Figure 37: Plots of the significance of region level overlap between Female CfDNA MO DMRs and Female cfDNA MO SeqFF-adjusted DMRs as determined by permutations testing with respect to **A)** the number of overlaps between the two DMR sets and **B)** the mean distance between the DMRs in those two sets. Y-axis is probability density on both plots.

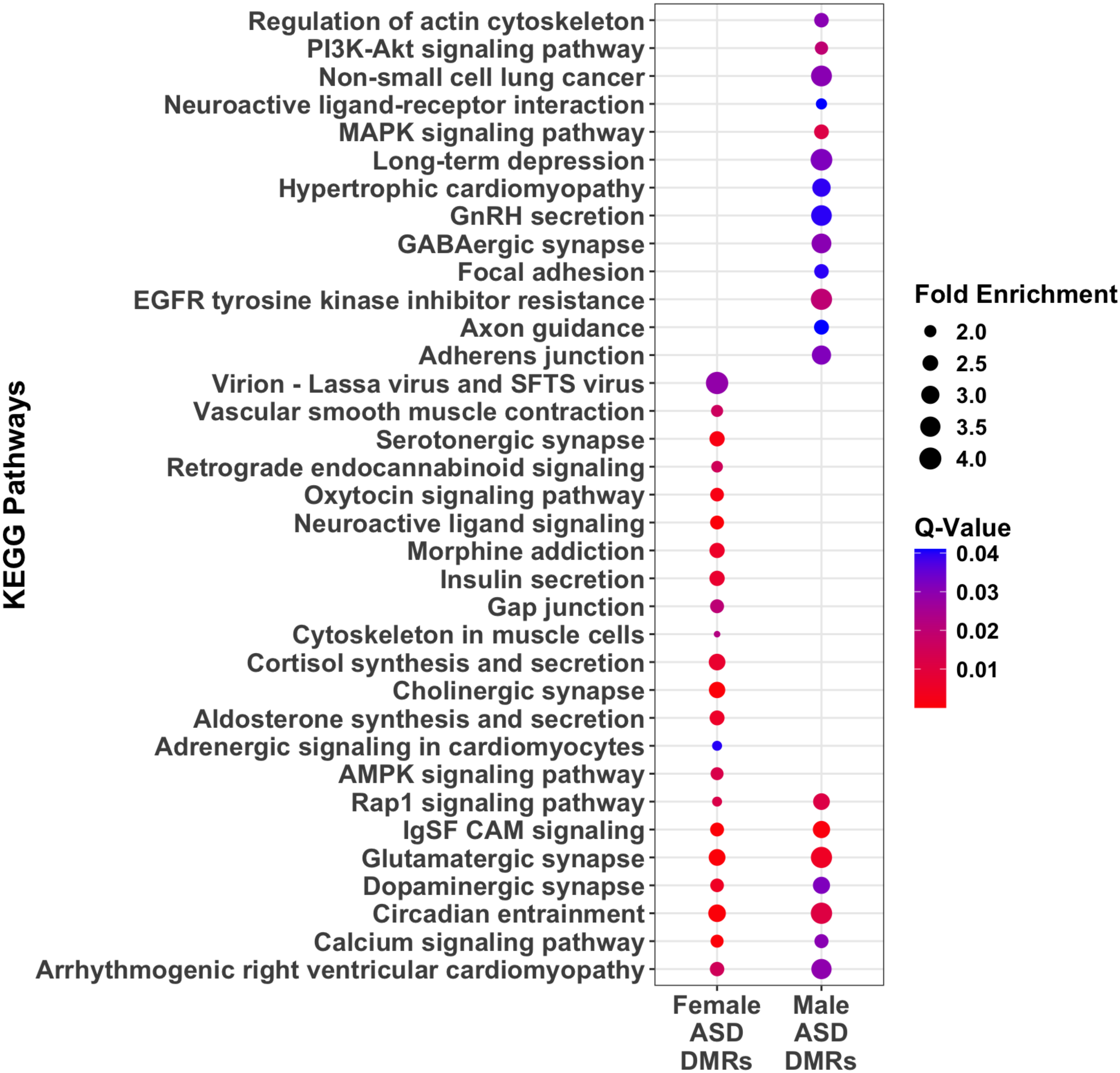

Supplementary Figure 38: All significant KEGG pathway enrichments for male (n = 849) and female (n = 3,283) cfDNA ASD SeqFF-adjusted DMRs. Seven pathways were significantly enriched in both males and females, clustering around synapse development and cell signaling. Four of the seven pathways significantly enriched in both sexes were also significantly enriched in both sexes of the original, non-SeqFF-adjusted DMRs.

A)

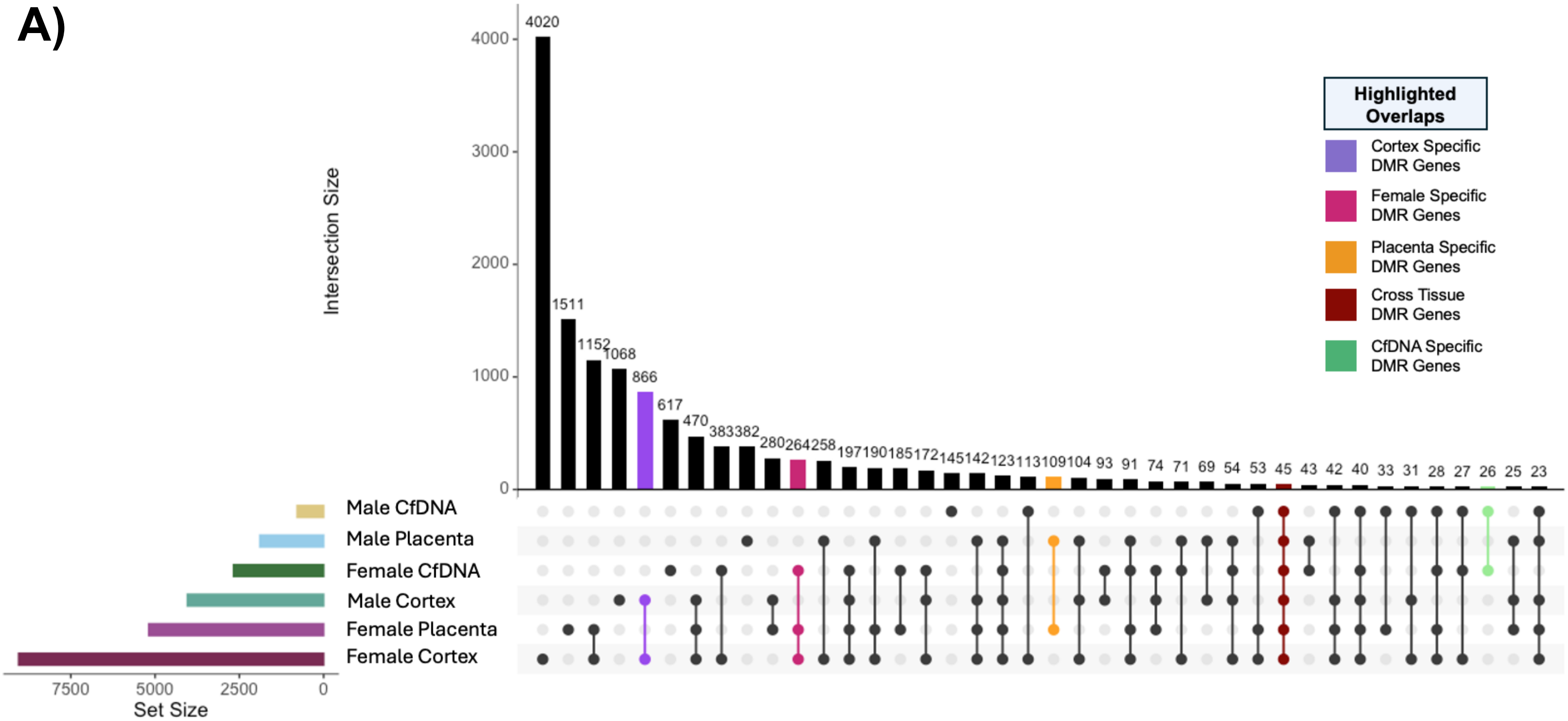

B)

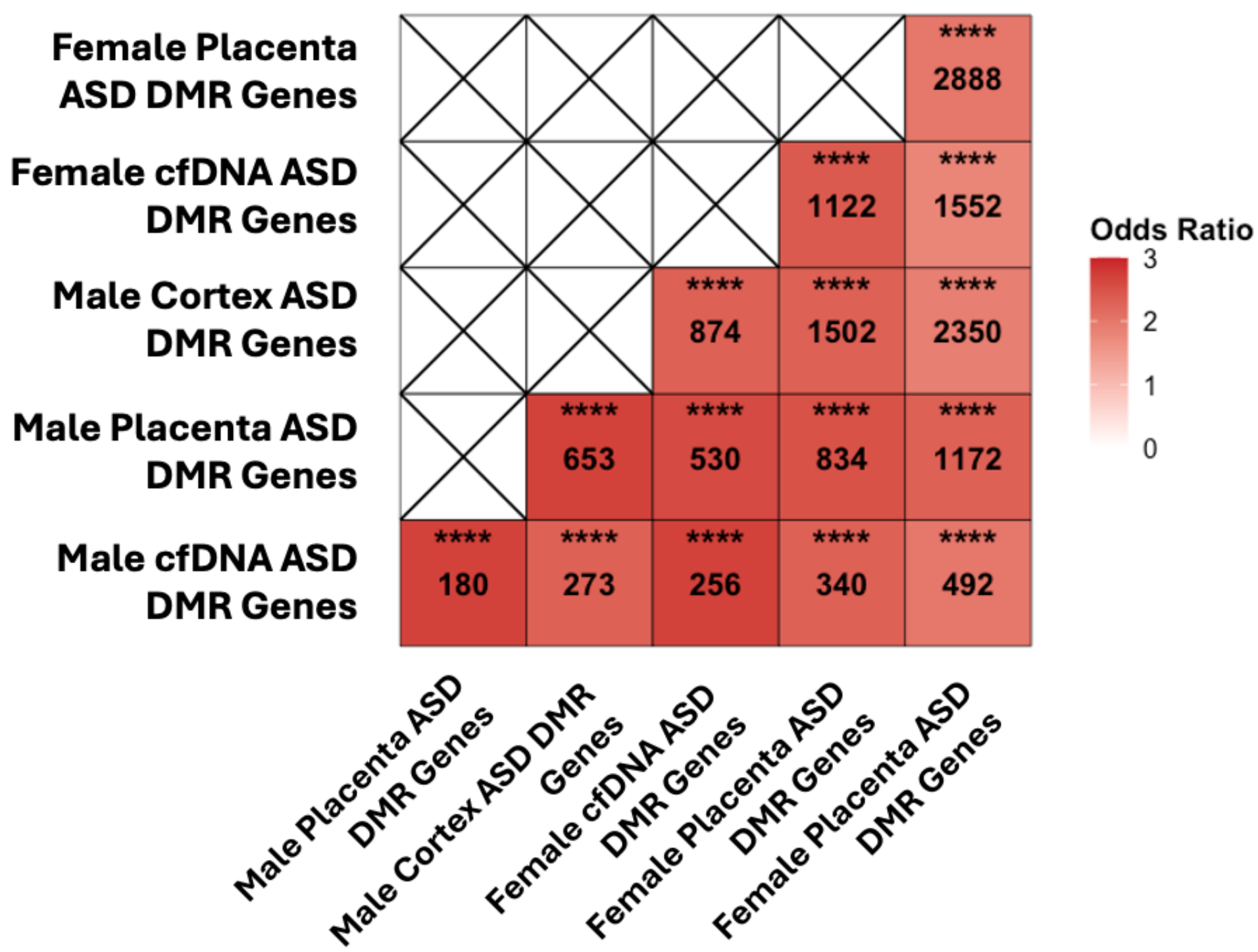

Supplementary Figure 39: A) All gene level overlaps between SeqFF-adjusted cfDNA ASD DMRs, placenta DMRs, and cortex DMRs. **B)** Significance of each the overlap between all possible combinations of DMR gene lists, including the SeqFF-adjusted cfDNA ASD DMRs in males and females. Significance determined by Fisher's Exact Test. The numbers shown on each tile correspond to the number of genes overlapping the two gene lists in question. Asterisks denote p-value as follows: **** $p < 0.0001$

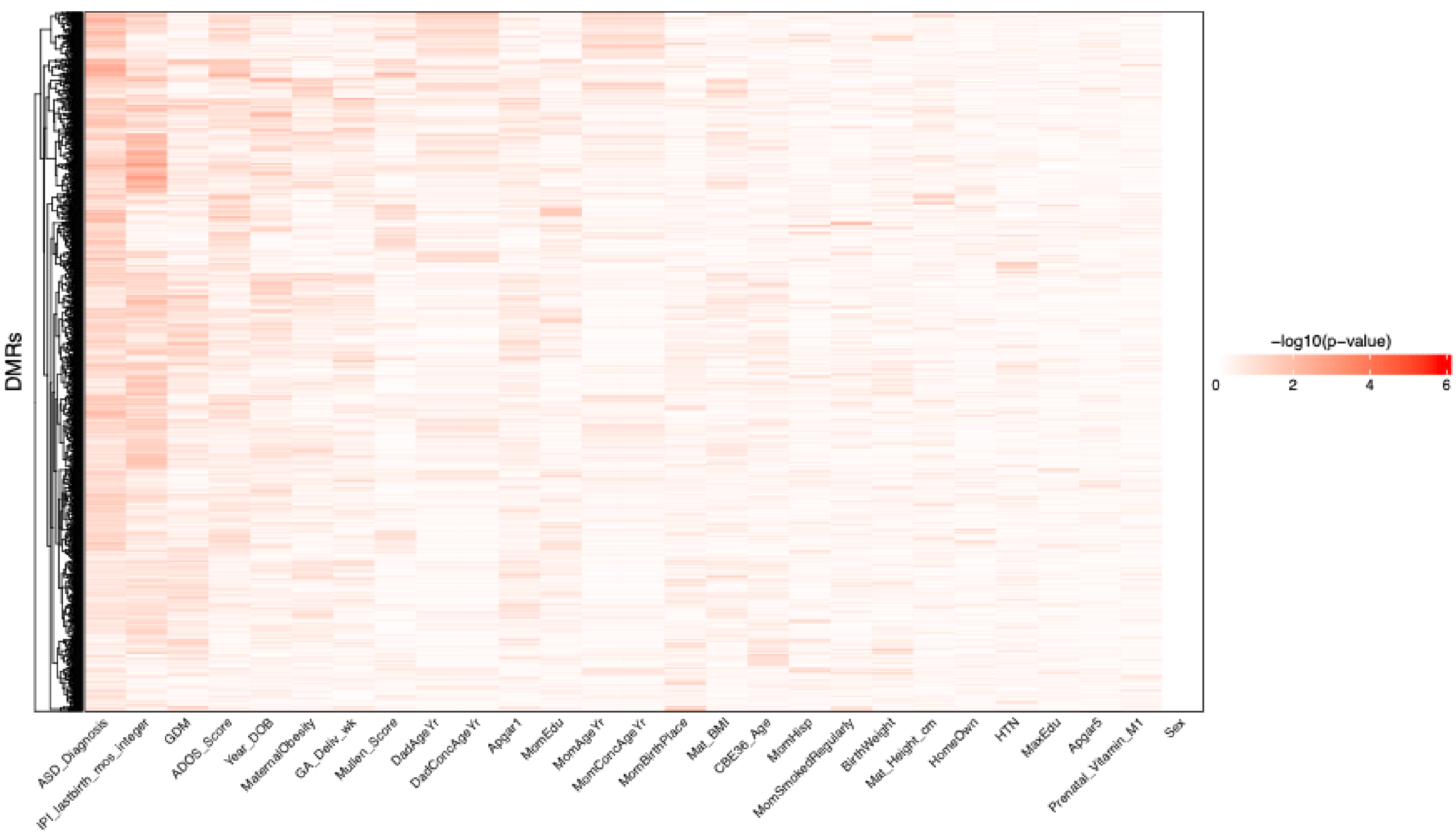

Supplementary Figure 40: Heatmap of significance of association between each male (n = 1,436) cfDNA ASD DMR (y-axis) and each sample covariate (x-axis). Significance determined by t-test of the regression coefficient and quantified as a permutation p-value. Sample covariates ordered on the x-axis by total number of significant (p-value < 0.05) associations with the DMRs, descending from left to right.

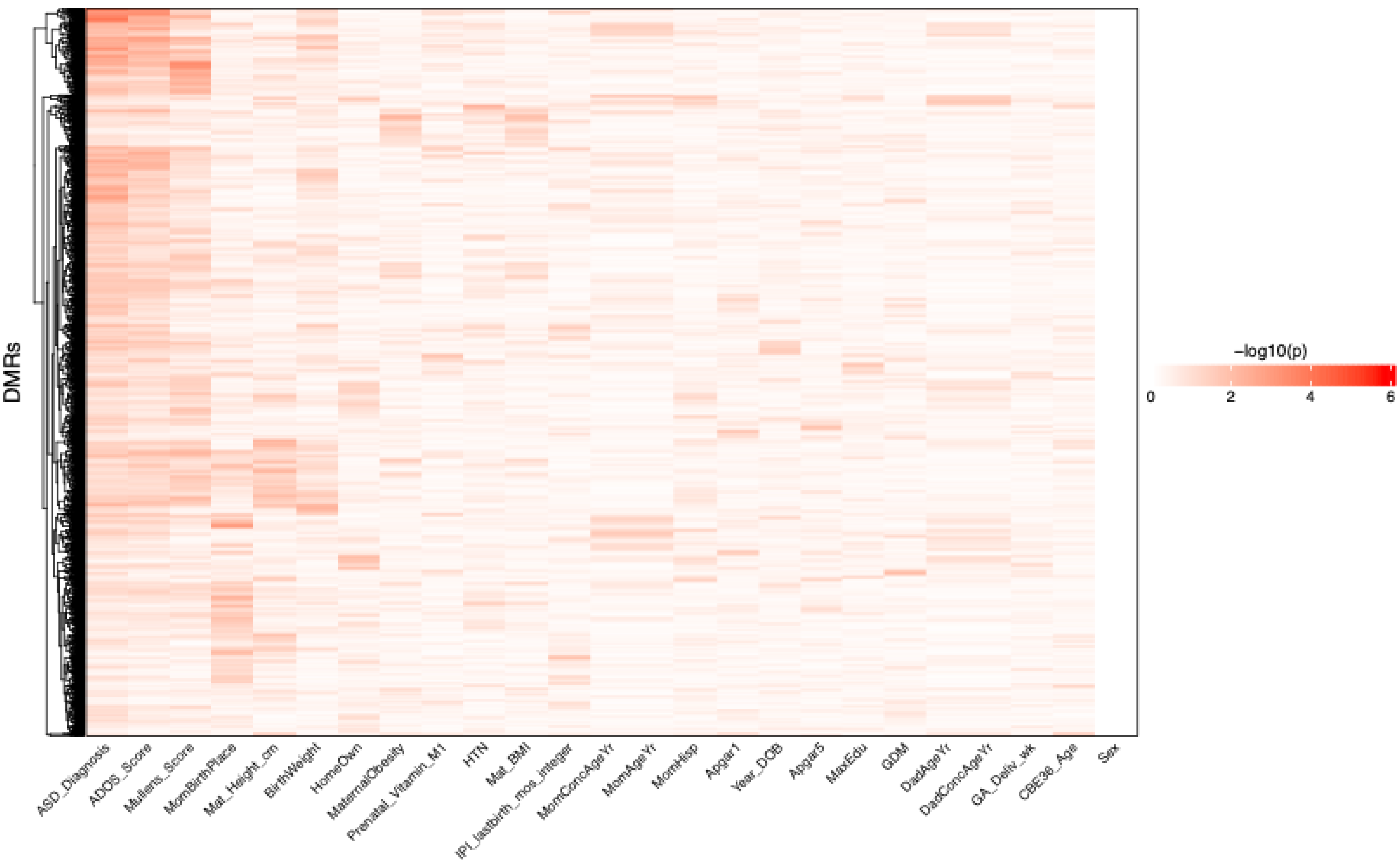

Supplementary Figure 41: Heatmap of significance of association between each female (n = 3,113) cfDNA ASD DMR (y-axis) and each sample covariate (x-axis). Significance determined by t-test of the regression coefficient and quantified as a permutation p-value. Sample covariates ordered on the x-axis by total number of significant (p-value < 0.05) associations with the DMRs, descending from left to right.

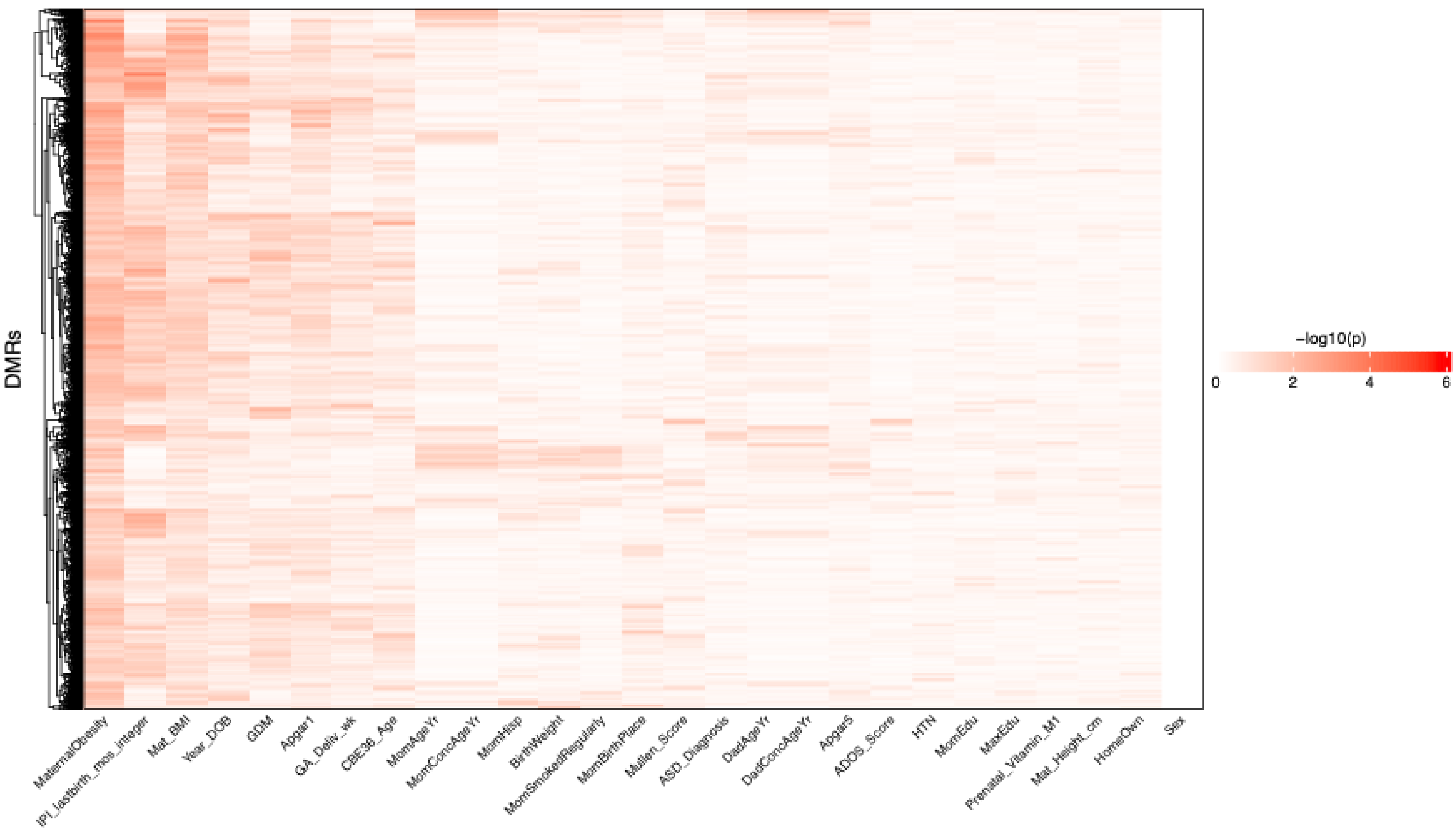

Supplementary Figure 42: Heatmap of significance of association between each male (n = 6,677) cfDNA MO DMR (y-axis) and each sample covariate (x-axis). Significance determined by t-test of the regression coefficient and quantified as a permutation p-value. Sample covariates ordered on the x-axis by total number of significant (p-value < 0.05) associations with the DMRs, descending from left to right.

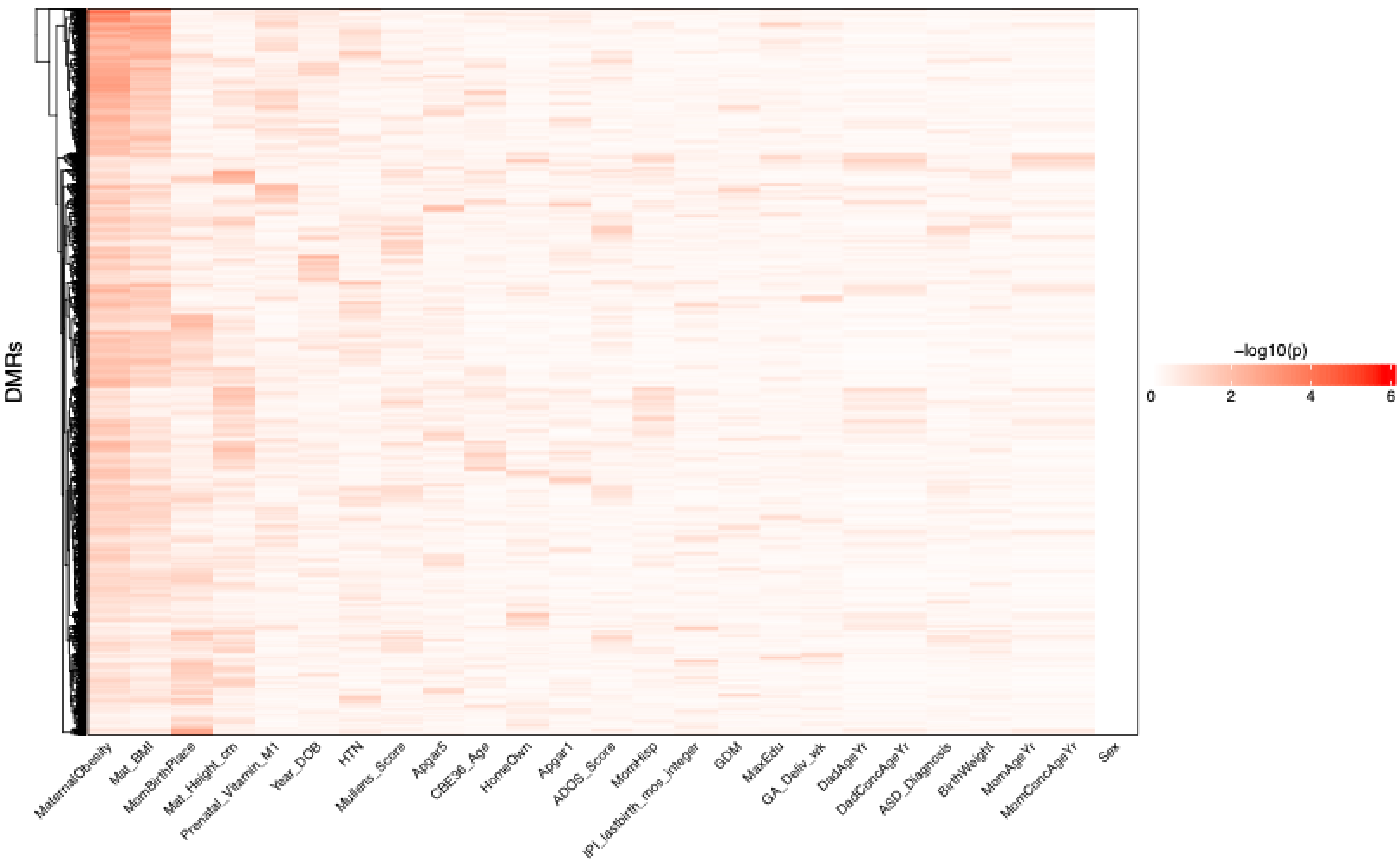

Supplementary Figure 43: Heatmap of significance of association between each female (n = 7,748) cfDNA MO DMR (y-axis) and each sample covariate (x-axis). Significance determined by t-test of the regression coefficient and quantified as a permutation p-value. Sample covariates ordered on the x-axis by total number of significant (p-value < 0.05) associations with the DMRs, descending from left to right.
