# Supplementary material for: Prenatal cell-free DNA methylome detects association with autism and maternal obesity
Source: Commun Biol. 2026 Jun 22;9:1319. doi: 10.1038/s42003-026-10500-9 (PMC13650157; doi:10.1038/s42003-026-10500-9)
Supplement: Supplementary file 2 [file 42003_2026_10500_MOESM2_ESM.pdf]

Description of Additional Supplementary Files

File name: Supplementary Data 1

Description: MARBLES participants subject characteristics

File name: Supplementary Data 2

Description: MARBLES participants characteristics data dictionary

File name: Supplementary Data 3

Description: Correlation between all subject characteristics

File name: Supplementary Data 4

Description: P-values of correlations between all subject characteristics

File name: Supplementary Data 5

Description: Fetal male pregnancies cfDNA ASD DMRs and background regions

File name: Supplementary Data 6

Description: Fetal female pregnancies cfDNA ASD DMRs and background regions

File name: Supplementary Data 7

Description: Summary table of DMR methylation directionality and global CpG methylation

File name: Supplementary Data 8

Description: Sex-combined cfDNA ASD DMRs and background regions

File name: Supplementary Data 9

Description: Full KEGG enrichment report for Male cfDNA ASD DMRs

File name: Supplementary Data 10

Description: Full KEGG enrichment report for Female cfDNA ASD DMRs

File name: Supplementary Data 11

Description: CfDNA DMR genes SFARI enrichment by confidence level

File name: Supplementary Data 12

Description: List of 60 genes found in both male and female cfDNA ASD DMR lists and the SFARI gene list

File name: Supplementary Data 13

Description: ChromHMM enrichment analysis report for Male cfDNA ASD DMRs

File name: Supplementary Data 14

Description: ChromHMM enrichment analysis report for Female cfDNA ASD DMRs

File name: Supplementary Data 15

Description: Fetal male pregnancies placenta ASD DMRs and background regions

File name: Supplementary Data 16

Description: Fetal female pregnancies placenta ASD DMRs and background regions

File name: Supplementary Data 17

Description: Male post-mortem cortex ASD DMRs and background regions

File name: Supplementary Data 18

Description: Female post-mortem cortex ASD DMRs and background regions

File name: Supplementary Data 19

Description: List of cross tissue genes found in all six ASD DMR sets

File name: Supplementary Data 20

Description: Full KEGG enrichment report for cross-tissue ASD DMR genes

File name: Supplementary Data 21

Description: List of genes specific to female ASD DMRs

File name: Supplementary Data 22

Description: Fetal male pregnancies cfDNA MO DMRS

File name: Supplementary Data 23

Description: Fetal female pregnancies cfDNA MO DMRS

File name: Supplementary Data 24

Description: List of 210 genes found in all four cfDNA DMR gene lists

File name: Supplementary Data 25

Description: Table detailing the number of cfDNA samples across intersecting ASD and MO classifications

File name: Supplementary Data 26

Description: Fetal male pregnancies cfDNA ASD DMRs (with fetal fraction adjustment) and background regions

File name: Supplementary Data 27

Description: Fetal female pregnancies cfDNA ASD DMRs (with fetal fraction adjustment) and background regions

File name: Supplementary Data 28

Description: Fetal male pregnancies cfDNA MO DMRs (with fetal fraction adjustment) and background regions

File name: Supplementary Data 29

Description: Fetal female pregnancies cfDNA MO DMRs (with fetal fraction adjustment) and background regions

File name: Supplementary Data 30

Description: Tables describing the placenta and cortex ASD DMRs that overlap with epigenetic clocks

File name: Supplementary Data 31

Description: DMR level comparison of CpG coverage between ASD and Control groups in male placenta ASD DMR analysis

File name: Supplementary Data 32

Description: DMR level comparison of CpG coverage between ASD and Control groups in male placenta ASD DMR analysis

File name: Supplementary Data 33

Description: Per-chromosome overrepresentation analysis results for male Placenta ASD DMRs with nominally significant (unadjusted $p < 0.05$) coverage differences between ASD and Control groups against a background of all reported DMRs

File name: Supplementary Data 34

Description: Per-chromosome overrepresentation analysis results for female Placenta ASD DMRs with nominally significant (unadjusted $p < 0.05$) coverage differences between ASD and Control groups against a background of all reported DMRs

File name: Supplementary Data 35

Description: Full KEGG enrichment report for Male MO DMR genes

File name: Supplementary Data 36

Description: Full KEGG enrichment report for Female MO DMR genes

File name: Supplementary Data 37

Description: Full KEGG enrichment report for the 210 genes found in all four cfDNA DMR gene lists
