# Supplementary material for: Prenatal cell-free DNA methylome detects association with autism and maternal obesity
Source: Commun Biol. 2026 Jun 22;9:1319. doi: 10.1038/s42003-026-10500-9 (PMC13650157; doi:10.1038/s42003-026-10500-9)
Supplement: Supplementary file 3 [file 42003_2026_10500_MOESM4_ESM.pdf]

nature portfolio

Corresponding author(s): Janine M. LaSale

Last updated by author(s): 05/07/2026

Reporting Summary

Nature Portfolio wishes to improve the reproducibility of the work that we publish. This form provides structure for consistency and transparency in reporting. For further information on Nature Portfolio policies, see our Editorial Policies and the Editorial Policy Checklist.

Statistics

For all statistical analyses, confirm that the following items are present in the figure legend, table legend, main text, or Methods section.

n/a	Confirmed	
☐	☒	The exact sample size (*n*) for each experimental group/condition, given as a discrete number and unit of measurement
☐	☒	A statement on whether measurements were taken from distinct samples or whether the same sample was measured repeatedly
☐	☒	The statistical test(s) used AND whether they are one- or two-sided<br>*Only common tests should be described solely by name; describe more complex techniques in the Methods section.*
☐	☒	A description of all covariates tested
☐	☒	A description of any assumptions or corrections, such as tests of normality and adjustment for multiple comparisons
☐	☒	A full description of the statistical parameters including central tendency (e.g. means) or other basic estimates (e.g. regression coefficient) AND variation (e.g. standard deviation) or associated estimates of uncertainty (e.g. confidence intervals)
☐	☒	For null hypothesis testing, the test statistic (e.g. *F*, *t*, *r*) with confidence intervals, effect sizes, degrees of freedom and *P* value noted<br>*Give P values as exact values whenever suitable.*
☒	☐	For Bayesian analysis, information on the choice of priors and Markov chain Monte Carlo settings
☒	☐	For hierarchical and complex designs, identification of the appropriate level for tests and full reporting of outcomes
☒	☐	Estimates of effect sizes (e.g. Cohen's *d*, Pearson's *r*), indicating how they were calculated

Our web collection on statistics for biologists contains articles on many of the points above.

Software and code

Policy information about availability of computer code

Data collection	Microsoft Excel version 16.93
Data analysis	R version 4.5.0 and 4.2.3<br>DMRichR v1.7.1, Epigenerator (https://github.com/vhaghani26/epigenerator)<br>Additional code that support these findings can be found on GitHub at the following repository: https://github.com/lwilliams27/CfDNA_Methylome_AutismAndMaternalObesity .

For manuscripts utilizing custom algorithms or software that are central to the research but not yet described in published literature, software must be made available to editors and reviewers. We strongly encourage code deposition in a community repository (e.g. GitHub). See the Nature Portfolio guidelines for submitting code & software for further information.

Data

Policy information about availability of data

All manuscripts must include a data availability statement. This statement should provide the following information, where applicable:

- Accession codes, unique identifiers, or web links for publicly available datasets
- A description of any restrictions on data availability
- For clinical datasets or third party data, please ensure that the statement adheres to our policy

The sequencing data generated by this study from all subjects that have provided consent (n=48) have been deposited in the GEO database under accession code

GSE307129. Previously published WGBS data for placenta and cortex can be accessed in the GEO database under accession codes GSE178206 (placenta) and GSE119981 (cortex).
Source data are provided with this paper.

Research involving human participants, their data, or biological material

Policy information about studies with human participants or human data. See also policy information about sex, gender (identity/presentation), and sexual orientation and race, ethnicity and racism.

Reporting on sex and gender	DMR calls for ASD and MO were reported in sex-stratified cohorts due to the substantive differences observed between sexed cohorts with respect to cfDNA methylation. Sex was self-reported by MARBLES participants, though confirmed by the presence/absence of Y chromosome reads in the cfDNA data.
Reporting on race, ethnicity, or other socially relevant groupings	This study was not designed to assess the potential impact of race and ethnicity on epigenetic associations for ASD or MO. For purposes of completion and transparency, statistics of correlation between ethnicity and numerous other available participant covariates are included in Supplementary Tables 3 and 4. Ethnicity was coded as binary depending on a mother's response to questionnaire during MARBLES enrollment. As described in the data dictionary, a value of 0 indicates a mother selected "Not Hispanic" and a value of 1 indicates a mother selected "Hispanic". This classification framework was provided by researchers, but reflects the framework supported by the California Census Office. Individual-level ethnicity data is not reported here due to its nature as potentially identifying.
Population characteristics	Full reported population characteristics can be found in Supplementary Table 1 and 2. To protect the anonymity of the study participants, subject-level covariates that are potentially identifying are omitted wherever possible.
Recruitment	As previously described (https://ehp.niehs.nih.gov/doi/10.1289/EHP535), MARBLES recruits through the California Department of Developmental Services (DDS). This may result in the underrepresentation of milder cases of ASD, as DDS guidelines implemented in 2003 required individuals receiving DDS services to have at least three significant functional limitations.
Ethics oversight	The MARBLES study protocol was approved by the Institutional Review Boards (IRB) for the State of California and the University of California, Davis.

Note that full information on the approval of the study protocol must also be provided in the manuscript.

Field-specific reporting

Please select the one below that is the best fit for your research. If you are not sure, read the appropriate sections before making your selection.

☒ Life sciences ☐ Behavioural & social sciences ☐ Ecological, evolutionary & environmental sciences

For a reference copy of the document with all sections, see nature.com/documents/nr-reporting-summary-flat.pdf

Life sciences study design

All studies must disclose on these points even when the disclosure is negative.

Sample size	Participants from the MARBLES cohort with available plasma and pregnancy outcome data (n=51) were stratified by sex for analysis of ASD and MO DMRs. The ASD DMR analysis compared non-ASD control males (n=19) to ASD males (n=14) and non-ASD control females (n=8) to ASD females (n=10). The MO DMR analysis compared non-MO control males (n=24) to MO males (n=9) and non-MO control females (n=9) to MO females (n=9). These sample sizes represent the maximum possible sample size for this proof-of-principle study due to the combined limitations of requiring samples with high volumes of precious plasma and the paucity of prospective pregnancy studies that collect and share these resources. Our sample sizes are typical of other published works on findings from similarly precious tissues (PMID: 36130949, 33795684, PMID: 33602921).
Data exclusions	No data were excluded from the analyses.
Replication	Each DMR analysis had multiple biological replicates within each sex stratified group.
Randomization	Participants were sorted into control and MO groups by maternal body mass index at pre-pregnancy. Participants were sorted into control and ASD groups by clinical assessment results at 3 years of age.
Blinding	Investigators were blinded to group allocation during data collection and analysis when possible.

Reporting for specific materials, systems and methods

We require information from authors about some types of materials, experimental systems and methods used in many studies. Here, indicate whether each material, system or method listed is relevant to your study. If you are not sure if a list item applies to your research, read the appropriate section before selecting a response.

Materials & experimental systems

n/a	Involved in the study
☒	☐ Antibodies
☒	☐ Eukaryotic cell lines
☒	☐ Palaeontology and archaeology
☒	☐ Animals and other organisms
☒	☐ Clinical data
☒	☐ Dual use research of concern
☒	☐ Plants

Methods

n/a	Involved in the study
☒	☐ ChIP-seq
☒	☐ Flow cytometry
☒	☐ MRI-based neuroimaging

Plants

Seed stocks

Report on the source of all seed stocks or other plant material used. If applicable, state the seed stock centre and catalogue number. If plant specimens were collected from the field, describe the collection location, date and sampling procedures.

Novel plant genotypes

Describe the methods by which all novel plant genotypes were produced. This includes those generated by transgenic approaches, gene editing, chemical/radiation-based mutagenesis and hybridization. For transgenic lines, describe the transformation method, the number of independent lines analyzed and the generation upon which experiments were performed. For gene-edited lines, describe the editor used, the endogenous sequence targeted for editing, the targeting guide RNA sequence (if applicable) and how the editor was applied.

Authentication

Describe any authentication procedures for each seed stock used or novel genotype generated. Describe any experiments used to assess the effect of a mutation and, where applicable, how potential secondary effects (e.g. second site T-DNA insertions, mosiacism, off-target gene editing) were examined.
