# Supplementary material for: Prenatal cell-free DNA methylome detects association with autism and maternal obesity
Source: Commun Biol. 2026 Jun 22;9:1319. doi: 10.1038/s42003-026-10500-9 (PMC13650157; doi:10.1038/s42003-026-10500-9)
Supplement: Supplementary file 4 [file 42003_2026_10500_MOESM5_ESM.pdf]

nature portfolio

Peer Review File

Prenatal cell-free DNA methylome detects association with autism and maternal obesity

Corresponding Author: Dr Janine LaSalle

This file contains all reviewer reports in order by version, followed by all author rebuttals in order by version.

Version 0:

Reviewer comments:

Reviewer #1

(Remarks to the Author)
The authors performed cfDNA methylation analysis to identify signatures of autism (ASD). The study is important as it can inform minimally invasive prediction of patients at risk. Methylation changes in the placenta and brain samples were intersected with those observed in blood and and some biologically plausible pathways were implicated based on differential methylation patterns. The authors have also shared the data in the Gene Expression Omnibus, providing an important resource to the community. There are however a few aspects that need to be addressed to allow better assessment of the robustness of findings:

1) The authors state “Significant DMRs were defined as those with a permutation p-value of < 0.05”. Assuming hundreds of thousands of DMRs are tested for differential methylation, finding 1-2 thousands of DMRs does not seem unlikely by chance using nominal p-value cut-offs, regardless that permutations were used to calculate the p-values. Are there any significant findings after FDR correction?
2) Related to the question above about not having proving DMR level differences beyond what is expected by chance is the finding of the authors that there were only 377 DMRs when using all pregnancies (n=51), 1,436 ASD related DMRs in male (n=33) and 3,113 in female pregnancies (n=18) fits the idea of randomness because it is easier to get small permutations-based p-values when sample size is low than when sample size is large. Is the magnitude of DMR changes overall higher in female than in males pregnancies?
3) The authors mention that samples were collected at 36-41 weeks, but I did not see a table showing differences in gestational age at sample collection between cases and controls and differences in maternal age. Both factors could affect methylation patterns and affect the results.
4) Supplementary figures 2-4 can be removed because PCA plots based on features preselected because they seem most different between groups will always lead to distinct clusters when starting with a large number of initial features. The authors could start with randomly generated data for 100,000 DMRs and select top 1000 DMR or so which seem most different using a t-test p-values and can see that cluster differences will always emerge.

Reviewer #2

(Remarks to the Author)
In this paper the authors studied the methylation changes in maternal plasma cfDNA between control and pregnancies with babies later diagnosed with ASD. The study is extensive, involving 51 samples and comparisons with placenta/brain data.

However, there is one major flaw in this study, which needs to be addressed. The samples studies in this paper are maternal plasma cfDNA samples, which are mixtures of DNA fragments of both fetal and maternal origin. The authors already realized that the percentage of cfDNA with fetal origin varies between pregnancies, and the range is about 21.6% to 41% at the 3rd trimester. Given the high variation in fetal proportion in maternal plasma cfDNA, and the massive difference between placental methylome and blood methylome, it is almost mandatory to control for the fetal proportion when doing differential methylation analysis on maternal plasma cfDNA samples. There are plenty of examples in the literature about how to do differential analysis while controlling for proportions of different components in the samples.

There are also some minor problems in this manuscript.
First, the authors explained that when doing differential methylation analysis for MO vs Non-MO, they did not control for

ASD, because ASD is not a cause of MO (Methods section, lines 433-436). This justification is not valid. However, the authors may argue that the percentage of ASD patients is roughly the same in MO and Non-MO groups, hence not needed in a linear model. Nevertheless, it should be pointed out that including ASD as a covariate may still increase the power of the test. More importantly, if there is non-linear relation between ASD effect and MO effect, a stratification by ASD would be needed.
Second, the authors should be cautious about the interpretation of the PCA plots (Results section, lines 119-123). The separation of the two groups of samples is merely a consequence of doing PCA using differentially methylation regions, and the classification of the samples into two groups are obviously correlated with the various ASD scores.

Reviewer #3

(Remarks to the Author)
Williams et al present a study leveraging whole-genome bisulfite sequencing of maternal plasma cfDNA to identify epigenetic signatures associated with autism spectrum disorder (ASD) and maternal obesity (MO). The approach is novel and addresses a critical gap in non-invasive prenatal biomarker discovery. However, several methodological and interpretative issues need clarification before the work can be considered for publication.
Specific comments:
1. Provide details on possible pregnancy complications (e.g., preterm birth, intrauterine growth restriction) and whether these were controlled for. Since these conditions are known to have epigenetic signatures and they are also known to increase risk of ASD for the fetuses.
2. Were known chromosomal abnormalities or CNVs screened and excluded? These could confound methylation patterns.
3. While cfDNA is primarily placental in origin, could ASD pregnancies exhibit altered cfDNA composition (e.g., increased brain-derived cfDNA)? This can be evaluated using tissue-specific methylation signatures.
4. The strong sex-specific methylation patterns are intriguing. Were X chromosome regions included? If so, how do results change when X-linked loci are excluded?
5. Discuss biological plausibility for observed sex differences and whether they reflect developmental delay or other processes.
6. When identifying DMRs in placenta and cortex, were gestational ages matched?
7. Please provide directionality of methylation changes across tissues. Do they suggest developmental delay or accelerated maturation?
8. In Figure 3, clusters of placenta ASD DMRs on chromosomes 2, 5, 14 (males) and 2, 7, 12, 18, 21, 22 (females) raise concern for CNVs. Could these regions represent secondary effect of copy number changes rather than primary methylation changes?
9. Address whether CNV analysis was performed or if regions overlapping known CNVs were excluded.
10. While the overlap with SFARI genes is compelling, it would be interesting to further stratify by SFARI evidence level (gene score) to assess robustness.
11. Discuss whether shared pathways (e.g., synaptic signaling, circadian entrainment) are unique to ASD or reflect general neurodevelopmental processes.

Version 1:

Reviewer comments:

Reviewer #1

(Remarks to the Author)
The authors have addressed my concerns and the revisions to the manuscript are appropriate.

Reviewer #2

(Remarks to the Author)
The authors have addressed my previous comments on the earlier version of this manuscript, and explained why the major concern regarding the percentage of Fetal DNA in maternal cfDNA is hard and/or unnecessary to address. I understand that the authors may have limited resource to perform methylation based fetal fraction estimation in time. But the issue of fetal fraction is still too important to ignore. Given that the authors have tried seqFF to estimate fetal fraction and reported a relative low fetal fraction, my suggestion is:

Test if seqFF estimation of fetal fraction is correlated with chrY/chrX ratio based estimation of fetal fraction for samples with male babies. A good correlation would suggest that seqFF estimation can server as a marker for fetal fraction. The authors can then include seqFF estimation as a factor of interest and/or covariate in the analysis.

Reviewer #3

(Remarks to the Author)
The authors have adequately addressed all of my comments. I appreciate their extensive effort invested in the revision, and the manuscript has improved substantially. I have no further comments.

Version 2:

Reviewer comments:

Reviewer #2

(Remarks to the Author)
The authors performed the analysis suggested by this reviewer. The results are satisfactory. In particular, the fit of seqFF to chrY:chrX ratio (Supp Fig 31) makes sense, as fetal fraction should be almost twice of the Y/X ratio (as long as the fetal fraction is not too high). The offset of the fitted line can be explained by the unusually low Y/X ratio. The values of fetal fraction estimated by seqFF are also consistent with the fetal fraction reported in the literature (Supp Fig 30).
This revision addressed my remaining concerns. I recommend to accept this paper for publication.

Open Access This Peer Review File is licensed under a Creative Commons Attribution 4.0 International License, which permits use, sharing, adaptation, distribution and reproduction in any medium or format, as long as you give appropriate credit to the original author(s) and the source, provide a link to the Creative Commons license, and indicate if changes were made.
In cases where reviewers are anonymous, credit should be given to 'Anonymous Referee' and the source.
The images or other third party material in this Peer Review File are included in the article's Creative Commons license, unless indicated otherwise in a credit line to the material. If material is not included in the article's Creative Commons license and your intended use is not permitted by statutory regulation or exceeds the permitted use, you will need to obtain permission directly from the copyright holder.
To view a copy of this license, visit https://creativecommons.org/licenses/by/4.0/

Williams et al. 2025 – Reviewer Comments

We thank the editors and three reviewers for their time and helpful suggestions that have improved our manuscript. We were pleased with the overall enthusiasm for our study and its potential impact, as seen in comments such as “The study is important as it can inform minimally invasive prediction of patients at risk” (Rev 1) and “The approach is novel and addresses a critical gap in non-invasive prenatal biomarker discovery” (Rev 3). We appreciate the insightful suggestions for improvement and have revised the manuscript to improve clarity. In addition to changes to the manuscript’s text, we have also provided additional supplemental materials to support our findings.

For reference, here is a list of all modified materials:

- Supplemental Tables 1 (new information)
- Supplemental Tables 2 (new information)
- Supplemental Tables 3 (new analysis)
- Supplemental Tables 4 (new analysis)
- Supplemental Table 5 (new information)
- Supplemental Table 6 (new information)
- Supplemental Table 8 (new information)
- Supplemental Table 11 (new analysis)
- Supplemental Table 14 (new information)
- Supplemental Table 15 (new information)
- Supplemental Table 26 (new analysis)
- Supplemental Table 27 (new analysis)
- Supplemental Table 28 (new analysis)
- Supplemental Table 39 (new analysis)
- Supplemental Table 30 (new analysis)
- Supplemental Figure 2 (new analysis)
- Supplemental Figure 28 (new analysis)
- Supplemental Figure 30 (new analysis)
- Supplemental Figure 31 (new analysis)
- Supplemental Figure 32 (new analysis)
- Supplemental Figure 33 (new analysis)
- Removed former Supplemental Figures 2-4

We have provided a detailed point-by-point response addressing our reviewers’ feedback below, and a revised manuscript with changes noted in blue.

Reviewer #1 (Remarks to the Author):

The authors performed cfDNA methylation analysis to identify signatures of autism (ASD). The study is important as it can inform minimally invasive prediction of patients at risk. Methylation changes in the placenta and brain samples were intersected with those observed in blood and some biologically plausible pathways were implicated based on differential methylation patterns. The authors have also shared the data in the

Gene Expression Omnibus, providing an important resource to the community. There are however a few aspects that need to be addressed to allow better assessment of the robustness of findings:

1) The authors state "Significant DMRs were defined as those with a permutation p-value of < 0.05". Assuming hundreds of thousands of DMRs are tested for differential methylation, finding 1-2 thousands of DMRs does not seem unlikely by chance using nominal p-value cut-offs, regardless that permutations were used to calculate the p-values. Are there any significant findings after FDR correction?

Response: We appreciate the reviewer's focus on statistical level of evidence in the context of DMR identification. No single DMR was significant following FDR correction, but the conclusions in our title and abstract were not dependent on any individual DMR. To provide clarity on the background CpG clusters tested for association with ASD, we have expanded Supplemental Tables annotating the regions identified as potential DMRs for each DMRichR run (Supplemental Tables 5, 6, 8, 14-17). Our goal in this study was not to claim individual DMRs as high-confidence ASD- or MO-associated loci, but rather to identify reproducible gene pathway-level epigenomic signatures, and as such we focused our interpretation on enrichment results that passed FDR correction. We explicitly avoided overinterpretation of individual DMRs in our Discussion of our findings. It is crucial we make this approach clear, and to that end we have added additional language in the Discussion section to clarify the pitfalls of ascribing high confidence to individual DMRs discussed in this manuscript (lines 364-366).

2) Related to the question above about not having proving DMR level differences beyond what is expected by chance is the finding of the authors that there were only 377 DMRs when using all pregnancies (n=51), 1,436 ASD related DMRs in male (n=33) and 3,113 in female pregnancies (n=18) fits the idea of randomness because it is easier to get small permutations-based p-values when sample size is low than when sample size is large. Is the magnitude of DMR changes overall higher in female than in males pregnancies?

Response: While it undeniable that smaller sample sizes are more likely to produce small permutation based p-values in DMR analyses, there are also differences in the magnitude of female DMRs compared to male DMRs that supports the sex discrepancy as being biological. Specifically, the magnitude of the average ASD DMR in the female set is about 1.35% greater than the average ASD DMR in the male set, a difference that is statistically significant via Welch's t-test (p-value < 0.0001). This systemic difference in effect size is non-trivial in the context of differential methylation generally, and when applied across the tens of thousands of CpGs represented in our DMRs would constitute an appreciable, impactful change in DNA methylation with potential biological impact. This sex difference is also consistent with our recent publication on sex-stratified ASD DMRs from newborn blood spots in which ASD DMR methylation levels in females, for which we were better able to separate or cluster by diagnosis than males, even when male samples were downsampled to match[1] . We have highlighted this difference in magnitude between male and female cfDNA ASD DMRs with new

language in the Results section (lines 119-123), as well as in a new Supplemental Figure [Supplemental Figure 2].

3) The authors mention that samples were collected at 36-41 weeks, but I did not see a table showing differences in gestational age at sample collection between cases and controls and differences in maternal age. Both factors could affect methylation patterns and affect the results.

Response: To determine whether our reported cfDNA DMRs were driven by differences in maternal or gestational age between case and control groups, we first examined whether significant differences existed between sex-stratified case and control groups for the ASD and MO comparison. Sample-level data on maternal and gestational age (as well as other covariates) can be found in the updated Supplemental Table 1. The summary results of the Welch's t-tests performed to test for significant differences between case and control groups for the two variables of interest are provided below.

		Sex	
		Males (n = 33)	Females (n = 18)
Comparison	ASD vs. Control	14 ASD and 19 Control<br><br>Gestational Age: NS (P-value = 0.268)<br><br>Maternal Age: NS (P-value = 0.584)	10 ASD and 9 Control<br><br>Gestational Age: NS (P-value 0.865)<br><br>Maternal Age: NS (P-value = 0.2503)
	MO vs. Control	9 MO and 24 Control<br><br>Gestational Age: NS (P-value = 0.060)<br><br>Maternal Age: NS (P-value = 0.156)	9 MO and 9 Control<br><br>Gestational Age: NS (P-value = 0.709)<br><br>Maternal Age: NS (P-value = 0.810)

While none of these comparisons rise to the level of a significant difference between case and control group with regards to maternal or gestational age, some of the comparisons are approaching significance, particularly in the case of the Male MO DMR comparison. To further analyze this, we plotted heatmaps of the significance of each DMR in association with all available sample metadata and examined the fraction of DMRs significantly associated with maternal and gestational age to determine whether the identified DMRs show systemic association with maternal or gestational age.

Significance of the relationship was quantified as a p-value with t-test of the regression coefficient between each DMR and each covariate. Heatmaps are provided in Supplementary Figures 31-34.

As shown in these heatmaps, associations between maternal and gestational age and the reported cfDNA DMRs are uniformly weak and sparse, with $-\log_{10}$(p-values) remaining low and non-significant across most regions. In contrast, ASD and MO variables show structured and substantial association with their corresponding DMR groups. While maternal and gestational age may differ somewhat between sex-stratified outcome groups, these differences do not appear to exert systematic effects on the methylation signatures for ASD and MO reported here.

Given the importance of this question, we have added the heatmaps as Supplementary Figures and referenced them in the Methods section (lines 491-494).

4) Supplementary figures 2-4 can be removed because PCA plots based on features preselected because they seem most different between groups will always lead to distinct clusters when starting with a large number of initial features. The authors could start with randomly generated data for 100,000 DMRs and select top 1000 DMR or so which seem most different using a t-test p-values and can see that cluster differences will always emerge.

Response: We thank the reviewer for this insight. We have removed the referenced Supplementary Figures, as well as language in the Results section discussing them. Visualization of the associations between DMR methylation and all available sample metadata can now be found in Supplementary Figures 30-33.

Reviewer #2 (Remarks to the Author):

In this paper the authors studied the methylation changes in maternal plasma cfDNA between control and pregnancies with babies later diagnosed with ASD. The study is extensive, involving 51 samples and comparisons with placenta/brain data.

However, there is one major flaw in this study, which needs to be addressed. The samples studies in this paper are maternal plasma cfDNA samples, which are mixtures of DNA fragments of both fetal and maternal origin. The authors already realized that the percentage of cfDNA with fetal origin varies between pregnancies, and the range is about 21.6% to 41% at the 3rd trimester. Given the high variation in fetal proportion in maternal plasma cfDNA, and the massive difference between placental methylome and blood methylome, it is almost mandatory to control for the fetal proportion when doing differential methylation analysis on maternal plasma cfDNA samples. There are plenty of examples in the literature about how to do differential analysis while controlling for proportions of different components in the samples.

Response: We thank the reviewer for this important observation. Fetal fraction (FF) adjustment has been used historically in cfDNA research to try and facilitate tissue-specific attribution of signal to either the maternal or fetal component, and it was an intentional choice in the design of this study to not attempt FF correction when calling DMRs. The reasons for this are both practical and purpose-driven, as we will describe below.

Firstly, there remains serious uncertainty as to reliability of most common FF estimation tools in the context of bisulfite-converted libraries. All widely used FF estimation technologies assume as baseline the properties of native, non-bisulfite treated cfDNA[2,3]. Bisulfite conversion substantially alters and degrades cfDNA molecules, distorting many of the sequence characteristics required for these methods to function (e.g. fragment size distribution and GC content). This is illustrated in Supplemental Figure 29, where attempted SeqFF FF prediction results in an estimated fetal fraction far lower than what has been observed historically for 3rd trimester cfDNA[4]. While it would be theoretically possible to estimate FF without relying on methods trained on native cfDNA characteristics by identifying paternal-specific SNPs present in the cfDNA data, this approach was not feasible for our study given that the MARBLES study does not collect paternal biospecimens[5]. To our knowledge, no validated FF estimation method exists for bisulfite-converted cfDNA WGBS data without paternal genotyping available.

Secondly, our foremost objective in this study was “To test the efficacy of cfDNA methylomes as environmentally-sensitive predictors of ASD *in-utero*” (Introduction, Lines 89-90). We attempted to investigate whether the cfDNA methylome itself, without respect to its specific maternal or fetal components, showed patterns of variation in association with ASD as well as an environmental factor for ASD (MO). The interrogation of the characteristics of cfDNA as an aggregate tissue has already shown exceptional utility in the detection of fetal aneuploidy, which does not require fetal fraction correction to accurately predict trisomy with count-based methods[6]. Considering this, our analysis attempts to identify epigenetic markers of a potential connection between MO and ASD, considering both fetal and maternal contributions to cfDNA as informative biological signal.

With this in mind, we were careful throughout to paper to explicitly define our material of interest as cell-free DNA (cfDNA) as opposed to cell-free fetal DNA or cell-free maternal DNA. We also do not make any claims regarding the potential fetal or maternal specificity of the detected DMRs.

This reviewer’s comments on how our study approached fetal fraction are important, and we have revised our manuscript to better explain our methods and the limitations that accompany them. You can find revised language on this point in the Discussion (lines 351-357) and Methods (lines 447-449) sections.

There are also some minor problems in this manuscript.
First, the authors explained that when doing differential methylation analysis for MO vs

Non-MO, they did not control for ASD, because ASD is not a cause of MO (Methods section, lines 433-436). This justification is not valid. However, the authors may argue that the percentage of ASD patients is roughly the same in MO and Non-MO groups, hence not needed in a linear model. Nevertheless, it should be pointed out that including ASD as a covariate may still increase the power of the test. More importantly, if there is non-linear relation between ASD effect and MO effect, a stratification by ASD would be needed.

Response: There may have been some confusion about when obesity was measured. MO was determined by a mother's pre-pregnancy BMI, not their BMI during the pregnancy itself. Because maternal BMI was measured pre-pregnancy, it is temporally independent of ASD diagnosis.

Nevertheless, we did investigate whether these were significant differences in the percentage of ASD participants between the MO and Control groups and found no significant differences to exist (Fisher's Exact Test, p-value = 0.777).

While evidence of a non-linear relationship between ASD and MO would have forced ASD stratification or correction in our MO DMR models, there is an ample body of evidence that the relationship between MO and ASD is monotonic[7–10]. This is especially true when considering participants with maternal BMI > 18.5, which describes all but one of our study participants. Supported by prior publications, we assumed a linear relationship.

Second, the authors should be cautious about the interpretation of the PCA plots (Results section, lines 119-123). The separation of the two groups of samples is merely a consequence of doing PCA using differentially methylation regions, and the classification of the samples into two groups are obviously correlated with the various ASD scores.

Response: We thank the reviewer for this insight. We have removed the referenced Supplementary Figures, as well as language in the Results section discussing them. Visualization of the associations between DMR methylation and all available sample metadata can now be found in Supplementary Figures 30-33.

Reviewer #3 (Remarks to the Author):

Williams et al present a study leveraging whole-genome bisulfite sequencing of maternal plasma cfDNA to identify epigenetic signatures associated with autism spectrum disorder (ASD) and maternal obesity (MO). The approach is novel and addresses a critical gap in non-invasive prenatal biomarker discovery. However, several methodological and interpretative issues need clarification before the work can be considered for publication.
Specific comments:
1. Provide details on possible pregnancy complications (e.g., preterm birth, intrauterine

growth restriction) and whether these were controlled for. Since these conditions are known to have epigenetic signatures and they are also known to increase risk of ASD for the fetuses.

Response: This reviewer raises an insightful point regarding the potential impact of pregnancy complications on cfDNA and placenta epigenetics. We have updated Supplemental Table 1 to reflect both the gestational age of the fetuses at time of birth as well as whether the mothers involved in this study were diagnosed with intrauterine growth restriction (IUGR). In summary, two of our 51 participant births would qualify as pre-term and 50 of our 51 participant mothers were not diagnosed with IUGR. It is unknown whether the one remaining participant mother was or was not diagnosed with IUGR, as that participant did not provide the MARBLES study her pregnancy records. Given the near-complete absence of pre-term birth and IUGR diagnosed participants in our participants, we did not elect to correct for these conditions as covariates during the DMR identification process. A visualization of how DMR methylation associates with Gestational Age at Delivery can now be found in Supplemental Figures 30-33.

2. Were known chromosomal abnormalities or CNVs screened and excluded? These could confound methylation patterns.

Response: No, our MARBLES population was not genetically screened nor made to exclude participants based on known CNVs or chromosomal abnormalities. As such, methylation values for any given DMR in any single participant could be the results of both genetic and environmental contributions. While it would certainly be informative to have data on whether any of our participants are positive for common ASD-associated aneuploidies and CNVs (i.e. sex chromosome aneuploidies, 15q11-q13 duplication, etc), ultimately our focus was on identifying a broad signature of epigenetic dysregulation associated with ASD and MO against the background of enhanced ASD likelihood presented by the MARBLES cohort. ASD is highly heterogenous, and no single mutation or CNV has been found to account for more than 1% of ASD cases[11]. In the face of this extreme heterogeneity, we took care in presenting our DMRs as being associated with ASD or MO without claiming any observed epigenetic variance to be due to environmental versus genetic causes. Our observed methylation signatures exist at the interface of both genetic and environmental contributions, and we have made efforts to present them as such in the manuscript. To clarify this point, we have added a new line in the Methods section explicitly stating that no efforts were made to exclude samples (lines 397-400).

3. While cfDNA is primarily placental in origin, could ASD pregnancies exhibit altered cfDNA composition (e.g., increased brain-derived cfDNA)? This can be evaluated using tissue-specific methylation signatures.

Response: While it is possible that ASD pregnancies exhibit atypical cfDNA contributions from the brain, it has been extensively characterized in prior literature that the fetal fraction of cfDNA is derived from placental trophoblast cells[12–16]. The similarities in DMRs observed between cfDNA and brain are likely the result of the well-

characterized similarities in placenta methylation profiles and brain methylation profiles more broadly[17–19].

4. The strong sex-specific methylation patterns are intriguing. Were X chromosome regions included? If so, how do results change when X-linked loci are excluded?

Response: X-chromosome regions were not excluded from DMR analysis, though statistical analysis of the cfDNA ASD DMRs does not indicate an enrichment for X-linked genes (Fisher's Exact Test, p-value = 1 for both males and females). Removal of all X-linked DMRs from consideration would result in the depletion of 40 DMRs (~3%) from the male cfDNA ASD DMR set and 157 DMRs (~5%) from the female cfDNA ASD DMR set, which is highly unlikely to change the results of downstream enrichment analysis. Furthermore, our reported sex-differential methylation patterns in autosomal cfDNA concurs with prior epigenome-wide analyses of blood, a phenomenon shown to persist even after correction for cell type composition[20,21].

5. Discuss biological plausibility for observed sex differences and whether they reflect developmental delay or other processes.

Response: We have expanded our discussion of the potential biological causes for the observed sex differences in cfDNA ASD DMRs (lines 294-309). We have also added a Supplemental Figure (Supplemental Figure 28) showing differences in KEGG pathway enrichments between the subset of DMRs in similar genic elements with altered methylation directionality between fetal sexes (Supplemental Figure 28A) and the subset of DMRs in similar genic elements with concordant methylation directionality between fetal sexes (Supplemental Figure 28B). This analysis is used to discuss the underlying biological pathways which show differential methylomic signatures in association with ASD between fetal sexes.

6. When identifying DMRs in placenta and cortex, were gestational ages matched?

Response: While comparing our placental ASD DMRs to ASD DMRs from gestational age-matched post-mortem cortex samples would be the gold-standard for cross tissue epigenetic comparison, the absence of gestational post-mortem ASD cortex samples makes this comparison not possible in humans. This is a microcosm of a systemic under-availability of post-mortem ASD brain discussed in prior articles[22]. With this in mind, when generating post-mortem cortex ASD DMRs from previously published data[23], we did correct for age at time of death.

7. Please provide directionality of methylation changes across tissues. Do they suggest developmental delay or accelerated maturation?

Response: Global directionality of methylation changes in association with ASD and MO are provided in Figures 2, 3, and 5. DMR-level methylation directionality can be found in Supplemental Tables 5, 6, and 14-17.

To investigate whether our DMRs suggest developmental delay or accelerated maturation, we identified which of our placenta and cortex DMRs overlapped with CpGs annotated in placental gestational age epigenetic clocks and cortex age epigenetic clocks[24,25]. No such comparison could be made for maternal blood cfDNA DMRs due to the absence of any published epigenetic clock.

Amongst our reported sex-stratified placenta ASD DMRs there was minimal overlap with the gestational age associated CpGs reported in Lee *et al.* 2019. Only two of the male placenta ASD DMRs and ten of the female placenta ASD DMRs overlapped with the 558 CpG sites predictive of gestational age in the reported model. Though most of the placental DMRs (7/12) supported a model of developmental delay correlating with ASD DMRs (i.e. a DMR that is hypermethylated in ASD pregnancies being annotated to a CpG that is demethylated as gestational age increases), the minute sample size of DMRs that also overlapped the CpG sites known to predict gestational age makes confident interpretation of these results unreliable.

Overlaps between our cortex ASD DMRs and the cortical age-predicting CpGs of Shireby et al. 2020 were even more sparse. A single male cortex ASD DMR and three female cortex ASD DMRs overlapped with the 347 CpGs reported in the cortical age prediction model. In contrast to the placenta results, three of the four DMRs supported a model of accelerated maturation correlating with the ASD DMRs, with a single DMR in the female set suggesting developmental delay in association with ASD's epigenetic profile.

While the overlaps between our DMRs and published epigenetic clocks are too minor to make any definite conclusions about whether methylation changes in ASD patient tissues correspond to developmental delay or accelerated maturation broadly, we have included the annotations for the overlapping DMRs in Supplemental Table 26 and added new language in the Methods section (lines 498-500) to address this.

8. In Figure 3, clusters of placenta ASD DMRs on chromosomes 2, 5, 14 (males) and 2, 7, 12, 18, 21, 22 (females) raise concern for CNVs. Could these regions represent secondary effect of copy number changes rather than primary methylation changes?

Response: This reviewer raises a great point about the potential for CNVs to influence DMR calls, as coverage shifts caused by CNVs could result in the artificial skew of weight given to effected CpGs during the region identification process. This would be particularly impactful if CNVs were differently enriched between comparison groups.

To test whether this was the case, we quantified per-sample CpG coverage across all reported placenta ASD DMRs (2,678 male and 8,447 female) and compared average regional coverage between ASD and Control groups using t-tests with FDR correction [Supplemental Tables 27 and 28]. If CNVs were a major contributor to the observed methylation differences, we would expect a substantial proportion of DMRs to exhibit significant group-level differences in sequencing coverage.

However, we observed no DMRs showing significant coverage differences between ASD and control groups at an FDR threshold of $q < 0.05$. Even using a more permissive nominal threshold ($p < 0.05$), only 36 of 2,678 male ASD DMRs and 2,238 of 8,447 female ASD DMRs exhibited modest coverage differences between groups. Upon testing for overrepresentation of any chromosomes among this set of nominally significant DMRs via Fisher's Exact Test, no significant enrichments were identified in the male DMR set [Supplemental Table 29]. Significant enrichments were identified in the female DMR sets for chromosomes 3, 5, 10, 16, 17, and 21 [Supplemental Table 30]. However, these enrichments do not reflect the contiguous large-scale differences one would expect to observe in response to CNV-driven variance. Furthermore, the coordinates of these enrichments do not implicate many of the most well-characterized ASD-associated CNVs, such as 15q11-q13 duplication, 22q11 deletion and duplication, and 7q11.23 duplication[26].

Taken together, these results indicate that the identified placenta DMRs are not driven by systematic group-level differences in sequencing coverage and are thus unlikely to represent secondary effects of clustered CNVs. We have added new Supplemental Tables 27-30 and new wording to the Methods section to highlight this point (lines 500-506).

9. Address whether CNV analysis was performed or if regions overlapping known CNVs were excluded.

Response: While the question of whether CNVs or other varieties of genetic variants associate with specific epigenetic profiles in ASD patients is fascinating, it is beyond the scope of this exploratory study. Shaped in part by the logistical challenges of acquiring a sufficient number of blood plasma samples from a prospective ASD cohort with known diagnostic outcomes and pre-pregnancy maternal BMI, we designed this study to identify potential epigenetic markers of ASD and MO in cfDNA from the broadest possible swath of participants. As such, we did not exclude participants from the study based on the presence of absence of genetic variants implicated in ASD, nor did we exclude DMRs from our analyses if they overlapped with regions previously reported to harbor CNVs. Future studies examining which of our reported DMRs may only be associated with ASD or MO in a genetic context of specific CNVs overlapping the DMRs would be compelling and illuminate whether our reported ASD and MO DMRs remain significantly associated with the respective outcomes against a narrower genetic background. We have added language clarifying this in the Methods section (lines 460-463).

10. While the overlap with SFARI genes is compelling, it would be interesting to further stratify by SFARI evidence level (gene score) to assess robustness.

Response: We would like to thank the reviewer for this comment, as it led to additional analysis that has improved our manuscript. Fisher's Exact Tests for SFARI subcategory enrichment were performed on all cfDNA DMRs, and the results of these analyses are

described in Supplemental Table 11. In short, an enrichment for SFARI 2 (strong candidate) genes appears to be the most significant SFARI enrichment for male ASD cfDNA DMR genes, female ASD cfDNA DMR genes, and male MO cfDNA DMR genes. The female ASD cfDNA DMR gene list also shows a significant enrichment for SFARI 1 (high confidence) genes. This supports the robustness of our reported associations between these DMR groups and the SFARI gene database, as it shows the existing overlaps to be in high confidence and strong candidate genes, as opposed to genes with solely suggestive evidence regarding their role in ASD etiology.

We have included new language in the Results section (lines 145-148 and 236-239) to highlight this new analysis.

11. Discuss whether shared pathways (e.g., synaptic signaling, circadian entrainment) are unique to ASD or reflect general neurodevelopmental processes.

Response: Our thanks to the reviewer for pointing out this gap in the original manuscript's Discussion section! We have added to the Discussion section (lines 281-289) to elaborate on the specificity of the implicated pathways to ASD as opposed to more general neurodevelopment.

References:

1. Mouat, J. S. *et al.* Sex-specific DNA methylation signatures of autism spectrum disorder from whole genome bisulfite sequencing of newborn blood. *Biol. Sex Differ.* **16**, 30 (2025).
2. Kim, S. K. *et al.* Determination of fetal DNA fraction from the plasma of pregnant women using sequence read counts: Determination of fetal DNA fraction from the plasma of pregnant women using sequence read counts. *Prenat. Diagn.* **35**, 810–815 (2015).
3. Straver, R., Oudejans, C. B. M., Sistermans, E. A. & Reinders, M. J. T. Calculating the fetal fraction for noninvasive prenatal testing based on genome-wide nucleosome profiles. *Prenat. Diagn.* **36**, 614–621 (2016).

4. Sun, K. *et al.* Plasma DNA tissue mapping by genome-wide methylation sequencing for noninvasive prenatal, cancer, and transplantation assessments. *Proc. Natl. Acad. Sci.* **112**, (2015).
5. Lo, Y. M. D. *et al.* Maternal Plasma DNA Sequencing Reveals the Genome-Wide Genetic and Mutational Profile of the Fetus. *Sci. Transl. Med.* **2**, (2010).
6. Fan, H. C., Blumenfeld, Y. J., Chitkara, U., Hudgins, L. & Quake, S. R. Noninvasive diagnosis of fetal aneuploidy by shotgun sequencing DNA from maternal blood. *Proc. Natl. Acad. Sci.* **105**, 16266–16271 (2008).
7. Wang, Y., Tang, S., Xu, S., Weng, S. & Liu, Z. Maternal Body Mass Index and Risk of Autism Spectrum Disorders in Offspring: A Meta-analysis. *Sci. Rep.* **6**, 34248 (2016).
8. Reynolds, L. C., Inder, T. E., Neil, J. J., Pineda, R. G. & Rogers, C. E. Maternal obesity and increased risk for autism and developmental delay among very preterm infants. *J. Perinatol.* **34**, 688–692 (2014).
9. Li, M. *et al.* The Association of Maternal Obesity and Diabetes With Autism and Other Developmental Disabilities. *Pediatrics* **137**, e20152206 (2016).
10. Krakowiak, P. *et al.* Maternal Metabolic Conditions and Risk for Autism and Other Neurodevelopmental Disorders. *PEDIATRICS* **129**, e1121–e1128 (2012).
11. Jeste, S. S. & Geschwind, D. H. Disentangling the heterogeneity of autism spectrum disorder through genetic findings. *Nat. Rev. Neurol.* **10**, 74–81 (2014).
12. Tjoa, M. L., Cindrova-Davies, T., Spasic-Boskovic, O., Bianchi, D. W. & Burton, G. J. Trophoblastic Oxidative Stress and the Release of Cell-Free Feto-Placental DNA. *Am. J. Pathol.* **169**, 400–404 (2006).

13. Chan, K. C. A. *et al.* Size Distributions of Maternal and Fetal DNA in Maternal Plasma. *Clin. Chem.* **50**, 88–92 (2004).

14. Flori, E. Circulating cell-free fetal DNA in maternal serum appears to originate from cyto- and syncytio-trophoblastic cells. Case report. *Hum. Reprod.* **19**, 723–724 (2004).

15. Alberry, M. *et al.* Free fetal DNA in maternal plasma in anembryonic pregnancies: confirmation that the origin is the trophoblast. *Prenat. Diagn.* **27**, 415–418 (2007).

16. Masuzaki, H. Detection of cell free placental DNA in maternal plasma: direct evidence from three cases of confined placental mosaicism. *J. Med. Genet.* **41**, 289–292 (2004).

17. Zhu, Y. *et al.* Placental DNA methylation levels at CYP2E1 and IRS2 are associated with child outcome in a prospective autism study. *Hum. Mol. Genet.* **28**, 2659–2674 (2019).

18. Diez-Ahijado, L. *et al.* Evaluating the association between placenta DNA methylation and cognitive functions in the offspring. *Transl. Psychiatry* **14**, 383 (2024).

19. Ravaei, A., Emanuele, M., Nazzaro, G., Fadiga, L. & Rubini, M. Placental DNA methylation profile as predicting marker for autism spectrum disorder (ASD). *Mol. Med.* **29**, 8 (2023).

20. Singmann, P. *et al.* Characterization of whole-genome autosomal differences of DNA methylation between men and women. *Epigenetics Chromatin* **8**, 43 (2015).

21. Grant, O. A., Wang, Y., Kumari, M., Zabet, N. R. & Schalkwyk, L. Characterising sex differences of autosomal DNA methylation in whole blood using the Illumina EPIC array. *Clin. Epigenetics* **14**, 62 (2022).

22. Fetit, R., Hillary, R. F., Price, D. J. & Lawrie, S. M. The neuropathology of autism: A systematic review of post-mortem studies of autism and related disorders. *Neurosci. Biobehav. Rev.* **129**, 35–62 (2021).

23. Vogel Ciernia, A. *et al.* Epigenomic Convergence of Neural-Immune Risk Factors in Neurodevelopmental Disorder Cortex. *Cereb. Cortex* **30**, 640–655 (2020).

24. Lee, Y. *et al.* Placental epigenetic clocks: estimating gestational age using placental DNA methylation levels. *Aging* **11**, 4238–4253 (2019).

25. Shireby, G. L. *et al.* Recalibrating the epigenetic clock: implications for assessing biological age in the human cortex. *Brain J. Neurol.* **143**, 3763–3775 (2020).

26. Vicari, S. *et al.* Copy number variants in autism spectrum disorders. *Prog. Neuropsychopharmacol. Biol. Psychiatry* **92**, 421–427 (2019).

Williams et al. 2025 – Reviewer Comments

We are grateful for the comments provided by all three reviewers and believe the manuscript to be much improved by their insights. While Reviewers #1 and #3 are fully satisfied by our revisions, we recognize that Reviewer #2 has outstanding concerns regarding fetal fraction estimation. In addition to the previously discussed changes to the manuscript, we have further revised our language and provided additional supplemental materials to support our findings.

For reference, here is a list of all materials modified from our last submission:

- Supplemental Table 1 (new information)
- Supplemental Table 2 (new information)
- Supplemental Table 25 (new information)
- Supplemental Table 26 (new information)
- Supplemental Table 27 (new information)
- Supplemental Table 28 (new information)
- Supplemental Figure 6 (new analysis)
- Supplemental Figure 31 (new analysis)
- Supplemental Figure 32 (new analysis)
- Supplemental Figure 33 (new analysis)
- Supplemental Figure 34 (new analysis)
- Supplemental Figure 35 (new analysis)
- Supplemental Figure 36 (new analysis)
- Supplemental Figure 37 (new analysis)
- Supplemental Figure 38 (new analysis)
- Supplemental Figure 39 (new analysis)

We have provided a detailed point-by-point response addressing our reviewers' feedback below, and a revised manuscript with changes from our previous submission noted in blue.

Reviewer #1 (Remarks to the Author):

The authors have addressed my concerns and the revisions to the manuscript are appropriate.

Response: We thank this reviewer for their feedback and recommended revisions to the manuscript.

Reviewer #2 (Remarks to the Author):

The authors have addressed my previous comments on the earlier version of this manuscript, and explained why the major concern regarding the percentage of Fetal DNA in maternal cfDNA is hard and/or unnecessary to address. I understand that the authors may have limited resource to perform methylation based fetal fraction estimation in time. But the issue of fetal fraction is still too important to ignore. Given

that the authors have tried seqFF to estimate fetal fraction and reported a relative low fetal fraction, my suggestion is:

Test if seqFF estimation of fetal fraction is correlated with chrY/chrX ratio based estimation of fetal fraction for samples with male babies. A good correlation would suggest that seqFF estimation can server as a marker for fetal fraction. The authors can then include seqFF estimation as a factor of interest and/or covariate in the analysis.

Response: We recognize the impact that fetal fraction (FF) can have on methylomic analysis, and we appreciate this reviewer providing feasible suggestions on how to proceed.

While we maintain reservations about using atypically low SeqFF estimates of FF as an adjusted-for covariate during DMR identification, we have performed new DMR analysis that include SeqFF-estimated FF as an adjusted-for covariate to determine the magnitude of this correction's impact on the resulting identified regions. While our SeqFF estimates do not perfectly correlate with ChrY:ChrX read ratio in males, they do demonstrate a reliably positive correlation and may roughly track actual FF even if not accurate on a per-sample basis (Supplemental Figure 31, Supplemental Tables 1 and 2).

In summary, the SeqFF-adjusted DMRs (Supplemental Tables 26-29) did not appreciably deviate from the previously identified DMRs in any manner that relates to our conclusions described in the manuscript. The SeqFF-adjusted DMRs showed significantly greater overlap of both gene lists and genomic regions with their unadjusted DMR counterparts (Supplemental Figures 32-37). SeqFF-adjusted ASD DMR gene lists maintained overlapping enrichments for KEGG pathways involved in synapse development and cell signaling seen in the original DMRs (Supplemental Figure 38), and maintain significant overlap with the placenta and cortex DMR genes (Supplemental Figure 39).

One of the only discrepancies between the original cfDNA DMRs and the SeqFF-adjusted DMRs is in the results of gene level overlap analyses with the SFARI Gene list. Specifically, the SeqFF-adjusted female cfDNA ASD DMRs no longer show a significant enrichment ($p = 0.13$) for the SFARI gene list, and the SeqFF-adjusted female cfDNA MO DMRs do show a significant enrichment ($p = 2.4 \times 10^{-3}$) for the SFARI gene list. To appropriately caution readers of this fact, we have highlighted this finding explicitly in lines 145-146 and lines 239-241.

Given the minimal impact of adjustment for SeqFF on our primary conclusions, in combination with our continued skepticism of SeqFF's ability to accurately estimate fetal fraction from WGBS data, we remain confident in our reporting of the cfDNA DMRs described in this manuscript. However, we have added the coordinates and annotations of all SeqFF-adjusted cfDNA DMRs to the Supplemental Tables, and encourage any researchers interested in following-up on this work to examine whichever set of DMRs they conclude to be most relevant to their research interests.

Reviewer #3 (Remarks to the Author):

The authors have adequately addressed all of my comments. I appreciate their extensive effort invested in the revision, and the manuscript has improved substantially. I have no further comments.

Response: We thank this reviewer for their feedback and recommended revisions to the manuscript.
